# Assessing delimiting strategies to identify the infested zones of quarantine plant pests and diseases

**DOI:** 10.1038/s41598-025-90343-2

**Published:** 2025-02-15

**Authors:** Jun Min Joshua Koh, Nik J. Cunniffe, Stephen Parnell

**Affiliations:** 1 School of Life Sciences, The University of Warwick, Coventry, CV4 7AL UK; 2 Department of Plant Sciences, University of Cambridge, Cambridge, CB2 3EA UK

**Keywords:** Delimiting surveys, Individual-based model, Plant epidemiology, Huanglongbing, Plant pest management, Statistical methods, Ecological modelling, Ecological epidemiology

## Abstract

Following the discovery of a quarantine plant pest or disease, delimitation is urgently conducted to define the boundaries of the infested area, typically through surveys that detect the presence or absence of the pest. Swift and accurate delimitation is crucial after a pest or pathogen enters a new region for containment or eradication. Delimiting an area that is too small allows the pest to spread uncontrollably, while delimited areas that are too large can lead to excessive economic costs, making eradication cost-prohibitive. Despite its significance, there is a lack of comprehensive reviews on delimiting strategies and their effectiveness in managing plant pests; many current practices are ad-hoc and not scientifically based. In this study, we used an individual-based model to simulate the spread of Huanglongbing (citrus greening), a priority EU pest, and evaluated three delimiting strategies across various host distribution landscapes. We found that an adaptive strategy was most effective, especially when tailored to the polycyclic nature of the pest. This underscored the need for specific delimiting approaches based on the epidemiological characteristics of the target pest.

**Supplementary Information:**

The online version contains supplementary material available at 10.1038/s41598-025-90343-2.

## Introduction

Plants play a crucial role in tackling major social and environmental challenges today, such as the provision of food, fuel and environmental stability. However, the threat posed by invasive plant pests and pathogens is substantial^1,2^, and continually rising due to increasing globalization and climate change^3^. Epidemics resulting from these pest invasions can have catastrophic impacts on human health, food security and contribute to environmental change. For example, after *Xylella fastidiosa* was detected in Italy in 2013, it reduced ecosystem services including food production, soil erosion regulation and ornamental resources by 34%, and decreased biodiversity by 28%^4^. This was due to its impact on high-value habitats and the genetic diversity of the host olive trees, as farmers mostly replanted resistant cultivars. Moreover, in Italy the pathogen has been projected to lead up to 5.2 billion Euros in economic losses on olive alone and can infect hundreds of other plant species^5^.

After discovering a plant pest – which we interpret broadly to refer to vectors, herbivorous insects, and pathogens^6^ – in a new area, the extent of the infestation must be quickly determined so control measures can be implemented to prevent further spread. In the EU, delimitation, which involves defining the boundaries of an area considered to be infested by a pest (i.e. the potential infested zone or PIZ)^2^, is a regulatory requirement for all quarantine pests^7^. It is typically conducted via surveys which generate information on the observed presence and absence of a pest within a population^8^. An example of a delimiting strategy involves drawing a circle around every identified infected (or infested, in the case of an insect pest) individual and supplementing each circle with a buffer zone^9–11^. To achieve eradication or containment of a pest, the chosen delimiting strategy should delimit an area that contains the infestation^12^.

Understanding early-stage pest spread is challenging and depends on the epidemiological characteristics of the pest, the landscape, and environmental conditions at the initial arrival site. Spatial and temporal processes of surveillance and detection (e.g. the frequency and intensity of surveys, and the sensitivity of detection methods used) also determine how long the pest has been left undetected. A lack of understanding of these complex interacting processes has contributed to a lack of consensus on how to effectively conduct delimitation. Consequently, while there has been studies that evaluated previously applied strategies^13–15^ and documents that provide guidelines on the design and implementation of delimiting strategies^16,17^, there is little overview on the effectiveness of different delimiting strategies in the domain of plant pests and real-world approaches are often ad-hoc and not science-based. This is concerning as failure to accurately delimit an outbreak could result in either delimiting an area that is too small, which could lead to the pest spreading further, or an area that is too large, which could lead to unnecessary costs and potential legal obstacles associated with control programs^18^. Effective delimitation is especially important in non-agricultural areas (e.g. plants in urban areas, or natural communities) where host populations are not intensively managed and inspected. These areas can act as important reservoirs for pests and influence the connectivity between commercial and conservation areas^19–24^.

To address the knowledge gap and provide scientific support for policymakers, we used an individual-based model (IBM) to simulate the spread of *Candidatus* Liberibacter spp. - the causal agent of Huanglongbing (HLB), also known as citrus greening. We then evaluated the performance of three delimiting strategies across various host distribution landscapes with host densities comparable to those of urban areas in Spain. The work was developed in collaboration with the European Food Safety Authority (EFSA) plant pest survey methods Working Group to inform and update the EFSA General guidelines for statistically sound and risk-based surveys of plant pests^16^.

We chose to model HLB as it is a pertinent example due to its economic significance and its current threat to global citrus-producing areas, including Europe. It is caused by the bacterium *Candidatus* Liberibacter spp., and is considered to be the most destructive citrus pathosystem in the world^25^ and is an EU “priority plant pest”^26^. Previously confined to Asia and Africa, it was first detected in the Western Hemisphere in 2004 in Brazil, where in just five years it had infected approximately four million trees^27^. The effects of HLB were also devastating after its introduction in Florida in 2005 where after progressing slowly in the first three years, the disease incidence quickly started doubling every year until reaching 80% by 2013^28^ and generated losses estimated at approximately US$1 billion per year^29^. While HLB is currently absent in Europe, two of its vectors *Trioza erytreae* and *Diaphorina citri* have been detected in the Mediterranean Basin. *T. erytreae* was first detected in the island of Madeira (Portugal) in 1994, then in the Canary Islands (Spain) in 2002, and eventually on the mainland of Spain in Galicia in 2014^30^, while *D. citri* was recently detected in Cyprus in 2023^31^. Given that Spain is the fifth largest producer of citrus in the world^32^, the detection of *T. erytreae* in Spain has instigated the need for a contingency plan which includes an effective delimitation strategy. Delimitation for HLB is particularly challenging given its long asymptomatic period and delayed but rapid rate of population growth^33^. While most citrus trees grow in commercial groves and orchards, substantial populations are also found in municipal and residential areas, where tree density and distribution differ significantly.

## Methodology

### Host distribution methods

**Fig. 1:**
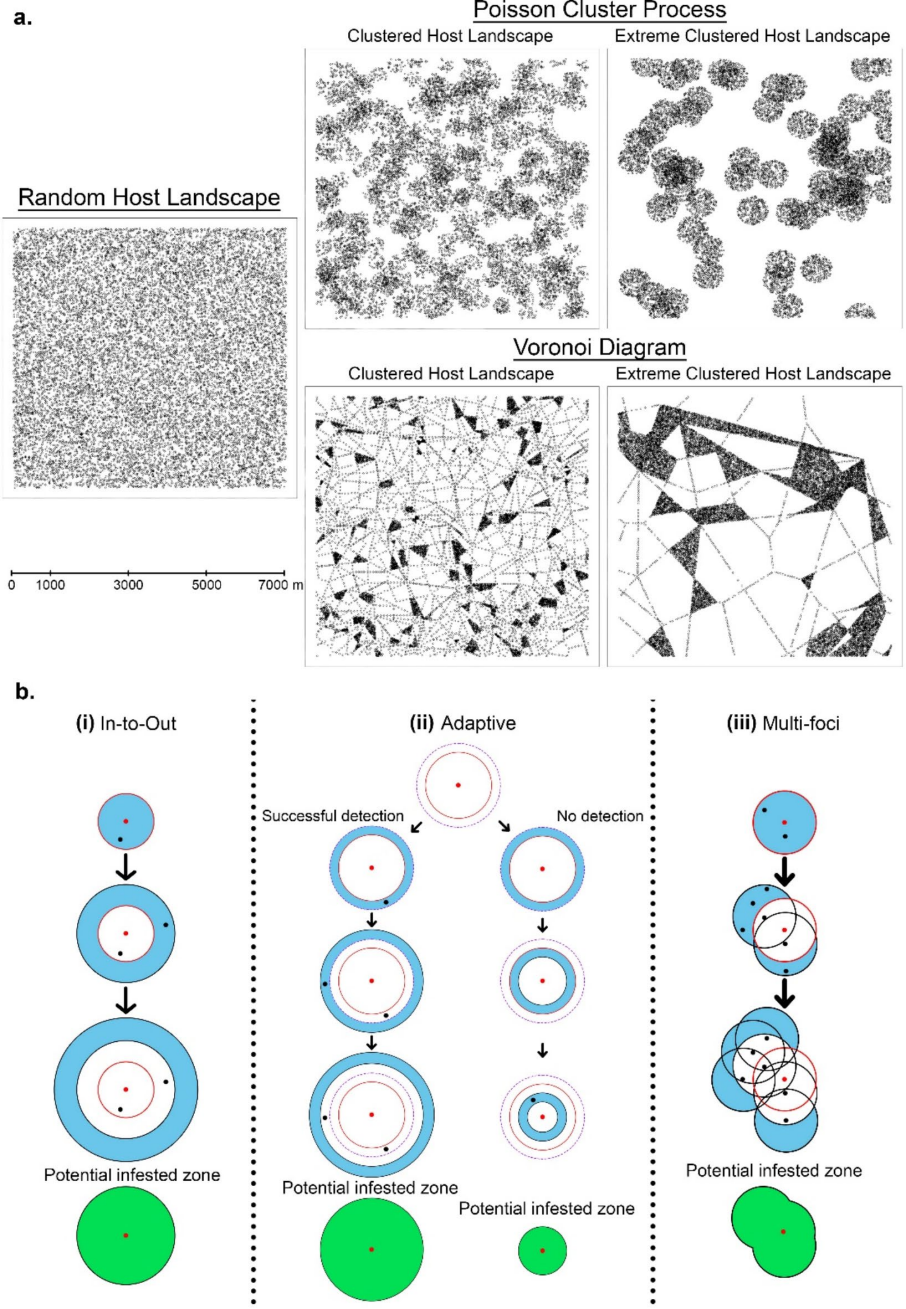
Examples of all the different host landscape types. Each plot has the same area and number of points (15,941) (**a**). Diagram of how the three delimiting strategies work. The red circle represents the first circle drawn around the first infected tree based on each strategy’s calculated radius length. The band in between the red circle and dotted purple circle in the Adaptive strategy represents the band in which the preliminary survey is conducted. Red dots represent the first infected tree detected, black dots represent subsequent detections of infected trees and survey bands are indicated by blue areas (**b**).

We used three different methods to simulate the spatial distribution of hosts which represented a range of landscape types (Fig. 1a). The spread of HLB through the different host landscapes was modelled with an individual based model (IBM). The IBM was coded in R 4.3.3^34^. We test our model using a landscape with a similar density of citrus trees to that of Seville, a large city in Spain. It is difficult to unambiguously quantify the number of citrus trees in Seville, with estimates ranging from 25,000 bitter orange trees in 1996^35^ to 50,000 orange trees in 2020^36^. In line with these estimates, here we used the value quoted by Galvañ, et al.^37^, who estimated there are approximately 46,000 citrus trees in the city of Seville which has an area of 141.4 km^2^. To approximate the tree density in Seville, the IBM generates 15,941 trees and distributes them in a 49 km^2^ plot.

The first method involves random placement, where x and y coordinates were drawn from a uniform probability distribution between 0 and 7000. This resulted in points with randomly generated coordinates that were uniformly distributed and exhibited no distinct pattern or clustering. The second method used the R package spatstat^38^ to generate a clustered host landscape, using a Poisson cluster process. The final method captured the key characteristics of urban citrus tree distribution in Seville, where trees tend to be distributed along roadsides and in parks. Firstly, to avoid artifacts at the boundaries of the plot, a Voronoi diagram that extended beyond the 7 km x 7 km plot was constructed and an augmented population size was chosen (28,000). Using an estimate of Seville’s public parks and gardens and acknowledging that not all trees are citrus, we randomly selected 8% of polygons to represent these areas. Within each polygon, we simulated a certain number of trees based on its area and distributed them randomly. The remaining trees were then distributed along the lengths of all the remaining polygons to simulate tree plantings along roads. To introduce more spatial host heterogeneity, the spacing between trees was varied by first calculating the average spacing between each tree necessary to populate the lengths of all the remaining polygons. We then randomly chose a value between 0.8 and 1.4 times of the average spacing for each polygon length and iterated this process until the difference between the simulated and augmented population sizes was less than or equal to 10. Finally, the entire plot was cropped down to 7 km x 7 km and trees were randomly removed to achieve the target population of 15,941. We also simulated landscapes with an unrealistic level of clustering (Extreme clustered landscapes) with both the Poisson cluster process and Voronoi diagram methods to fully test the robustness of the delimiting strategies. With the three different distribution methods and two levels of clustering, there was a total of five different host landscape types (Fig. 1a).

To ensure there was enough contrast between the host landscape types, we generated 500 realizations of each landscape type, simulated a 5-year epidemic with the IBM and calculated the disease prevalence per year and the number of susceptible and infectious hosts in the population in each year. For each realization, we ensured that the first infected host was in approximately the same position for all five landscape types. While the resulting plots (Fig. S1 and S2) showed that there was sufficient contrast between the host landscape types, it also showed that the variation in disease prevalence increased with increasingly clustered host landscapes. This variation was anticipated, as in more clustered landscapes, if the first infected host occurs within a dense cluster interconnected with other clusters, the simulated pest spreads more rapidly and infects a larger proportion of the population. Conversely, if the first infected host appears in an area with sparse hosts, the limited connectivity hinders the pest’s spread, resulting in a lower prevalence.

### The individual based model

The IBM adapts an SCIR (Susceptible, Cryptic, Infectious, Removed) model^39–41^ rather than the SEIR (Susceptible, Exposed, Infectious, Recovered) model because trees newly inoculated with HLB bacteria can become infectious within two weeks^42^ even while the tree is asymptomatic^43^. Since there is no effective chemical treatment for HLB^44^ and control measures are applied after delimitation, we did not model the removal of infected hosts, making the IBM a SCI model. The IBM makes two key assumptions. Firstly, infected hosts only become symptomatic an average of 365 days after infection. Anecdotal observations from the field have suggested that HLB disease symptoms manifest upwards of several months to a year after infection^25,45^, while graft inoculated trees in a laboratory manifested symptoms 200 days after infection^43^. Secondly, even though the vectors of HLB have shown preference for different citrus species^46,47^, we assumed that all host individuals are the same species.

The distances between all individuals were computed and the rate of disease transmission between a pair of hosts separated by distance $\:{d}_{ij}$ was modelled with an (unnormalized) exponential dispersal kernel, $\:K\left(d;\:\alpha\:\right)=\text{exp}\left(-\frac{d}{\alpha\:}\right)$ where $\:\alpha\:$ is the scale parameter. The IBM initializes by setting the state of each individual to susceptible. A single individual is selected at random to be the origin of the epidemic and its state is updated to cryptic. The simulation then runs in continuous time and epidemiological transitions of all individuals were simulated using the direct Gillespie’s algorithm^48^.

Because data on psyllid dispersal under natural conditions were not available, we used data on the distance travelled by psyllids when flying in an artificial mill, making the conservative assumption that average dispersal distances under natural conditions would be comparable. Arakawa and Miyamoto^49^ estimated the mean distance travelled by psyllids as 346 m under controlled conditions. We then chose the scale parameter of the exponential dispersal kernel in our model, $\:\alpha\:$, such that $\:2\times\:\alpha\:=346$ m invoking the standard relationship between the mean dispersal distance in two dimensions and the exponential dispersal kernel scale parameter^50^. With a value of 173 m for $\:\alpha\:$, we then parameterized the baseline infection rate ($\:\beta\:$). In commercial trees, the prevalence of HLB can reach 50% in two years after the first infection in blocks with only young trees (0–2 years)^25,51^. Considering that citrus trees in a city would likely be a mix of old and young trees, we parameterized $\:\beta\:$ with a line search to achieve a prevalence of 50% after five years on a random host landscape. We selected twenty-five values of $\:\beta\:$ (ranging from 0.0001/yr to 0.00022/yr), obtained the median prevalence for each value from 500 iterations, plotted the results in a graph (Fig. S3) and obtained a value of 0.0001424/yr for $\:\beta\:$. An animated GIF showing the spread of the simulated pest in a random host landscape can be found in the Supplementary Material (Fig. SA1).

With the parameterized values of $\:\alpha\:$ and $\:\beta\:$, and on a random host distribution landscape, we estimated (a) the mean maximum spread distance of the pest in the first year (1057 ± 17 m, Fig. S23a), (b) the mean maximum spread distance of the pest per generation (738 ± 7 m, Fig. S23b), and (c) the mean number of generations after 5 years (25, Fig. S24). Each of these values was estimated with a minimum of 1,000 realizations of the IBM.

For the remainder of the paper, we will use the value of 1050 m as the true maximum yearly spread distance of the pest, and the value of 750 m as the true maximum spread distance of each generation. It is worth noting that the value of 1050 m falls within the range (1 percentile: 0.9 km/yr, 99 percentile: 40.12 km/yr) from a recent expert knowledge elicitation exercise for the spread rate of HLB in the EU^26^. Although 1050 m is on the lower end of the elicited range, this is because experts estimated the spread in citrus production areas with much higher host densities than in our simulation and included natural long-distance jumps of vectors which could not occur within the size of study region we consider here. Both the mean and median number of generations present after five years was twenty-five, which indicated that our simulated pest had an average of five generations per year. This is consistent with the biology of the psyllid vectors and HLB. According to Djeddour, et al.^52^, the psyllid vectors have about nine to ten generations per year, coupled with the latent period of HLB equal to about one generation of psyllid vectors^34^, this gives about five generations of infected trees per year.

## Modelling the delimiting strategies

### Description of the three main strategies

In practice, delimiting strategies usually involve drawing a circle around infected hosts with the radius determined by an estimate of the maximum annual spread distance of the pest. This estimate can be derived through various methods, such as expert elicitation or with empirical data, but its accuracy is rarely known. Therefore, in addition to examining the effect of host distribution landscapes, we also assess the impact of underestimating, matching, or overestimating spread rate – referred to here as the “inspector-estimated” (IE) value - on the performance of the delimiting strategies. We coded three different delimiting strategies, namely the In-to-Out strategy, the Adaptive strategy, and the Multi-foci strategy (Fig. 1b). These three strategies were identified in discussion with the EFSA plant pest survey methods Working Group leading to a recommendation for the Adaptive strategy in EFSA’s General Guidelines^16^. The In-to-Out and Adaptive strategies are essentially circles of varying radii drawn around the first detected infected tree. They differ in that the In-to-Out strategy always starts with surveying the immediate area around the first infected tree and then moves outward in concentric circles until no more infected trees are detected (Fig. 1b(i) and SA2). The Adaptive strategy aims to get ahead of the spread of the pest by first estimating the boundary of the epidemic and then conducting a preliminary survey in a band outside the estimated boundary (Fig. 1b(ii)). If the epidemic boundary was correctly estimated, no infected trees will be detected in the preliminary survey and the Adaptive strategy moves inwards towards the first detection and stops when a successful detection is made (Fig. 1b(ii) and SA4). If an infected tree is detected in the preliminary survey, the Adaptive strategy behaves like the In-to-Out strategy and moves outward until no more infected trees are detected (Fig. 1b(ii) and SA3). Unlike the other two strategies, the Multi-foci strategy considers multiple detections made in each survey round and draws a circle, of fixed radius length, around each new detection. The next survey band is defined as the encircled areas that do not overlap with the previous survey band (Fig. 1b(iii) and SA5). Like the In-to-Out strategy, the Multi-foci strategy stops when no new detections are made. To assess the impact of underestimating, matching or overestimating the true spread distances of the pest, the radius of each round of all three delimiting strategies will be calculated based on several IE values.

### Calculating radius length for the delimiting strategies

Because the radii of the In-to-Out and Adaptive strategies differ between survey rounds, and each new survey band represents an additional year or generation of spread, there are multiple ways to calculate the radii. For this paper we considered three different methods to calculate the varying radii of the In-to-Out and Adaptive strategies. The first method (Linear) assumes that the pest spreads according to the IE maximum annual spread distance (Table 1). However, this is likely an overestimate because the probability of the pest dispersing to the maximum distance in consecutive years is very low. The effects of an exponential spread that is compounded yearly can be better approximated by a gamma distribution to represent multiple rounds of exponentially distributed dispersal. This may also be an overestimation since it doesn’t account for environmental factors or the effects of host distribution, but it provides a usable method. The simplest approach (Gamma Year) was to parameterize the shape parameter with the IE spread duration of the pest in years and use the corresponding IE maximum yearly spread distance to parameterize the rate parameter (Table 1). However, the underlying assumption of the Gamma Year method is that the pest has only one generation per year. Therefore, in the case of a polycyclic pest like HLB, where new infective units are produced in within the same season, it would be more accurate to parameterize the shape parameter with the IE spread duration of the pest in generations and use the IE maximum generational spread distance to parameterize the rate parameter (Gamma Gen) (Table 1). Since the IE spread distances are treated as the 95th percentile of the pest’s annual/generational spread, the expression $\:\text{ln}(1-0.95)$ transforms this 95th percentile into a form that adjusts the radius calculation accordingly. With the Multi-foci strategy, the radius of every circle was kept constant and was the IE maximum yearly spread distance of the pest. With the three versions of both the Adaptive and In-to-Out strategies and the Multi-foci strategy, there were a total of seven different strategies tested.

**Table 1:** Summary of how each delimiting strategy calculates the length of the radius. Where $\:{y}_{i}$ is the inspector-estimated spread duration of the pest in years for the $\:i$^th^ survey round, $\:{g}_{i}$ is the inspector-estimated spread duration of the pest in generations for the $\:i$^th^ survey round, $\:D$ is the inspector-estimated maximum yearly spread distance of the pest, and $\:\delta\:$ is the inspector-estimated maximum spread distance of each generation.

Delimiting Strategy	How Radius Length is Calculated
In-to-Out (Gamma Year)	$\:{Radius}_{i}=gamma\left({y}_{i},\:-\frac{\text{l}\text{n}(1-0.95)}{D}\right)$
In-to-Out (Gamma Gen)	$\:{Radius}_{i}=gamma\left({g}_{i},\:-\frac{\text{l}\text{n}(1-0.95)}{\delta\:}\right)$
In-to-Out (Linear)	$\:{Radius}_{i}={y}_{i}\times\:D$
Adaptive (Gamma Year)	$\:{Radius}_{i}=gamma\left({y}_{i},\:-\frac{\text{l}\text{n}(1-0.95)}{D}\right)$
Adaptive (Gamma Gen)	$\:{Radius}_{i}=gamma\left({g}_{i},\:-\frac{\text{l}\text{n}(1-0.95)}{\delta\:}\right)$
Adaptive (Linear)	$\:{Radius}_{i}={y}_{i}\times\:D$
Multi-foci	$\:Radius=D$

### The number of trees surveyed per round

To calculate the number of trees that need to be surveyed to detect an infection in each survey band, all seven strategies determine the number of samples required within a band to achieve a certain confidence level ($\:CL$) that detection will occur if the pest is present above a defined design prevalence ($\:DP$). For example, if the pest is not detected at $\:CL=0.8$ and $\:DP=0.1$, it suggests an 80% confidence that the pest prevalence is lower than 10% of the target population. For this, we adopted the finite population equation from EFSA’s risk-based estimate of system sensitivity (RiBESS+) tool^53^. The equation utilizes a hypergeometric probability distribution and is based on the principles originally developed by Cannon^54^. It is expressed as:

1 $$\:Sample\:size=\frac{\left(1-\:{(1-CL)}^{\frac{1}{N\times\:DP}}\right)\times\:\left(N-0.5\left(N\times\:DP\times\:MetSens-1\right)\right)}{MetSens}$$

where $\:N$ is the total number of trees within the survey band and $\:MetSens$ is the sensitivity of the detection method. In practice, both the $\:CL$ and $\:DP$ are variables determined by risk managers, balancing the trade-off between acceptability risk levels and available resources. For our tests, we used a confidence level of $\:CL=0.95$ and a design prevalence of $\:DP=0.01$; the number of trees depended on the area being surveyed, and we varied the sensitivity of detection with $\:MetSens=0.2,\:0.5,\:0.8$ and $\:1.0$. Finally, our tests of all seven delimiting strategies assume that each survey round, regardless of the number of trees or area that needs to be covered, is completed instantaneously on the 30th day after the conclusion of the previous survey round or the start of the delimiting strategy.

Assessing the performance of the delimiting strategies.

Performance was measured with four metrics.

Capability.

- The number of infected trees delimited by the strategies divided by the total number of infected trees present at the end of the delimiting survey. We also explored the proportion of simulations where a Capability of 1 was achieved (i.e. 100% of infected trees were delimited).

Efficiency.

- The area of the delimited potential infested zone divided by the area of the convex hull (the area of the smallest convex polygon needed to delimit all the infected trees present in the population at the end of the delimiting strategy).

Effort.

- The total number of trees surveyed.

Survey rounds.

- The number of survey rounds taken to delimit a potential infested zone.

We assessed the performance of all seven strategies with five different scenarios of increasing complexity. The first scenario assumed a hypothetical situation where the IE duration of pest spread and distances accurately matched the true values, the epidemic’s origin was correctly identified and used as the starting point for all seven delimiting strategies, and there was no asymptomatic period for the pest. The only variable was the sensitivity of the detection method (Method Sensitivity), tested at four levels (0.2, 0.5, 0.8, and 1.0). The second scenario was similar to the first, except that a 1-year asymptomatic period was included.

The third scenario expanded on the second by starting the delimiting strategies at a random symptomatic tree in the population, rather than the epidemic’s origin. The fourth scenario further built on the third, simulating situations where the IE annual/generational spread distance of the pest was: (1) greatly underestimated (annual: 450 m/yr; generational: 350 m/gen), (2) underestimated (annual: 750 m/yr; generational: 550 m/gen), (3) matched with the true value (annual: 1050 m/yr; generational: 750 m/gen), and (4) overestimated (annual: 1350 m/yr; generational: 950 m/gen). In this scenario, the strategies assumed a 3-year spread duration (15 generations) but started surveying when the pest had spread for only 2 years (10 generations) to overestimate the duration, or for 4 years (20 generations) to underestimate it.

The fifth scenario was like the fourth but assessed the performance of the delimiting strategies in clustered and extreme clustered landscapes generated by both the Poisson cluster process and Voronoi diagram methods.

The IBM, host distribution methods and delimiting strategies were all modelled and run in R 4.3.3^34^ and the code and parameters can be found in the Supplementary Materials (https://github.com/roguedaemon87/Supplementary-Material). To differentiate animated figures from static ones, animated figures in the Supplementary Material are labeled with the prefix SA*_*, while static figures are labeled with S*_*.

## Results

In the following sections we first explore the influence of changing the different surveillance parameters on the performance of each of the three delimiting strategies, using a ‘random host landscape’ to demonstrate this. Secondly, we explore the influence of different types of clustered landscapes on the performance of the three delimiting strategies.

### The influence of surveillance parameters on delimiting strategy performance on a random host landscape

**Fig. 2:**
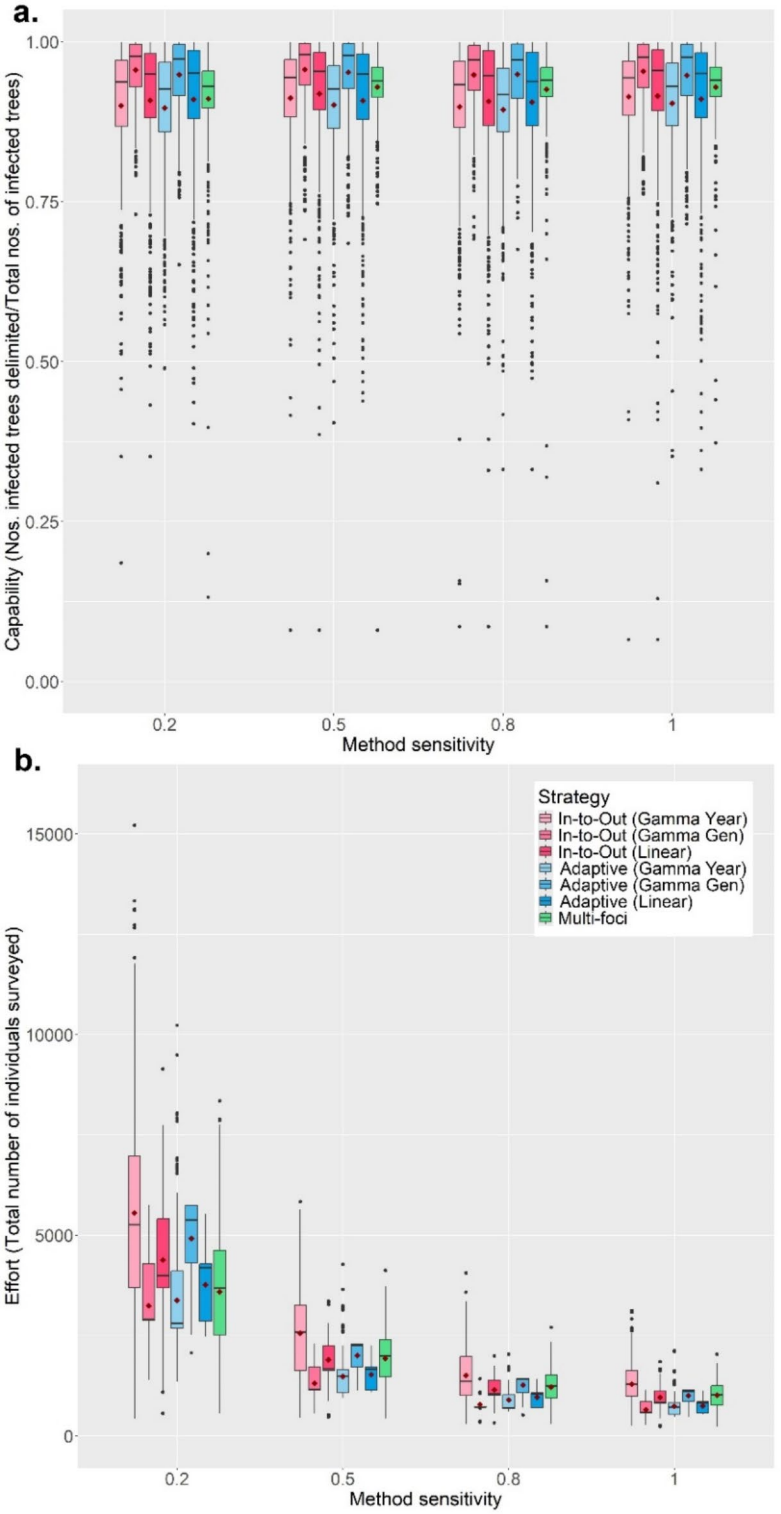
The change in Capability (**a**) and Effort (**b**) with Method sensitivity of all seven delimiting strategies on a randomly distributed host landscape. The inspector-estimated (IE) duration of the pest spread, and annual/generational spread distances were matched with the true values. For each realization of the simulated epidemic, all delimiting strategies started from the same randomly selected symptomatic individual. Each boxplot was obtained from 500 iterations. Mean values are indicated with a dark-red diamond.

**Fig. 3:**
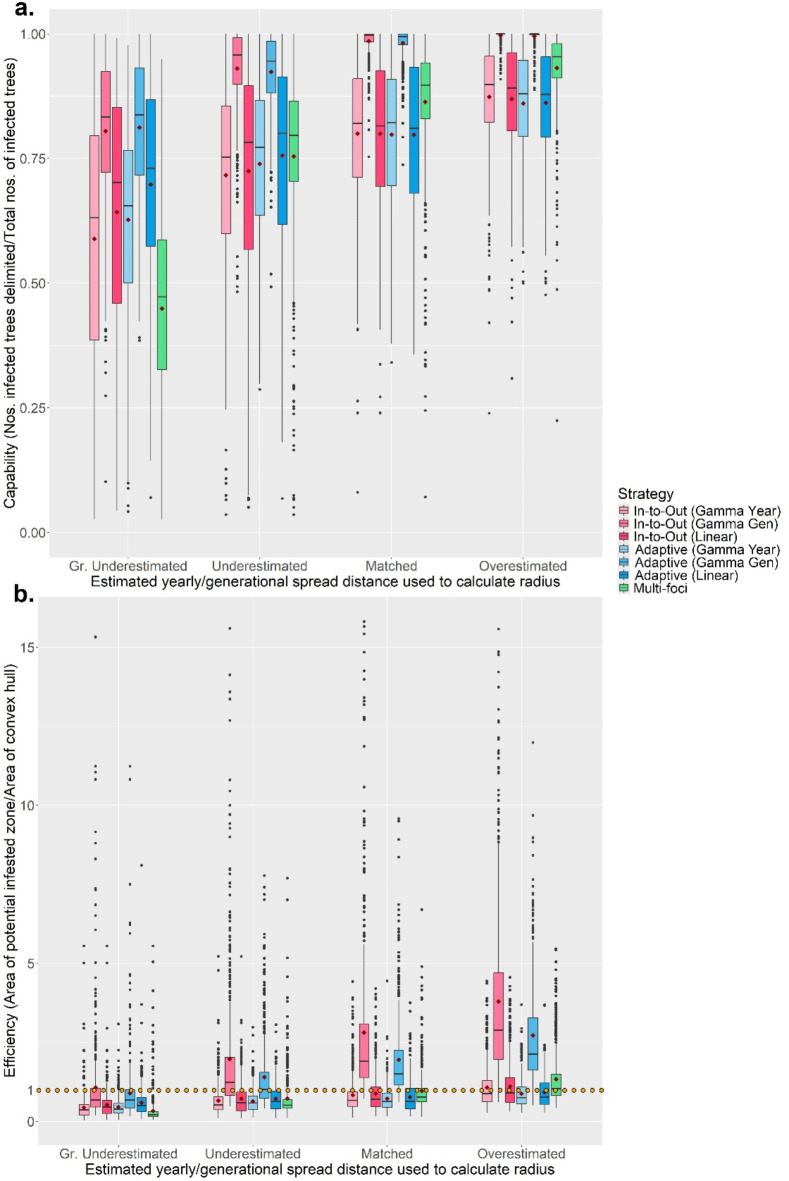
The change in Capability (**a**) and Efficiency (**b**) scores of all seven delimiting strategies on a randomly distributed host landscape with varying inspector-estimated (IE) annual/generational spread distances. The IE duration of the pest spread was overestimated, and the Method Sensitivity was 0.5. For each realization of the simulated epidemic, all delimiting strategies started from the same randomly selected symptomatic individual. Each boxplot was obtained from 500 iterations and the values of the IE spread distances are given in the table above the graph. Mean values are indicated with a dark-red diamond.

The RiBESS + equation performed well and negated the effects of changing the method sensitivity on the Capability and Efficiency of all seven strategies (i.e. as lower method sensitives were applied, this was compensated for by RiBESS + increasing sample size) (Fig. 2a). However, decreasing the method sensitivity resulted in increased Effort levels to delimit the PIZ (Fig. 2b). Varying the inspector-estimated (IE) yearly/generational spread distance profoundly affected the performance of all seven strategies (Fig. 3). Capability of all seven strategies was poor when the IE spread distances were lower than the true values and improved when the IE spread distance was equal to or greater than the true values (Fig. 3a). Throughout the first four scenarios, the Gamma Gen (both In-to-Out and Adaptive) strategies had the highest Capability levels, albeit at higher Effort levels and at lower Efficiency (when the IE spread distance was matched or overestimated) than the other strategies.

**Fig. 4:**
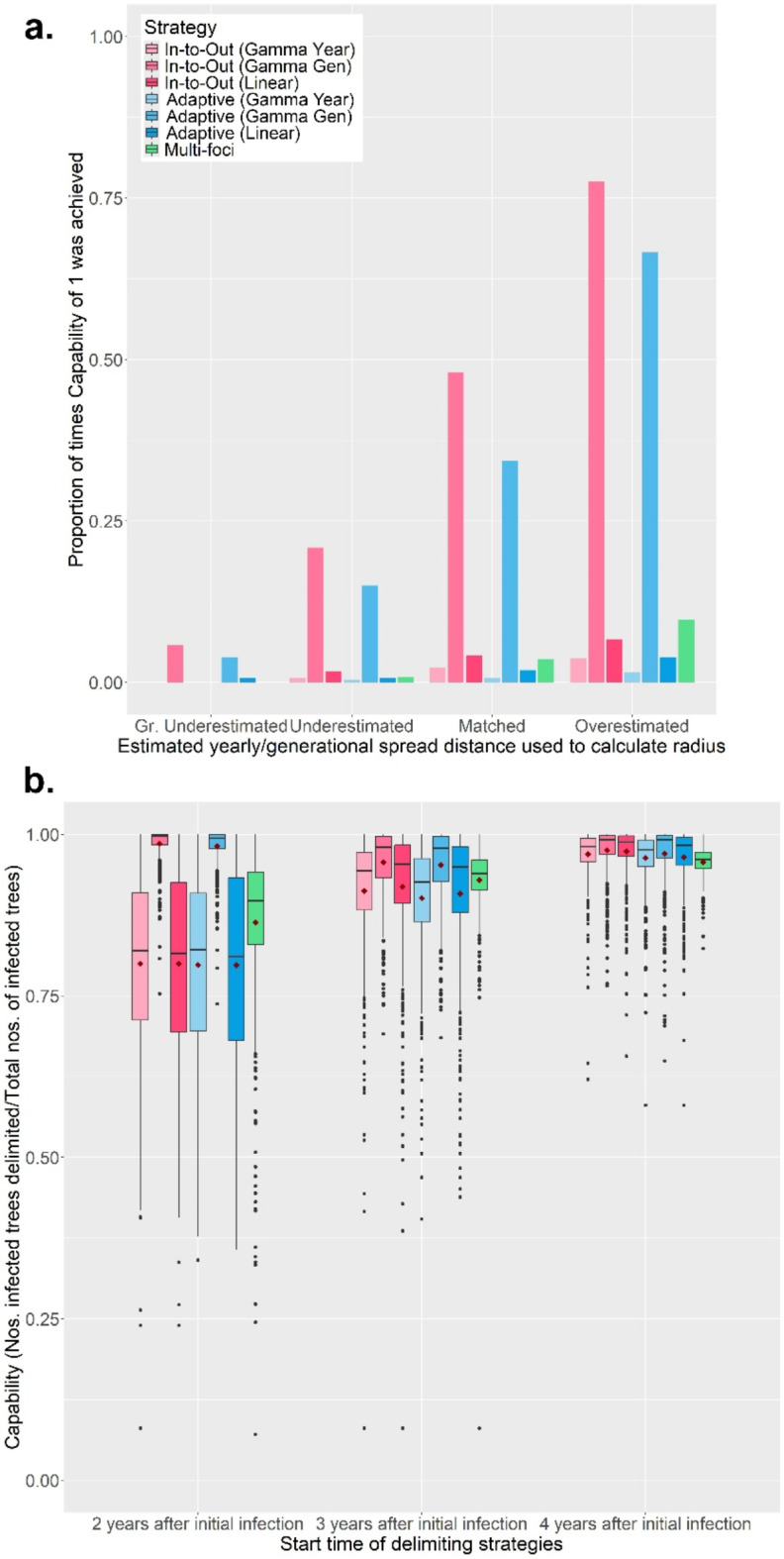
(**a**) The change of how often each of the seven delimiting strategies achieved a perfect Capability score of 1 with inspector-estimated (IE) annual/generational spread distances on a randomly distributed host landscape. The IE duration of pest spread was overestimated. (**b**) The change in Capability scores of all seven delimiting strategies on a randomly distributed host landscape with varying start times of the delimiting strategies. The inspector-estimated (IE) duration of the pest spread was 3-years, the IE spread distance was matched with the true value (annual: 1050 m/yr; generational: 750 m/yr). For both panels, in each realization of the simulated epidemic, all delimiting strategies started from the same randomly selected symptomatic individual. Each boxplot and bar were obtained from 500 iterations and mean values for the boxplots are indicated with a dark-red diamond. Method sensitivity was 0.5.

**Fig. 5:**
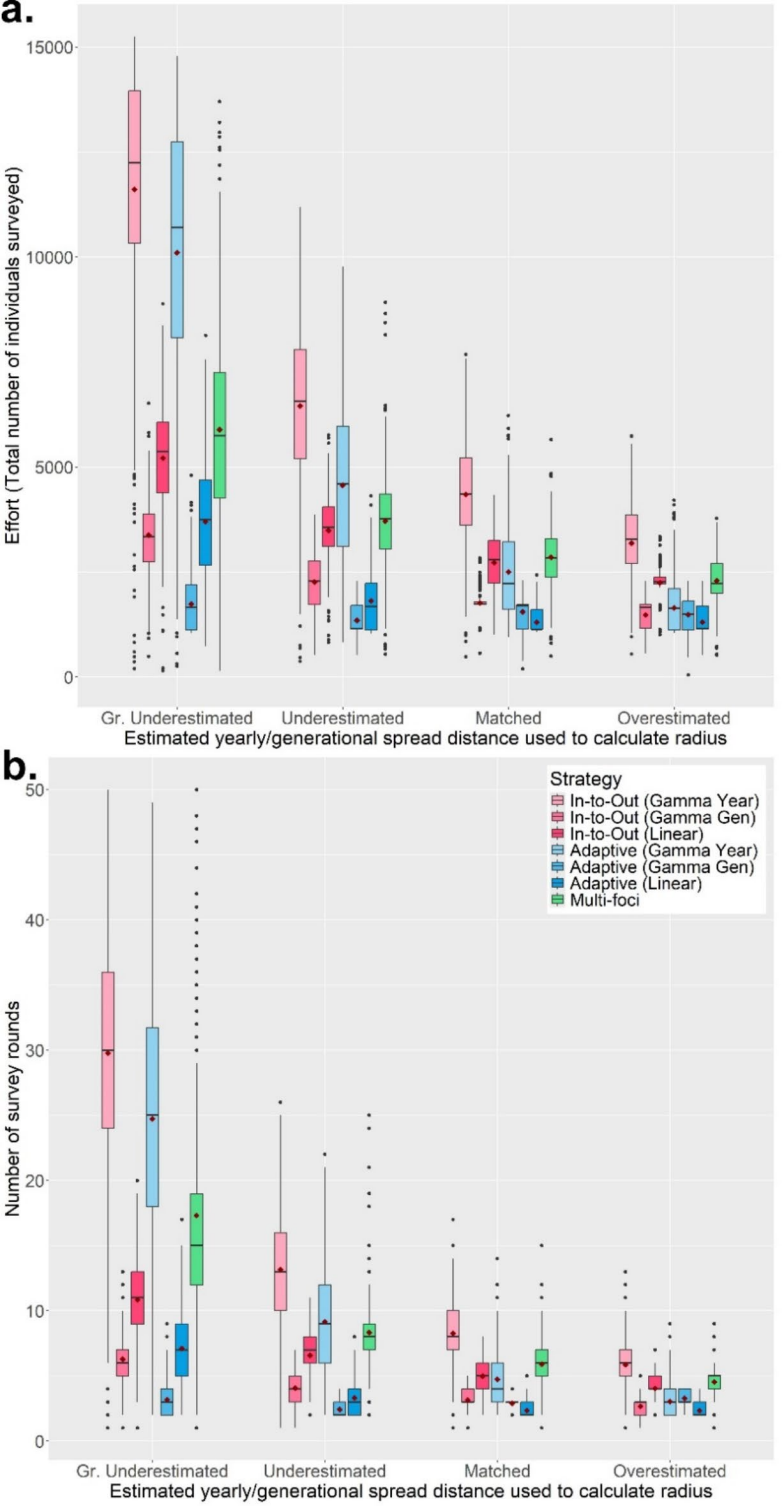
The change in Effort (**a**) and the number of survey rounds (**b**) of all seven delimiting strategies on a randomly distributed host landscape with varying inspector-estimated (IE) annual/generational spread distances. The IE duration of the pest spread was overestimated, and the Method Sensitivity was 0.5. For each realization of the simulated epidemic, all delimiting strategies started from the same randomly selected symptomatic individual. Each boxplot was obtained from 500 iterations and the values of the IE spread distances are given in the table above the graph. Mean values are indicated with a dark-red diamond.

The Gamma Gen (both In-to-Out and Adaptive) strategies also achieved perfect Capability scores the most often, even across different host landscapes (Fig. 4a and S19), and performed well regardless of whether the IE mean number of generations per year was underestimated or overestimated (Fig. S11 to S14). However, in most scenarios, the Adaptive (Gamma Gen) strategy had a higher Efficiency (Method Sensitivity = 0.2, 0.5, 0.8, 1.0; median: 0.67, 1.00, 1.51, 2.13) than the In-to-Out (Gamma Gen) strategy (Method Sensitivity = 0.2, 0.5, 0.8, 1.0; median: 0.67, 1.23, 1.89, 2.88) (Fig. 3b), needed less Effort (Fig. 5a) and fewer survey rounds (Fig. 5b) than the In-to-Out (Gamma Gen) strategy and therefore was the overall best strategy. Changing the IE duration of the pest spread also affected the performance of all seven strategies. The Gamma Gen strategies performed well when the pest spread duration was overestimated (delimiting strategies initiated 2 years post-infection) but declined in Capability when the duration was accurately matched or underestimated (delimiting strategies initiated 4 years post-infection) (Fig. 4b). In contrast, the Gamma Year, Linear, and Multi-foci strategies showed improved performance with longer pest spread durations (Fig. 4b).

### The influence of clustered host landscapes on delimiting strategy performance

**Fig. 6:**
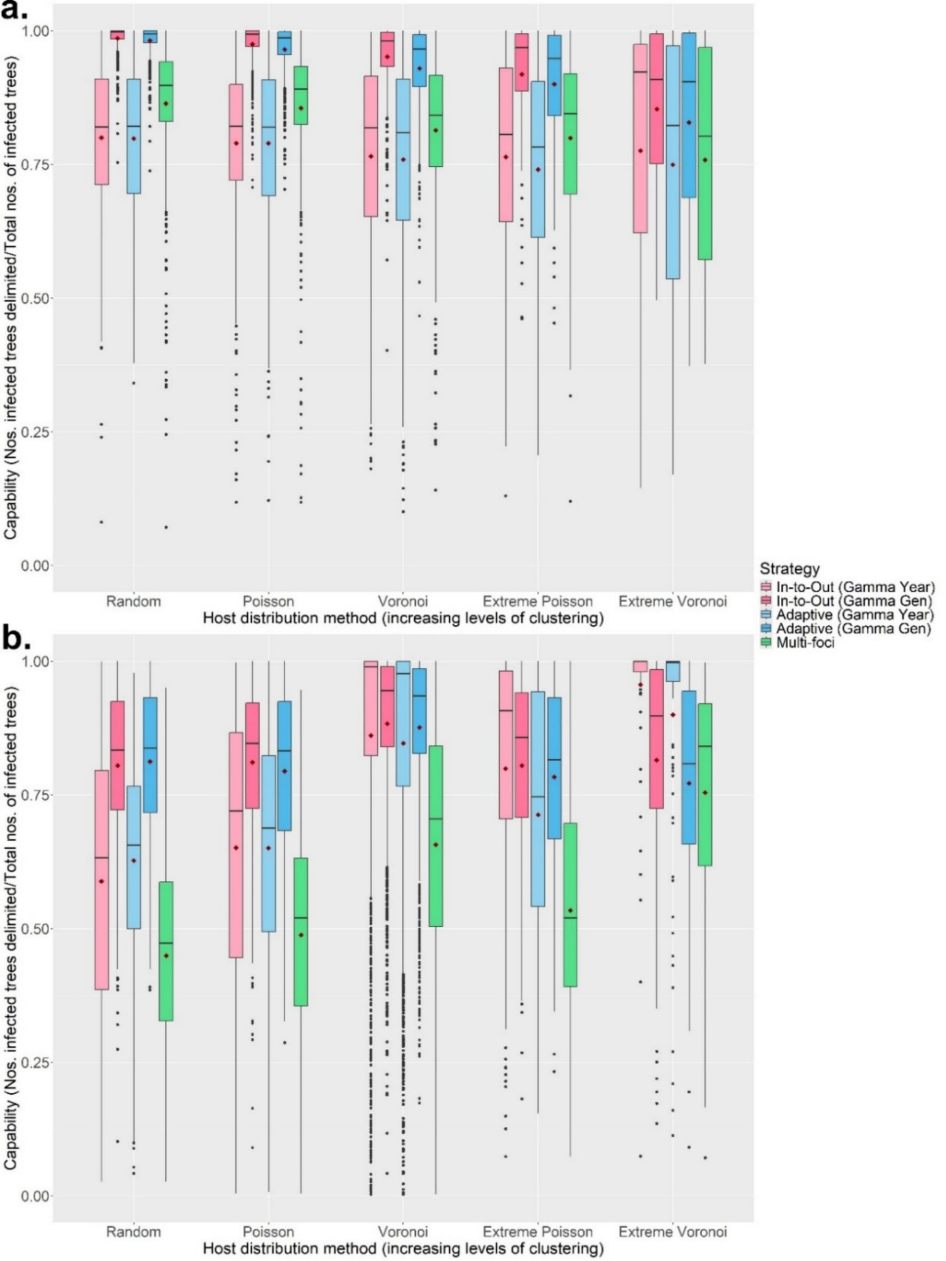
The change in the Capability scores of the delimiting strategies on various host landscape types and when the Method Sensitivity was 0.5, and the IE duration of the pest spread was overestimated. The inspector-estimated (IE) spread distances were matched with the true value (**a**) and greatly underestimated (**b**) (450 m/yr and 350 m/gen). For each realization of the simulated epidemic, all delimiting strategies started from the same randomly selected symptomatic individual. Boxplots for the Random, Poisson and Voronoi landscapes were obtained from 500 iterations, while boxplots for the Extreme Poisson and Extreme Voronoi landscapes were obtained from 200 iterations. Mean values are indicated with a dark-red diamond. The performance of the In-to-Out (Linear) and Adaptive (Linear) strategies was not assessed on clustered host landscapes.

**Fig. 7:**
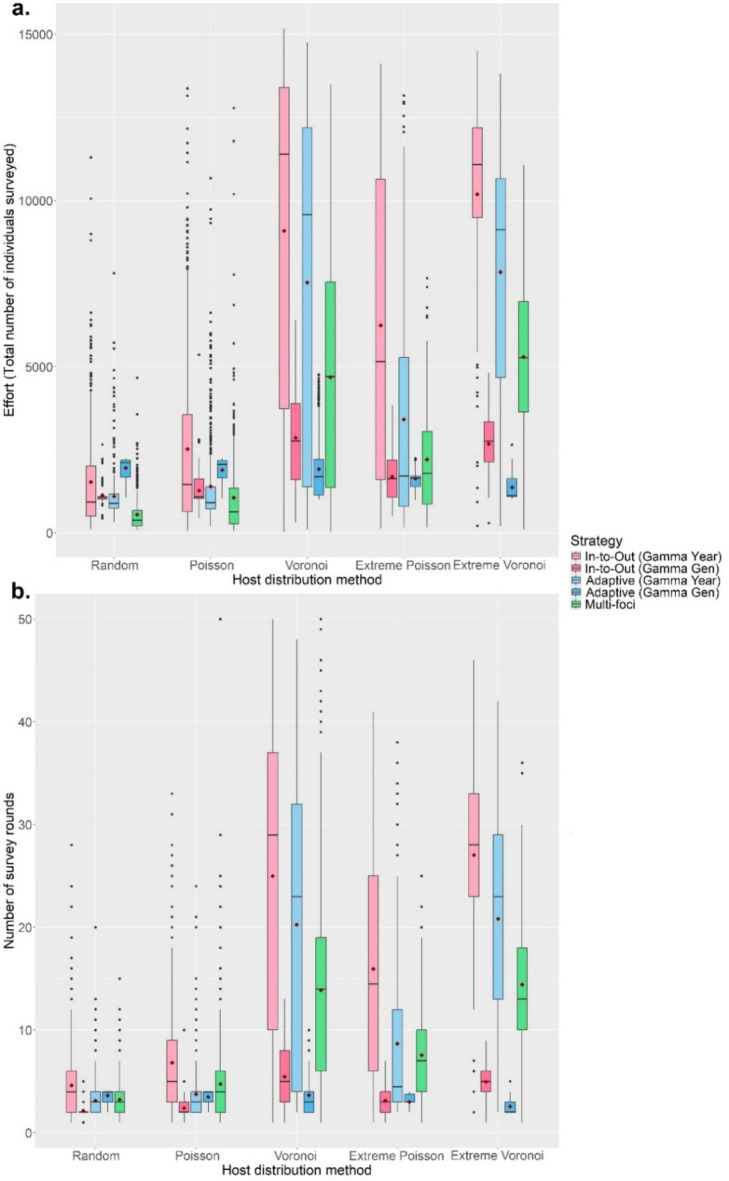
The change in Effort scores (**a**) and number of survey rounds (**b**) of each delimiting strategy on various landscape types. The Method Sensitivity was 0.5, the inspector-estimated (IE) spread distances were greatly underestimated (450 m/year and 350 m/generation) and the IE duration of the pest spread was overestimated. For each realization of the simulated epidemic, all delimiting strategies started from the same randomly selected symptomatic individual. Boxplots for the random host, Poisson and Voronoi landscapes were obtained from 500 iterations, while boxplots for the Extreme Poisson and Extreme Voronoi landscapes were obtained from 200 iterations. Mean values are indicated with a dark-red diamond.

Although increasing the level of clustering resulted in greater variation in performance, the Gamma Gen strategies maintained the highest Capability scores (Fig. 6a). While the In-to-Out (Gamma Gen) strategy slightly outperformed the Adaptive (Gamma Gen) strategy in Capability, the Adaptive (Gamma Gen) strategy proved more Efficient in most scenarios, requiring fewer survey rounds and less Effort than the In-to-Out (Gamma Gen) strategy. However, when the IE spread distances were lower than the true value, the Capability scores of the Gamma Year and Multi-foci strategies improved with increasing levels of clustering and host heterogeneity, with the former surpassing the Capability scores of the Gamma Gen strategies in Extreme Voronoi Diagram host landscapes (Fig. 6b). This was because the Gamma Year strategies’ shorter radii better matched the spread of symptomatic individuals, which had been slowed and bottlenecked by the heterogenous landscape (Fig. S21, SA6 and SA7). However, this in turn resulted in the Gamma Year strategies needing much more Effort (Fig. 7a) and many more survey rounds (Fig. 7b) than the Gamma Gen strategies to delimit the PIZ. The full plots of each scenario can be found in the Supplementary Materials (Scenario 1: Fig. S4; Scenario 2: Fig. S5; Scenario 3: Fig. S6; Scenario 4: Fig. S7–10; Scenario 5: Fig. S15 – S18).

## Discussion

Delimiting strategies are essential for effective outbreak management, whether for eradication or containment. Without science-based approaches, delimited areas may be inadequately sized—too small, allowing pests to spread unchecked, or too large, making eradication economically unfeasible. In this study, we focused on the economically significant causal agent of citrus HLB to illustrate how the design of delimiting strategies depends on the pest’s epidemiology. We compared various delimiting strategies across different scenarios and host landscape types to objectively determine the best-performing strategy. On a random host landscape, the Multi-foci strategy had the worst performance. Its performance was most negatively affected when the strategy started on a random symptomatic tree and asymptomatic trees cannot be detected. This was mainly caused by two reasons. Firstly, because the IBM models the spread with an exponential dispersal kernel, the epidemic spreads from its origin and creates two fronts: an inner front of symptomatic hosts and an outer front of asymptomatic hosts. Hence, when the Multi-foci strategy begins at a random symptomatic tree on the edge of the symptomatic front, it encounters more symptomatic hosts towards the epidemic’s origin and fewer in other directions. This bias persists even with perfect method sensitivity, as fewer hosts are sampled away from the origin. At lower sensitivities, imperfect detection further reinforces this trend, leading the strategy to focus inward rather than outward. Secondly, the constant and relatively short radii of the Multi-foci strategy results in survey circles that fail to capture the full extent of the spread, especially given the presence of asymptomatic trees. An example of these reasons is provided in Fig. SA8 of the Supplementary Material. For comparison, Fig. SA9 illustrates the performance of the Adaptive (Gamma Gen) strategy in the same scenario as Fig. SA8. The In-to-Out and Adaptive strategies performed similarly in terms of Capability, but the Adaptive strategy was generally more Efficient and required fewer survey rounds and less Effort in most situations. However, the In-to-Out strategy outperformed the Adaptive strategy in terms of Capability, Effort, and Number of Survey Rounds when the pest was detected early (two years after the initial infection) (Fig. S7, S9 and S10). The strong performance of the In-to-Out strategy when the pest was detected early aligns with the findings of other research studies^55,56^ and can be attributed to the strategy’s initial focus on surveying the immediate area around the starting point while the epidemic was still relatively small. This allowed the In-to-Out strategy to delimit the PIZ faster and more efficiently than the Adaptive strategy. Therefore, if the target pest’s dispersal rate is low or restrained and the confidence of spread duration is high, the In-to-Out strategy would be preferable over the Adaptive strategy. However, in practice, the timing of pest detection and identification is often unclear in relation to when it was first introduced to an area. This makes the Adaptive strategy the safer and more conservative option.

Of the three different versions of the Adaptive strategy, the Gamma Gen version performed the best. Compared to the Linear and Gamma Year versions, it consistently had the highest Capability levels (Fig. 3a) and even had an average Capability of > 90% when the generational spread distance was underestimated (Fig. S7). The Gamma Gen version also benefitted from earlier pest detections, as its wider survey bands were able to encompass most infected hosts (Fig. 4b). Although the relative Capability of its wider bands decreased as pest spread duration increased, it still outperformed the Gamma Year and Linear versions in terms of Capability (Fig. 4b). Additional details regarding the effect of the duration of pest spread on the delimiting strategies can be found in the Supplementary Materials. The Gamma Gen version was less efficient than the other versions due to its wider survey bands, which tended to overestimate the area of the convex hull. However, even when the generational spread distance was overestimated, it only delimited an area approximately 2 times the convex hull on average (Fig. S12). The superior performance of the Gamma Gen version stems from its suitability in tracking a polycyclic pest like HLB. Unlike the Linear and Gamma Year versions, which assume the pest has only one generation – and thus one spread event – per year, the Gamma Gen version accounts for multiple spread events annually. This was further confirmed when we analyzed how frequently each version accurately estimated the epidemic’s spread distance and moved inward (Fig. S20), as well as by comparing the probability density function values derived from the gamma distribution with a histogram of the pest’s spread distances after five years (Fig. S21 and S22). Of the three versions, the Gamma Gen version was the only one that moved inwards > 90% of the time when the estimated spread distance was matched, regardless of whether the IE duration of pest spread was overestimated, matched or underestimated. Even when the IE number of generations per year was underestimated to three generations per year and the IE generational spread distance was greatly underestimated, if the IE duration of the pest spread was matched or underestimated, the Adaptive (Gamma Gen) strategy could achieve Capability levels of > 90% (Fig. S11). Therefore, the Adaptive (Gamma Gen) strategy emerged as the overall best strategy in a random host landscape.

The Adaptive (Gamma Gen) strategy remained the best option, even on clustered landscapes where all delimiting strategies showed greater variation in performance due to host distribution heterogeneity. While the In-to-Out (Gamma Year) and Adaptive (Gamma Year) strategies achieved higher levels of Capability when the IE spread distances were underestimated, the Adaptive (Gamma Gen) was more efficient and required less Effort to delimit the PIZ. It is important to note that while the difference in Capability between the Adaptive (Gamma Year) and the Adaptive (Gamma Gen) strategies averaged 0.19 in an unrealistically clustered urban landscape (i.e. Extreme Voronoi), when the IE spread distances were underestimated, the difference in Capability was closer to 0.05 in more realistic scenarios (i.e. Voronoi column in Fig. 6). The lower Capability level of the Adaptive (Gamma Gen) strategy on highly clustered landscapes was due to its wider survey bands overestimating the spread of symptomatic individuals. This issue arose only in clustered landscapes, where individuals were densely packed in patches, and the low density of individuals between patches slowed the spread of symptomatic individuals. This finding aligns with metapopulation theory and has been documented in plant disease incidence in forests and grasslands^57–59^. These results provide actionable insights for the design of pest management programs, particularly in regions where resources for monitoring and containment are limited. The ability of the Adaptive (Gamma Gen) strategy to maintain high Capability across different landscape types offers flexibility in its implementation, which could be especially valuable in regions facing logistical or financial constraints. While the performance of the delimiting strategies was not evaluated using actual real-world distribution data of urban citrus trees, simulated host distributions generated through the Voronoi diagram method, based on satellite images of Seville, offer a close approximation. Using Earth observation data, which in principle allows for the mapping of individual trees, could provide a valuable methodology to improve this approximation. However, this approach is far from trivial due to challenges such as noise from buildings and other nearby objects in urban areas, as well as the difficulty of accurately identifying citrus trees among other intermixed species^60^.

Thus far this study has shown the importance of tailoring a delimiting strategy to the characteristics of the target pest. Considering the long asymptomatic period and polycyclic nature of HLB, our case study pest, vastly improved the performance of the Adaptive strategy. This also implies that if a different pest was simulated, for example a monocyclic pest like *Crinipellis perniciosa*^61^ or a polycyclic pest with a longer asymptomatic period like *X. fastidiosa*^62^, the other strategies may outperform the Adaptive (Gamma Gen) strategy. This underscores the importance of evaluating different delimiting strategies for their suitability in targeting specific pests, making the methodology presented in this paper valuable for future studies. Besides re-parameterizing the IBM with different dispersal kernels (e.g. inverse-power law kernel^63^ and exponential-power kernel^50^), baseline infection rates and scale parameters to simulate different pests, the delimiting strategy models can be included in large scale spread simulations, like Ellis, et al.^64^, Radici, et al.^65^ and Nguyen, et al.^66^, to develop effective coordinated management strategies and contingency plans. Future studies could also explore how these delimiting strategies perform under varying environmental stressors such as climate change or dispersal barriers, which can alter pest dispersal patterns. Additionally incorporating dynamic factors like changing host availability or explicitly modelling a highly heterogeneous vector population into future iterations of the IBM could yield more precise strategy recommendations. Exploring mixed host landscapes—such as a clustered host population partially generated using the Poisson cluster process and partially using the Voronoi diagram method—would be an intriguing avenue for future studies and is already possible using our code.

In addition to the assumptions mentioned in the Methodology section, the IBM did not simulate the movement or distribution of a vector population. Instead, we modelled the psyllid vectors implicitly with the baseline infection rate and assumed a uniformly distributed vector population across the landscape, with constant dispersal throughout the year. In reality, local dispersal of HLB vectors tend to be greatest during the warmer months and decreases greatly during the autumn and winter and winter months^21^. Vector population densities in the field are also much higher in areas with high densities of seedling trees than in areas mostly populated by mature trees^67^. We chose to model the vectors implicitly to ensure that the framework is generic, allowing the IBM to be adapted for other pests. This approach aligns with previous studies^40,68–70^, which have shown that models without explicitly modeling the vectors can still be effective for HLB. Like the IBM, the delimiting strategy models are not fully realistic. For example, the strategies do not account for false positive or false negative detections; the latter is especially likely for HLB as the HLB bacterium and pathogenic bacteria are unevenly distributed in plant tissues and sampling the wrong spot in a symptomatic individual can result in false negatives in polymerase chain reaction (PCR) tests^71^. However, since false negatives occur approximately 20% and 16% of the time for conventional PCR methods^72^ and real-time PCR methods^73^ respectively, their effect is likely nullified by the number of samples taken in each survey round in our simulations.

## Conclusion

The impact of invading plant pests can be extremely severe, and so optimizing control, monitoring and reaction strategies is imperative. Although much research has focused on improving surveillance methods, enhancing the accuracy and speed of pest detection, and increasing the efficiency of various control measures, little attention has been given to evaluating strategies for delimiting the extend of pest spread. Despite its limitations, this study highlights the importance of evaluating the suitability of delimiting strategies and adapting them to the specific characteristics of the targeted pest. In the case of polycyclic pests with a long asymptomatic period like HLB, the Adaptive (Gamma Gen) strategy outperformed the other strategies discussed in this paper. Additionally, this study presents a framework for assessing the performance of three delimiting strategies, with potential for future implementation in larger-scale research projects to enhance pest management strategies.

## Electronic supplementary material

Below is the link to the electronic supplementary material.

Supplementary Material 1

Supplementary Material 2

Supplementary Material 3

Supplementary Material 4

Supplementary Material 5

Supplementary Material 6

Supplementary Material 7

Supplementary Material 8

Supplementary Material 9

Supplementary Material 10

Supplementary Material 11

Supplementary Material 12

Supplementary Material 13

Supplementary Material 14

Supplementary Material 15

Supplementary Material 16

Supplementary Material 17

Supplementary Material 18

Supplementary Material 19

Supplementary Material 20

Supplementary Material 21

Supplementary Material 22

Supplementary Material 23

Supplementary Material 24

Supplementary Material 25

Supplementary Material 26

Supplementary Material 27

Supplementary Material 28

Supplementary Material 29

Supplementary Material 30

Supplementary Material 31

Supplementary Material 32

Supplementary Material 33

Supplementary Material 34

Supplementary Material 35

## Data Availability

The data supporting the findings of this study, as well as those generated or analyzed during the research, are available from the corresponding author upon request.

## References

[1] Ristaino, J. B. et al. The persistent threat of emerging plant disease pandemics to global food security. *Proceedings of the National Academy of Sciences* 118, e2022239118 (2021). 10.1073/pnas.2022239118 10.1073/pnas.2022239118 PMC8201941 34021073

[2] Panetta, F. D. & Lawes, R. Evaluation of weed eradication programs: the delimitation of extent. *Divers. Distrib.* **11**, 435–442 (2005).

[3] Spence, N., Hill, L. & Morris, J. How the global threat of pests and diseases impacts plants, people, and the planet. *PLANTS PEOPLE PLANET.* **2**, 5–13. 10.1002/ppp3.10088 (2020).

[4] Ali, B. M., van der Werf, W. & Oude Lansink, A. Assessment of the environmental impacts of *Xylella fastidiosa* subsp. *Pauca* in Puglia. *Crop Prot.* **142**, 105519. 10.1016/j.cropro.2020.105519 (2021).

[5] Schneider, K. et al. Impact of Xylella fastidiosa subspecies pauca in European olives. *Proc. Natl. Acad. Sci.* **117**, 9250–9259 (2020). 32284411 10.1073/pnas.1912206117 PMC7196823

[6] IPPC Secretariat. *Glossary of Phytosanitary Terms. International Standard for Phytosanitary Measures No. 5* (Food and Agriculture Organization of the United Nations on behalf of the Secretariat of the International Plant Protection Convention, 2023).

[7] European Parliament of the Council. Regulation (EU) 2016/2031 on protective measures against pests of plants. (2016).

[8] Stanaway, M. A. *Hierarchical Bayesian Models for Estimating the Extent of Plant pest Invasions* (Queensland University of Technology, 2011).

[9] Nardi, S. et al. Control of *Rhynchophorus Ferrugineus* (Olivier, 1790) according to EU decision 2007/365/EC in the Marche region (Central-Eastern Italy). *EPPO Bull.* **41**, 103–115 (2011).

[10] Lázaro, E. et al. Tracking the outbreak: an optimized sequential adaptive strategy for *Xylella fastidiosa* delimiting surveys. *Biol. Invasions*. **23**, 3243–3261 (2021).

[11] Dominic, E. & Neil, P. (eds) *(Ed Food & Rural Affairs Department for Environment)* (DEFRA, York, 2022).

[12] Miller, M. et al. *Control and Eradication*. in *Disease Control Priorities in Developing Countries* (eds D. T. Jamison Oxford University Press, Ch. 62, (2006).

[13] Carrasco, L. R. et al. Dispersal kernels of the invasive alien western corn rootworm and the effectiveness of buffer zones in eradication programmes in Europe. *Ann. Appl. Biol.* **156**, 63–77. 10.1111/j.1744-7348.2009.00363.x (2010).

[14] White, S. M., Bullock, J. M., Hooftman, D. A. P. & Chapman, D. S. Modelling the spread and control of Xylella fastidiosa in the early stages of invasion in Apulia, Italy. *Biol. Invasions*. **19**, 1825–1837. 10.1007/s10530-017-1393-5 (2017). 32025190 10.1007/s10530-017-1393-5 PMC6979717

[15] Rimbaud, L. et al. Improving Management Strategies of Plant Diseases Using Sequential Sensitivity Analyses. *Phytopathology*^®^ 109, 1184–1197 (2019). 10.1094/phyto-06-18-0196-r 10.1094/PHYTO-06-18-0196-R 30844325

[16] EFSA. General guidelines for statistically sound and risk-based surveys of plant pests. *EFSA Supporting Publications*. **17**, 1919E (2020).

[17] EPPO. PM 5/10 (1) Guidelines on the design and implementation of a buffer zone. EPPO Bulletin 51, 438–450. (2021). 10.1111/epp.12777

[18] Gottwald, T. R., Graham, J. H. & Schubert, T. S. Citrus canker: the Pathogen and its impact. *Plant. Health Progress*. **3**, 15. 10.1094/php-2002-0812-01-rv (2002).

[19] Paap, T., Burgess, T. I. & Wingfield, M. J. Urban trees: bridge-heads for forest pest invasions and sentinels for early detection. *Biol. Invasions*. **19**, 3515–3526. 10.1007/s10530-017-1595-x (2017).

[20] Sicard, A. et al. *Xylella fastidiosa*: Insights inemergingerging Plant Pathogen. *Annu. Rev. Phytopathol.* **56**, 181–202. 10.1146/annurev-phyto-080417-045849 (2018). https://doi.org/https://doi.org/29889627 10.1146/annurev-phyto-080417-045849

[21] Stelinski, L. L. Ecological aspects of the Vector-Borne Bacterial Disease, Citrus Greening (Huanglongbing): dispersal and host use by Asian Citrus psyllid, *Diaphorina Citri* Kuwayama. *Insects* **10**, 208 (2019). 31315295 10.3390/insects10070208 PMC6681385

[22] Nunes, P., Robinet, C., Branco, M. & Franco, J. C. Modelling the invasion dynamics of the African citrus psyllid: the role of human-mediated dispersal and urban and peri-urban citrus trees. *NeoBiota* **84**, 369–396 (2023).

[23] Milosavljević, I., Schall, K., Hoddle, C., Morgan, D. & Hoddle, M. Biocontrol program targets Asian citrus psyllid in California’s urban areas. *Calif. Agric.* **71**, 169–177 (2017).

[24] Garcia, A. G., Jamielniak, J. A., Diniz, A. J. F. & Parra, J. R. P. The importance of Integrated Pest Management to flatten the huanglongbing (HLB) curve and limit its vector, the Asian citrus psyllid. *Entomol. Generalis*. **42**, 349–359 (2022).

[25] Gottwald, T. R. Current epidemiological understanding of citrus huanglongbing. *Annu. Rev. Phytopathol.* **48**, 119–139 (2010). 20415578 10.1146/annurev-phyto-073009-114418

[26] EFSA. Report on the methodology applied by EFSA to provide a quantitative assessment of pest-related criteria required to rank candidate priority pests as defined by regulation (EU) 2016/2031. *EFSA J.* **17**, e05731. 10.2903/j.efsa.2019.5731 (2019). 32626353 10.2903/j.efsa.2019.5731 PMC7009284

[27] Belasque, J. Jr et al. Lessons from huanglongbing management in São Paulo state, Brazil. *J. Plant. Pathol.*, 285–302 (2010).

[28] Graham, J., Gottwald, T. & Setamou, M. Status of huanglongbing (HLB) outbreaks in Florida, California and Texas. *Trop. Plant. Pathol.* **45**, 265–278 (2020).

[29] Li, S., Wu, F., Duan, Y., Singerman, A. & Guan, Z. Citrus greening: management strategies and their economic impact. *HortScience* **55**, 604–612 (2020).

[30] Siverio, F. et al. Survey of huanglongbing associated with ‘*Candidatus* Liberibacter’ species in Spain: analyses of citrus plants and *Trioza erytreae*. *Phytopathologia Mediterranea*, 98–110 (2017).

[31] EPPO (European and Mediterranean Plant Protection Organisation). *Global Database. First Report of Diaphorina citri in Cyprus* (EPPO, 2023).

[32] Liu, Y., Heying, E. & Tanumihardjo, S. A. History, global distribution, and nutritional importance of citrus fruits. *Compr. Rev. Food Sci. Food Saf.* **11**, 530–545 (2012).

[33] Mastin, A. J., van den Bosch, F., Bourhis, Y. & Parnell, S. Epidemiologically-based strategies for the detection of emerging plant pathogens. *Sci. Rep.* **12**, 10972 (2022). 35768558 10.1038/s41598-022-13553-y PMC9243127

[34] R. A Language and Environment for Statistical Computing (R Foundation for Statistical Computing, Vienna, Austria, 2024). https://www.R-project.org/

[35] Oliva, S. R. & Bonells, J. E. *El naranjo amargo de Sevilla*, < (1996). https://www.sevilla.org/servicios/medio-ambiente-parques-jardines/e-articulos-tecnicos/naranjo_amargo.pdf

[36] Ayutamiento de Sevilla. *Mitos y realidades sobre la naranja amarga de Sevilla*, (2021). https://www.sevilla.org/actualidad/blog/mitos-y-realidades-sobre-la-naranja-amarga-de-sevilla#:~:text=Actualmente%2C%20las%20calles%20de%20la,pasada%20(2019%2D2020)&gt

[37] Galvañ, A. et al. Risk-based regionalization approach for area-wide management of HLB vectors in the Mediterranean Basin. *Front. Plant Sci.* **14**10.3389/fpls.2023.1256935 (2023). 10.3389/fpls.2023.1256935 PMC10725980 38111874

[38] Baddeley, A. & Turner, R. Spatstat: an R Package for analyzing spatial point patterns. *J. Stat. Softw.* **12**, 1–42. 10.18637/jss.v012.i06 (2005).

[39] Cunniffe, N. J. & Gilligan, C. A. *CHAPTER 12: Use of Mathematical Models to Predict Epidemics and to Optimize Disease Detection and Management*. in *Emerging Plant Diseases and Global Food Security* (eds Jean Beagle. Ristaino & Angela. Records) 239–266The American Phytopathological Society, (2020).

[40] Cunniffe, N. J., Stutt, R. O. J. H., DeSimone, R. E., Gottwald, T. R. & Gilligan, C. A. Optimising and communicating options for the Control of Invasive Plant Disease when there is epidemiological uncertainty. *PLoS Comput. Biol.* **11**, e1004211. 10.1371/journal.pcbi.1004211 (2015). 25874622 10.1371/journal.pcbi.1004211 PMC4395213

[41] Hyatt-Twynam, S. R. et al. Risk-based management of invading plant disease. *New Phytol.* **214**, 1317–1329. 10.1111/nph.14488 (2017). 28370154 10.1111/nph.14488 PMC5413851

[42] Lee, J. A. et al. Asymptomatic spread of huanglongbing and implications for disease control. *Proc. Natl. Acad. Sci.* **112**, 7605–7610 (2015). 26034273 10.1073/pnas.1508253112 PMC4475945

[43] Coletta-Filho, H. D., Daugherty, M. P., Ferreira, C. & Lopes, J. R. S. Temporal progression of ‘Candidatus Liberibacter asiaticus’ infection in Citrus and Acquisition Efficiency by Diaphorina citri. *Phytopathology*^®^**104**, 416–421. 10.1094/phyto-06-13-0157-r (2014). 24620723 10.1094/PHYTO-06-13-0157-R

[44] Munir, S. et al. The hidden treasures of citrus: finding Huanglongbing cure where it was lost. *Crit. Rev. Biotechnol.* **42**, 634–649. 10.1080/07388551.2021.1942780 (2022). 34325576 10.1080/07388551.2021.1942780

[45] Manjunath, K. L., Halbert, S. E., Ramadugu, C., Webb, S. & Lee, R. F. Detection of ‘*Candidatus* Liberibacter *asiaticus*’ in *Diaphorina citri* and its importance in the management of Citrus Huanglongbing. *Fla. Phytopathology*^®^. **98**, 387–396. 10.1094/phyto-98-4-0387 (2008). 10.1094/PHYTO-98-4-0387 18944186

[46] Gasparoto, M., Hau, B., Bassanezi, R., Rodrigues, J. & Amorim, L. Spatiotemporal dynamics of citrus huanglongbing spread: a case study. *Plant. Pathol.* **67**, 1621–1628 (2018).

[47] Benhadi-Marín, J., Garzo, E., Moreno, A., Pereira, J. A. & Fereres, A. Host plant preference of Trioza erytreae on lemon and bitter orange plants. *Arthropod-Plant Interact.* **15**, 887–896. 10.1007/s11829-021-09862-0 (2021).

[48] Keeling, M. J. & Rohani, P. *Modeling Infectious Diseases in Humans and Animals* (Princeton University Press, 2008).

[49] Arakawa, K. & Miyamoto, K. Flight ability of Asiatic citrus psyllid, *Diaphorina citri* Kuwayama (Homoptera; Psyllidae), measured by a flight mill. *Res. Bull. Plant. Prot. Service Japan*. **43**, 23–26 (2007).

[50] Fabre, F., Coville, J. & Cunniffe, N. J. Optimising reactive disease management using spatially explicit models at the landscape scale. in Plant Diseases and food Security in the 21st Century (eds (eds Scott, P., Strange, R., Korsten, L. & Gullino, M. L.) 47–72 (Springer International Publishing, (2021).

[51] Bassanezi, R. & Bassanezi, R. An approach to model the impact of Huanglongbing on citrus yield. in *Proceedings of the International Research Conference on Huanglongbing.* 301–304 (Plant Management Network).

[52] Djeddour, D., Pratt, C., Constantine, K., Rwomushana, I. & Day, R. The Asian Citrus Greening Disease (Huanglongbing): Evidence note on invasiveness and potential economic impacts for East Africa. *CABI Working Paper* 24 (2021).

[53] EFSA. A framework to substantiate absence of disease: the risk based estimate of system sensitivity tool (RiBESS) using data collated according to the EFSA Standard Sample Description-An example on *Echinococcus Multilocularis*. *EFSA Supporting Publications*. **9**, 366E (2012).

[54] Cannon, R. M. Demonstrating disease freedom—combining confidence levels. *Prev. Vet. Med.* **52**, 227–249. 10.1016/S0167-5877(01)00262-8 (2002). 11849719 10.1016/s0167-5877(01)00262-8

[55] Mitra, D. *Emerging Plant Diseases: Research Status and Challenges*. in *Emerging Trends in Plant Pathology* (eds Krishna P. Singh, Shamarao Jahagirdar, & Birinchi Kumar Sarma) 1–17Springer Singapore, (2021).

[56] Martinelli, F. et al. Advanced methods of plant disease detection. A review. *Agron. Sustain. Dev.* **35**, 1–25. 10.1007/s13593-014-0246-1 (2015).

[57] Park, A. W., Gubbins, S. & Gilligan, C. A. Invasion and persistence of plant parasites in a spatially structured host population. *Oikos* **94**, 162–174. 10.1034/j.1600-0706.2001.10489.x (2001).

[58] Choudhury, R. A. et al. Host density dependence and environmental factors affecting laurel wilt disease incidence. *Plant. Pathol.* **70**, 676–688. 10.1111/ppa.13314 (2021).

[59] Plantegenest, M., Le May, C. & Fabre, F. Landscape epidemiology of plant diseases. *J. Royal Soc. Interface*. **4**, 963–972 (2007). 10.1098/rsif.2007.1114 PMC2394555 17650471

[60] Velasquez-Camacho, L. et al. Remotely sensed Tree characterization in Urban areas: a review. *Remote Sens.* **13**, 4889 (2021).

[61] Maddison, A. C., Holt, J. & Jeger, M. J. Spatial dynamics of a monocyclic disease in a perennial crop. *Ecol. Model.* **88**, 45–52. 10.1016/0304-3800(95)00068-2 (1996).

[62] EFSA. Update of the scientific opinion on the risks to plant health posed by Xylella fastidiosa in the EU territory. *EFSA J.* **17**, e05665. 10.2903/j.efsa.2019.5665 (2019). 32626299 10.2903/j.efsa.2019.5665 PMC7009223

[63] Mundt, C. C. Use of the modified Gregory model to describe primary disease gradients of wheat leaf rust produced from area sources of inoculum. *Phytopathology*^®^**79**, 241–246 (1989).

[64] Ellis, J. et al. Developing epidemiological preparedness for a plant disease invasion: modelling citrus huánglóngbìng in the European Union. *bioRxiv*, 2024.2006.2004.597414 (2024). 10.1101/2024.06.04.597414

[65] Radici, A., Martinetti, D., Vanalli, C., Cunniffe, N. J. & Bevacqua, D. A metapopulation framework integrating landscape heterogeneity to model an airborne plant pathogen: the case of brown rot of peach in France. *Agric. Ecosyst. Environ.* **367**, 108994. 10.1016/j.agee.2024.108994 (2024).

[66] Nguyen, V. A., Bartels, D. W. & Gilligan, C. A. Modelling the spread and mitigation of an emerging vector-borne pathogen: Citrus greening in the U.S. *PLoS Comput. Biol.* **19**, e1010156. 10.1371/journal.pcbi.1010156 (2023). 37267376 10.1371/journal.pcbi.1010156 PMC10266658

[67] Martini, X., Pelz-Stelinski, K. S. & Stelinski, L. L. Absence of windbreaks and replanting citrus in solid sets increase density of Asian citrus psyllid populations. *Agric. Ecosyst. Environ.* **212**, 168–174. 10.1016/j.agee.2015.06.027 (2015).

[68] Craig, A. P., Cunniffe, N. J., Parry, M., Laranjeira, F. F. & Gilligan, C. A. Grower and regulator conflict in management of the citrus disease Huanglongbing in Brazil: a modelling study. *J. Appl. Ecol.* **55**, 1956–1965. 10.1111/1365-2664.13122 (2018).

[69] Filho, A. B. et al. The importance of primary inoculum and area-wide disease management to crop health and food security. *Food Secur.* **8**, 221–238. 10.1007/s12571-015-0544-8 (2016).

[70] Gottwald, T. et al. Canine olfactory detection of a vectored phytobacterial pathogen, Liberibacter Asiaticus, and integration with disease control. *Proc. Natl. Acad. Sci.* **117**, 3492–3501. 10.1073/pnas.1914296117 (2020). 32015115 10.1073/pnas.1914296117 PMC7035627

[71] Hao, H. Detection of citrus Huanglongbing by conventional and two fluorescence quantitative PCR assays. *Scientia Agricultura Sinica*. **39**, 2491–2497 (2006).

[72] Fu, S. M., Liu, H. W., Liu, Q. H., Zhou, C. Y. & Hartung, J. S. Detection of ‘Candidatus Liberibacter asiaticus’ in citrus by concurrent tissue print-based qPCR and immunoassay. *Plant. Pathol.* **68**, 796–803. 10.1111/ppa.12998 (2019).

[73] Morán, F., Herrero-Cervera, M., Carvajal-Rojas, S. & Marco-Noales, E. Real-time on-site detection of the three ‘Candidatus Liberibacter’ species associated with HLB disease: a rapid and validated method. *Front. Plant Sci.* **14**10.3389/fpls.2023.1176513 (2023). 10.3389/fpls.2023.1176513 PMC10282772 37351204

