# Supplementary material for: Assessing delimiting strategies to identify the infested zones of quarantine plant pests and diseases
Source: Sci Rep. 2025 Feb 15;15:5610. doi: 10.1038/s41598-025-90343-2 (PMC11829978; doi:10.1038/s41598-025-90343-2)
Supplement: Supplementary file 2 — Supplementary Material 2 [file 41598_2025_90343_MOESM2_ESM.docx]

Supplementary Materials

**Manuscript title: Assessing delimiting strategies to identify the infested zones of quarantine plant pests and diseases**

**Authors: Koh, J. M. J., Cunniffe, N. J., & Parnell, S.**

# Description of Scenarios

|  | **IE Duration of pathogen spread** | **IE Estimated spread distance** | **Strategy starting point** | **Asymptomatic period** | **Method sensitivity** |
| --- | --- | --- | --- | --- | --- |
| Scenario 1 | Matched (5 years or 25 generations) | Matched (1050m/year or 750m/gen) | Centre | None | 0.2, 0.5, 0.8, 1.0 |
| Scenario 2 | Matched (5 years or 25 generations) | Matched (1050m/year or 750m/gen) | Centre | 365 days | 0.2, 0.5, 0.8, 1.0 |
| Scenario 3 | Matched (5 years or 25 generations) | Matched (1050m/year or 750m/gen) | Random symptomatic tree | 365 days | 0.2, 0.5, 0.8, 1.0 |
| Scenario 4 | Overestimated, matched and underestimated | Year: 450m, 750, 1050m, 1350m  Gen: 350m, 550m, 750m, 1050m | Random symptomatic tree | 365 days | 0.2, 0.5, 0.8, 1.0 |

Scenarios 1 to 4 were applied to random host distributed landscapes. When assessing the performance of the delimiting strategies on clustered or extreme clustered landscapes (Scenario 5), a modified Scenario 4 was used, where the Method Sensitivity was fixed at 0.5.

# Detailed Explanation on the Effect of Pest Spread Duration

With the exception of the Gamma Gen strategies, the Capability scores of the delimiting strategies improved when the estimated pest duration was matched or underestimated. The performance of all the delimiting strategies can essentially be explained by their radii length. Here are some examples to aid in the explanation. We will start with the Gamma Year strategies which also explain the behaviour of the Linear strategies.


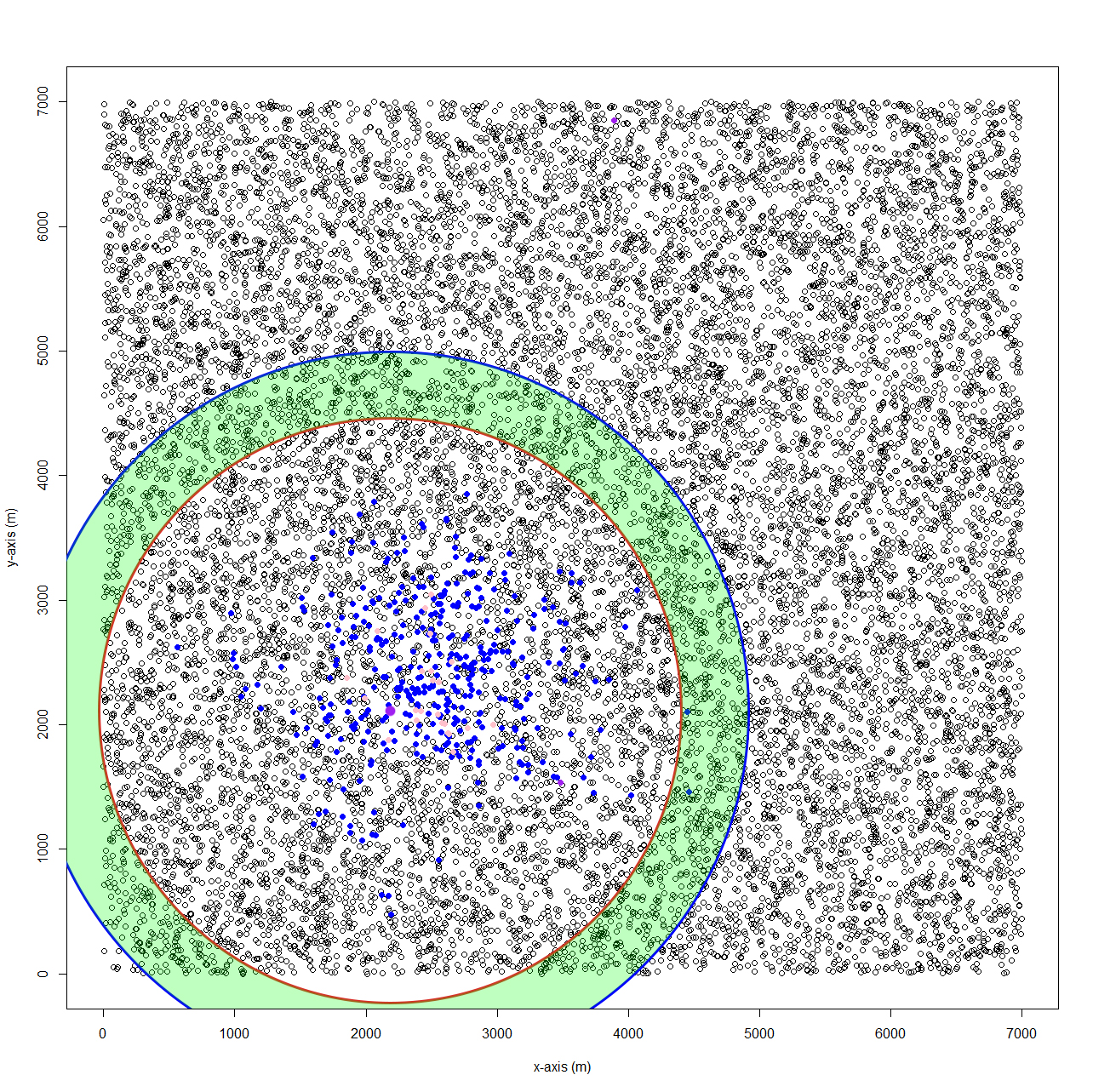


Supplementary Figure 1: First survey round of the Adaptive (Gamma Year) strategy. The method sensitivity is 0.5, the IE annual maximum spread distance was matched with the true annual maximum spread distance (1050 m/year), and the IE pest spread duration was overestimated (IE pest spread duration was 3 years when the pest had only been spreading for 2 years). The blue outer circle shows the outer boundary of the survey band (green shaded area), the red inner circle shows the inner boundary of the survey band, small black circles are susceptible trees, blue-filled circles are asymptomatic trees, pink-filled circles are symptomatic trees and the purple-filled circle was the first symptomatic tree detected (i.e. the starting point of the delimiting strategy).

In this first example (Supplementary Figure 1), although it only shows the Adaptive (Gamma Year) strategy, the reasons behind its performance are the same for the Linear and In-to-Out (Gamma Year) strategies. Because the spread duration has been overestimated, the first round of survey (green shaded area) is conducted much further from the actual extent of the pest spread, so no detection was made, and the strategy moves inwards.


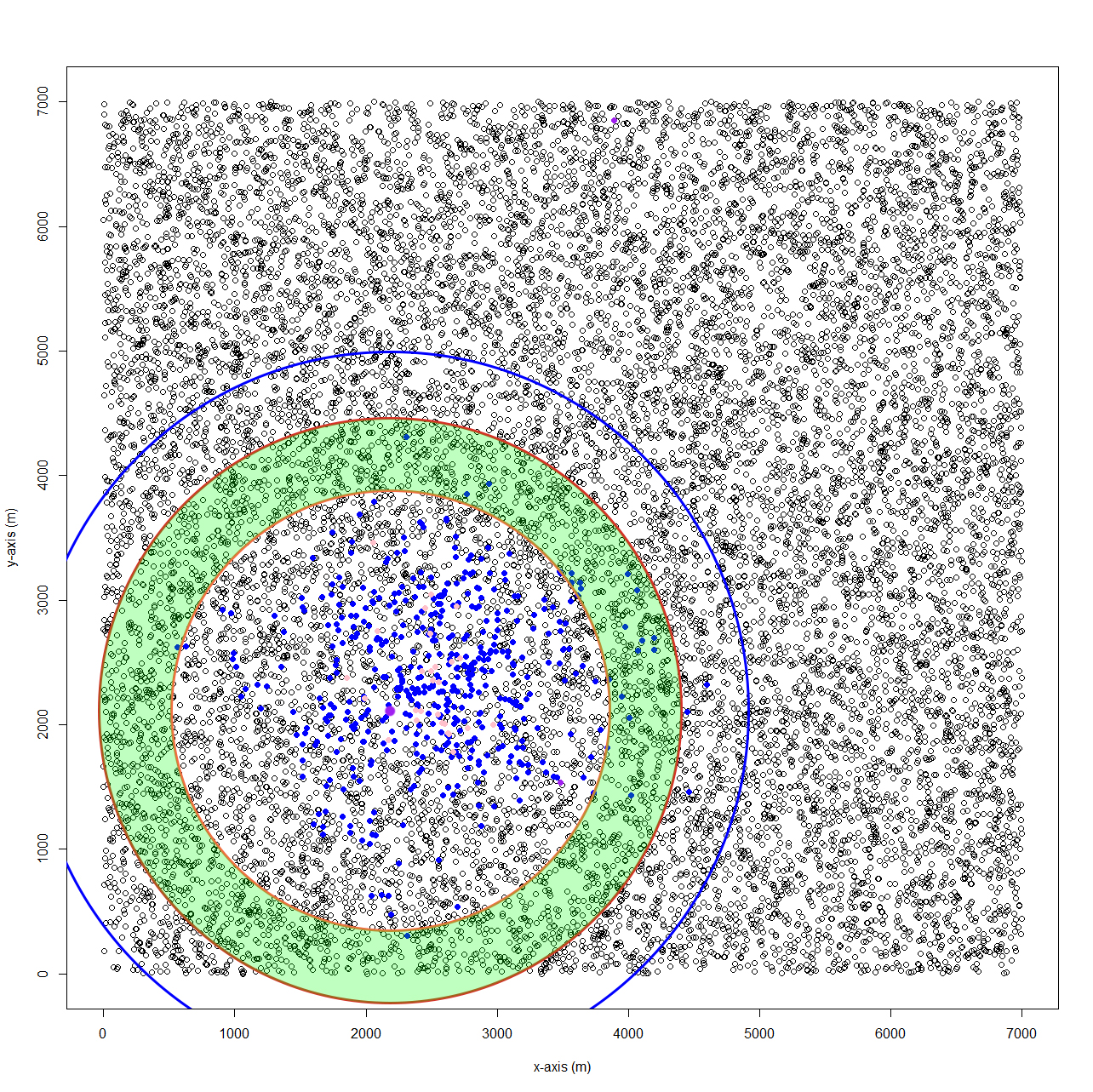


Supplementary Figure 2: Second survey round of the delimiting strategy shown in Figure 1. The scenario, variables and legend are the same as Scenario 1.

In the second survey round (Supplementary Figure 2), you can see that some infected hosts occur in the survey band, but they are asymptomatic (blue dots), so no successful detection was made, and the strategy moves inwards again.


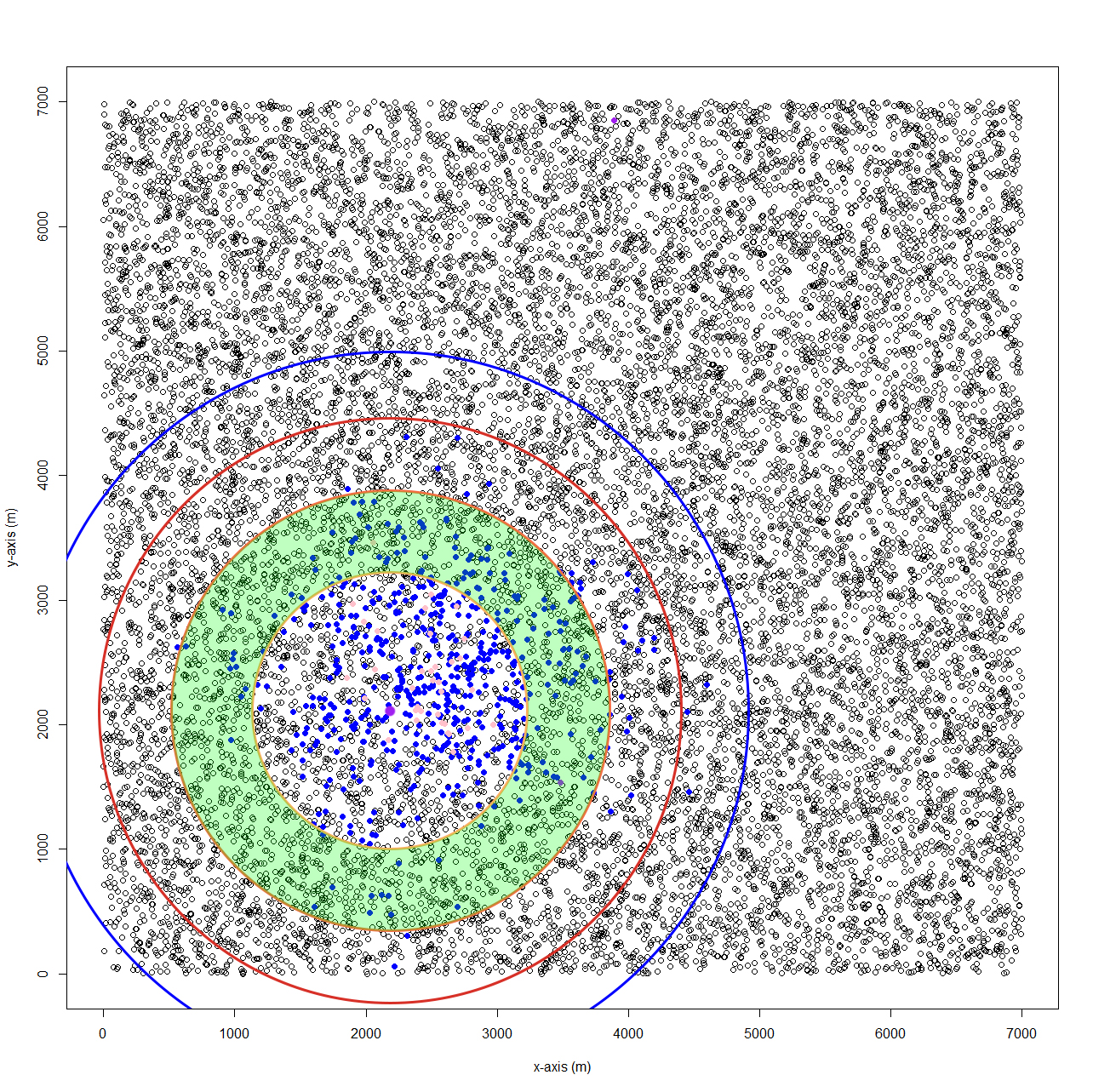


Supplementary Figure 3: Third survey round of the delimiting strategy shown in Figure 1. The scenario, variables and legend are the same as Scenario 1.

In the third survey round (Supplementary Figure 3), although there is at least one symptomatic host (pink dots), it was not detected (below the design prevalence of 0.01) and the Adaptive (Gamma Year) strategy moves further inward.


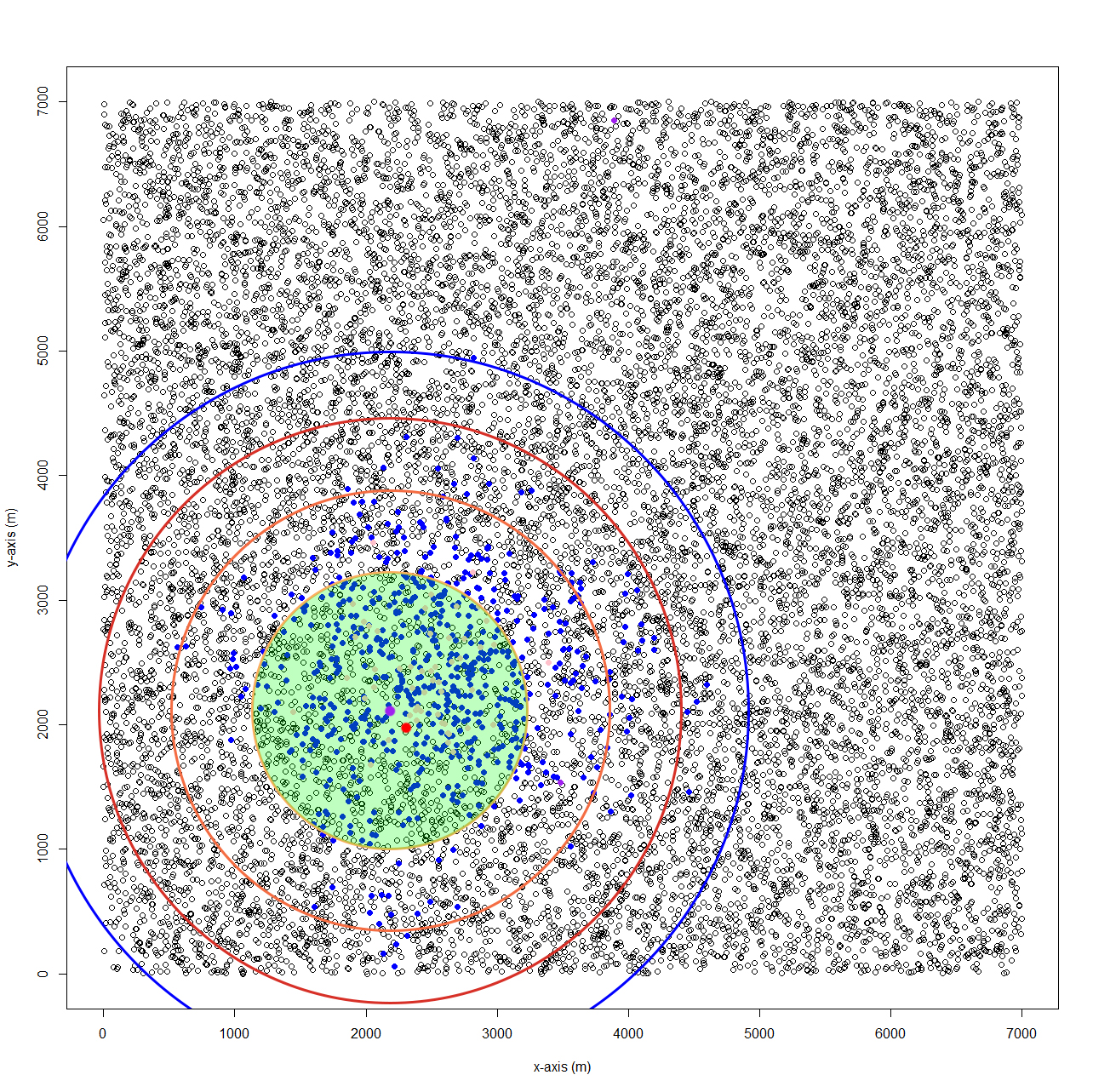


Supplementary Figure 4: Fourth survey round of the delimiting strategy shown in Figure 1. The scenario, variables and legend are the same as Scenario 1. The red-filled circle shows a successful detection of a symptomatic tree.

In the fourth and final round of survey (Supplementary Figure 4), there are sufficient symptomatic hosts, a successful detection was made, and the strategy stops. The potential infested zone is the same as the shaded green area and the final capability score was 0.67. From this example, you can see that the performance of the Adaptive (Gamma Year) strategy was mainly affected by its small radii which were not large enough to include the asymptomatic hosts (this will become clearer when we look at the Gamma Gen strategies). You could also argue that the strategy’s performance was affected by the scarcity of symptomatic hosts. However, this “delayed” spread is typical of HLB. This is also why we noted in the discussion that our findings would likely differ if a different pest were simulated. Under the exact same scenario and starting point, the Adaptive (Gamma Year) strategy, still hampered by its relatively small radii, achieved capability scores of 0.80 and 0.89 when the duration of pest spread was accurately estimated or underestimated, respectively. In both cases, the strategy expanded outward as the delay allowed sufficient time for the simulated pest to "ramp up" its spread, leading to an increase in detectable symptomatic hosts.


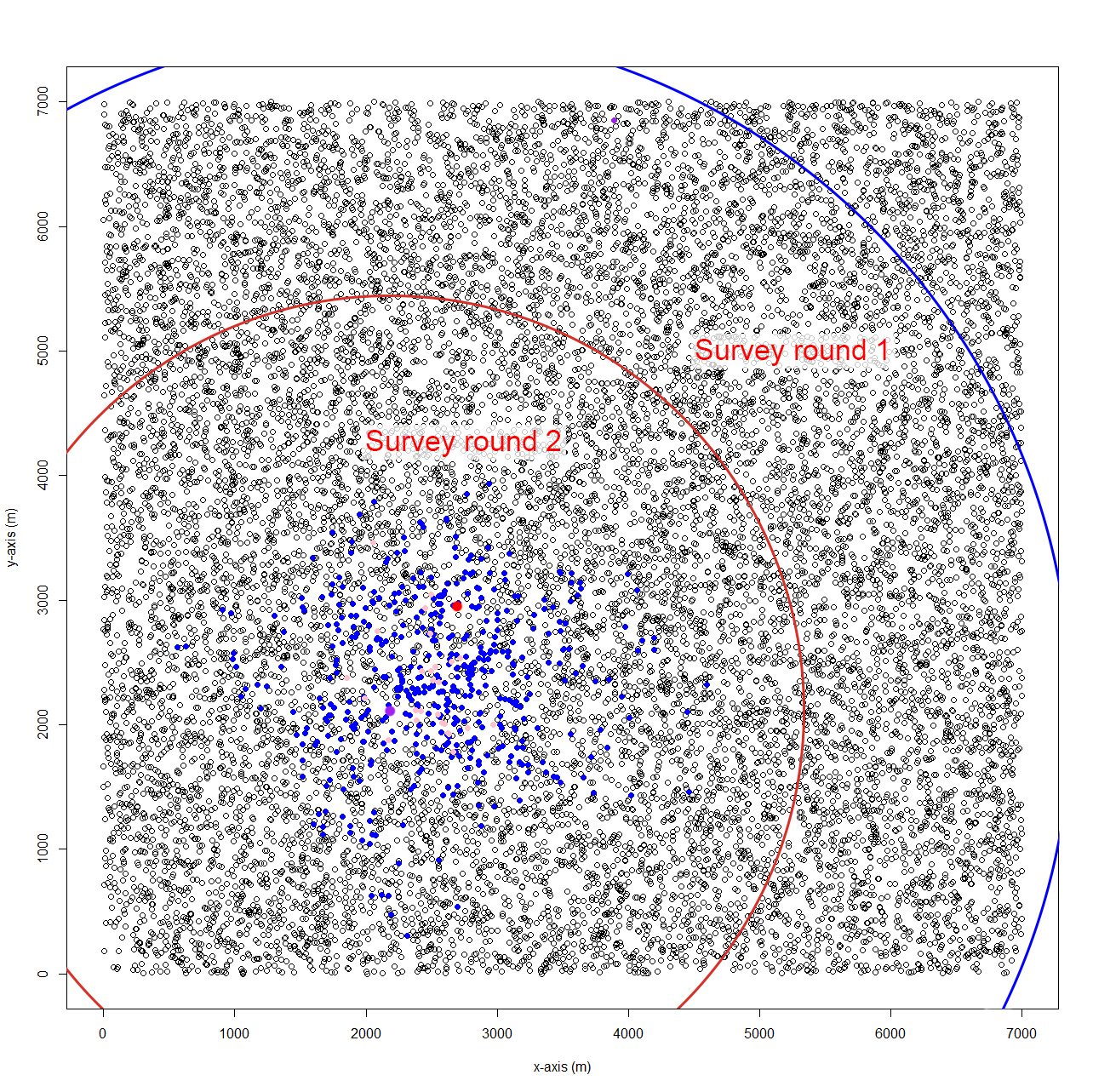


Supplementary Figure 5: An example of the Adaptive (Gamma Gen) strategy in the same scenario and starting point as the Adaptive (Gamma Year) strategy in Figures 1-4. IE generational spread distance was matched with the true value (750 m/gen). Legend remains the same as the previous figures.

In contrast, when the Adaptive (Gamma Gen) strategy was applied to the same scenario, it achieved a capability of 1. As seen from Supplementary Figure 5, its radii are large enough to encompass the extent of the pest spread which included both asymptomatic and symptomatic hosts (Note that the symptomatic and asymptomatic status of the hosts in Supplementary Figure 5 reflect their status at the end of the delimiting strategy). While it may seem like the large area covered would result in poor Effort scores, only 894 hosts were inspected for this example (this is due to the effect of the RiBESS+ equation).


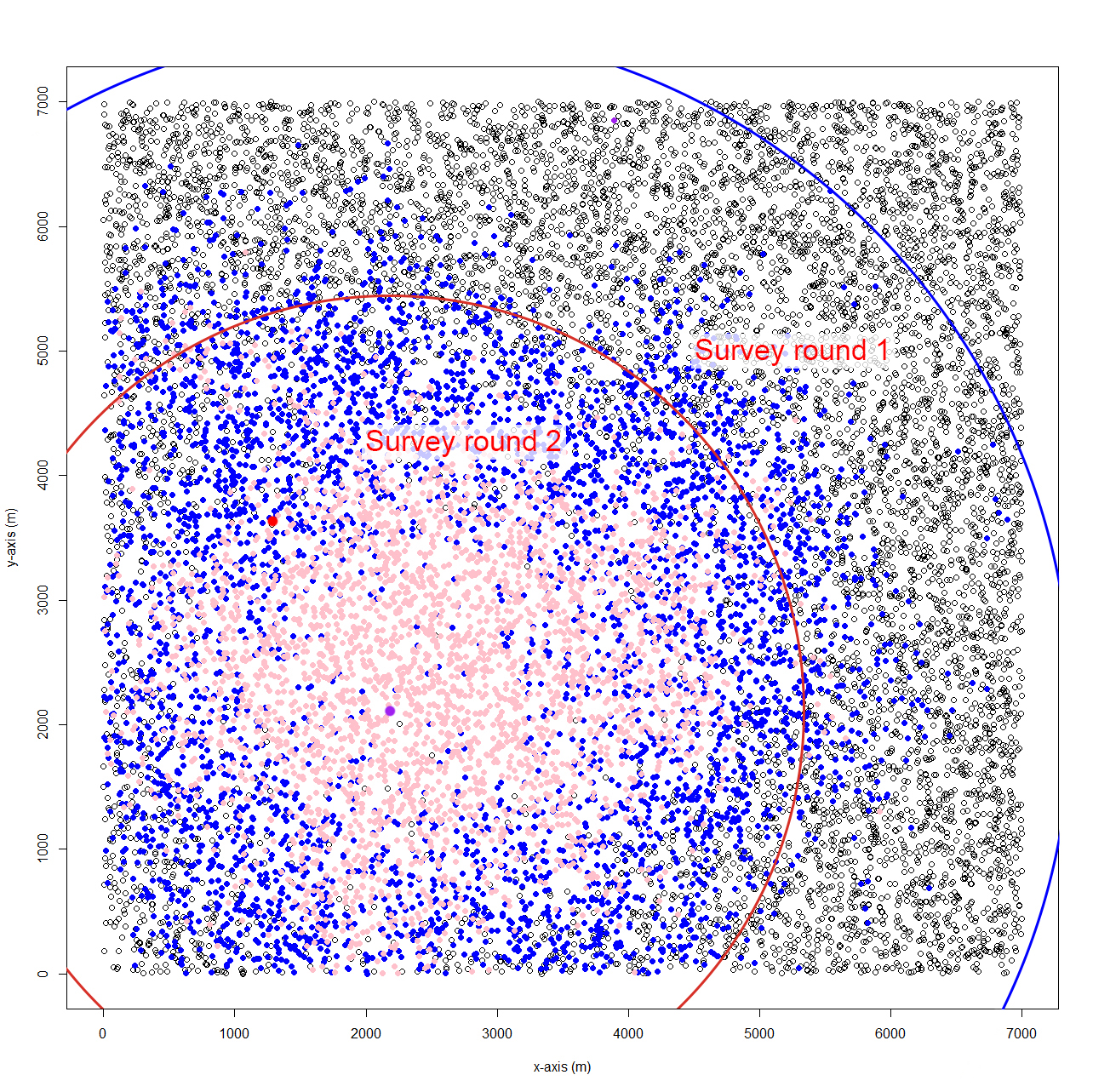


Supplementary Figure 6: An example of the Adaptive (Gamma Gen) strategy in the same scenario as shown in Figure 5, except that the IE pest spread duration was underestimated (IE pest spread duration was estimated to be 3 years when the pest had been spreading for 4 years). Legend remains the same as in previous figures.

Supplementary Figure 6 shows the exact same scenario as the previous example except that the duration of pest spread was underestimated. The Adaptive (Gamma Gen) strategy achieved a capability score of only 0.94, primarily because, under these circumstances, its radius was not sufficiently large to contain and delimit all infected hosts.

In summary, the Gamma Gen strategies performed well when the duration of pest spread was overestimated, as their larger radii were able to encompass both symptomatic and asymptomatic hosts. However, as the pest spread duration increased, the relative capability of these radii decreased, leading to a decline in performance. Conversely, the Gamma Year and Linear strategies consistently had radii that were insufficient to encompass both symptomatic and asymptomatic hosts. Their performance improved with longer pest spread durations due to the higher number of symptomatic hosts, which facilitated more successful detections. As for the Multi-foci strategy, its performance is also explained by the increased number of successful detections with longer pest spread durations.

## Supplementary Figures


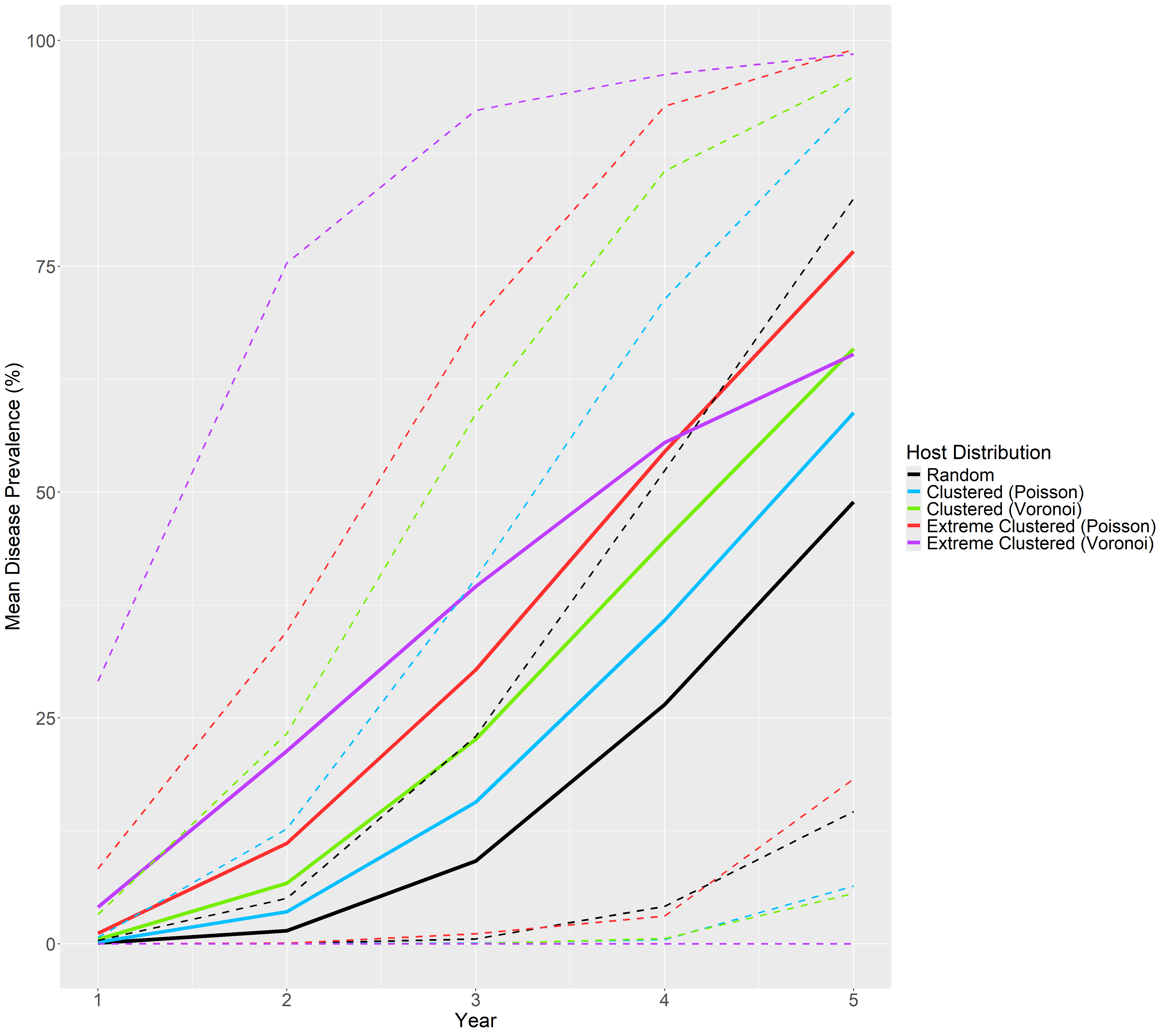


Figure S1: The change in mean disease prevalence of the host landscape types generated with the three different host distribution methods and with different levels of clustering. The dotted lines represent the 95% quartile range which are estimated from 500 realizations for each year.


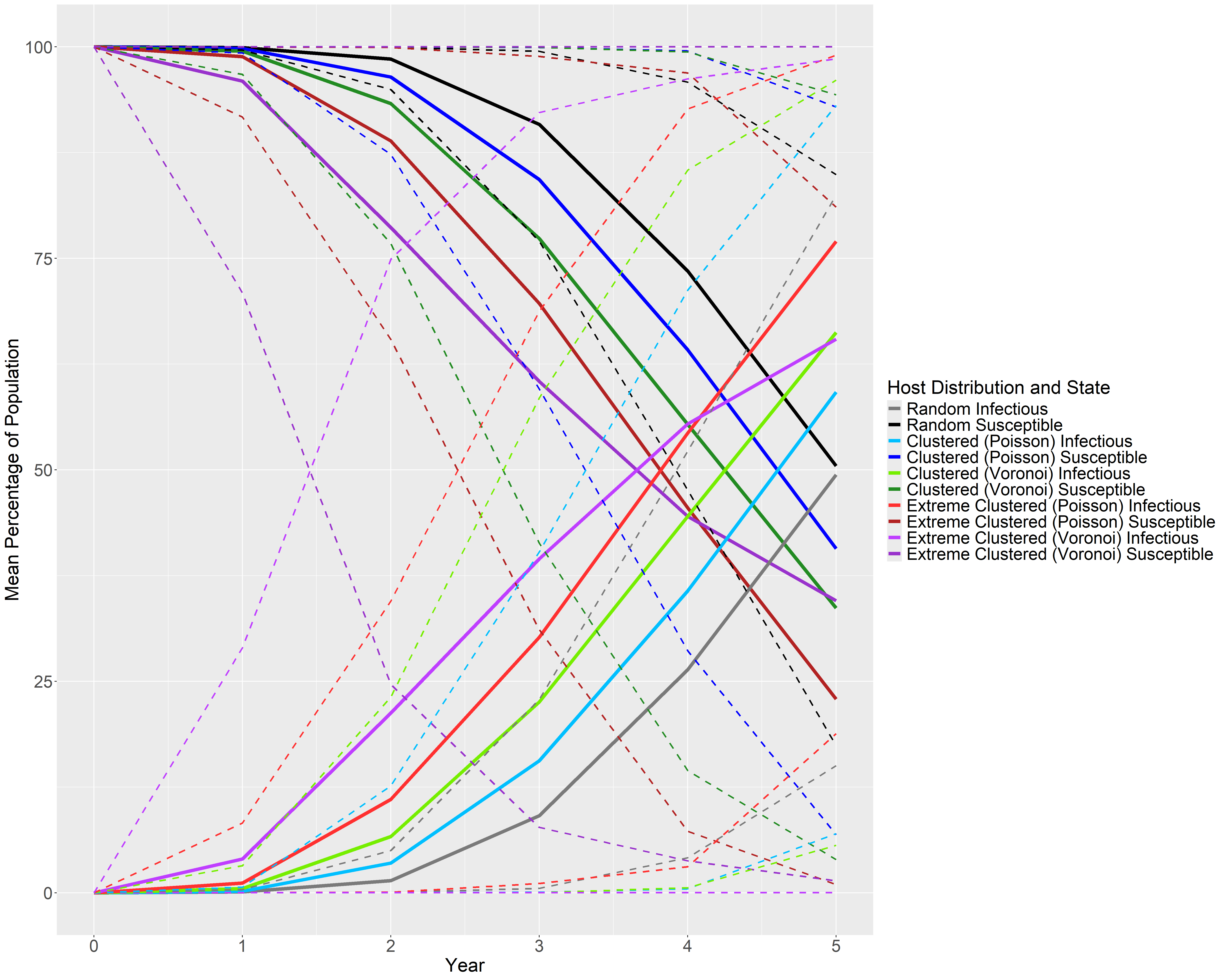


Figure S2: Graph showing the change in the percentage of susceptible and infectious hosts in the population for each of the host landscape types. Dotted lines represent the 95% quartile range which are estimated from 500 realizations for each year.


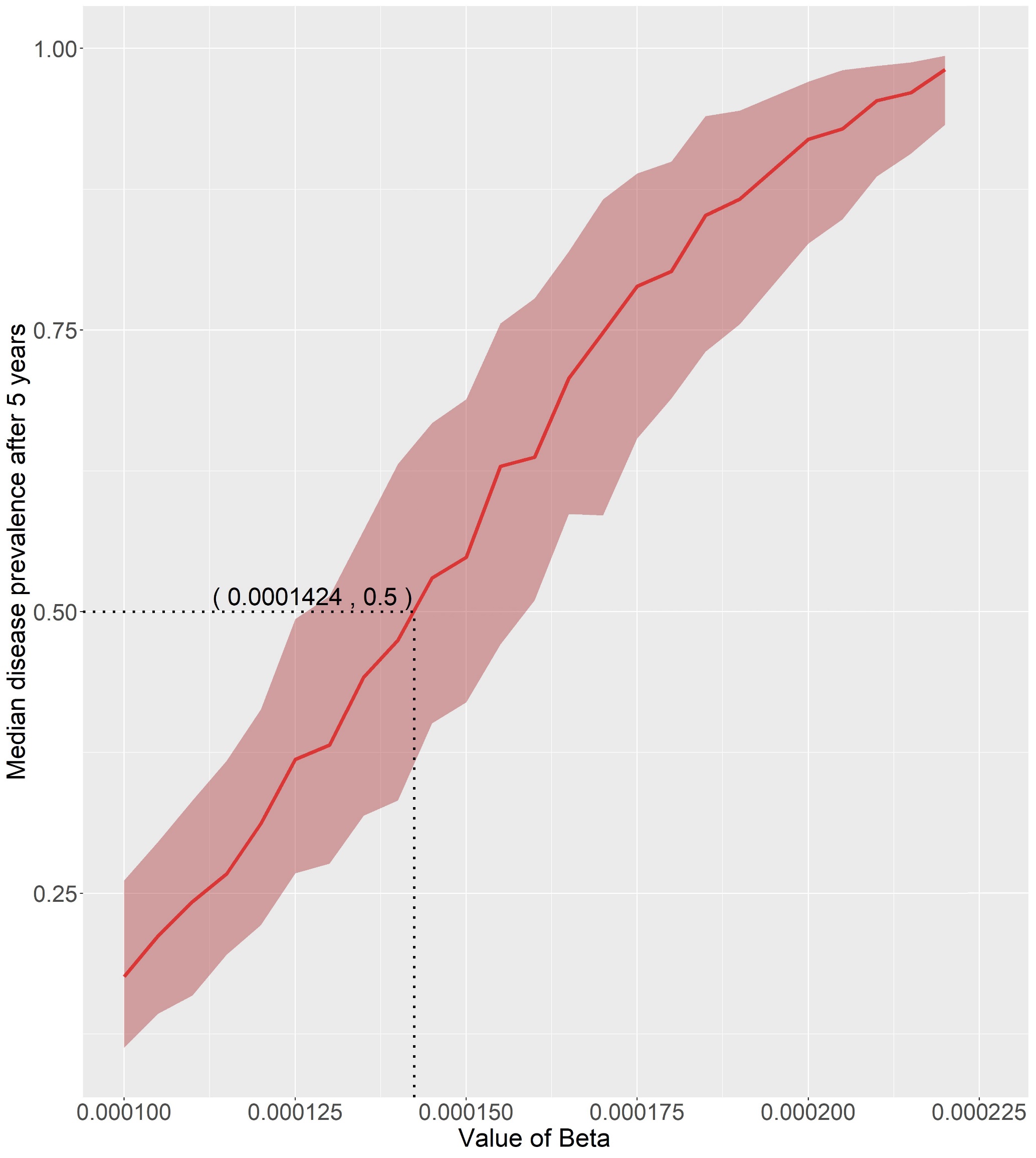


Figure S3: Graph showing the results of the line method used to parameterize the baseline infection rate ($\beta$) to achieve a median prevalence level of 50% after 5 years. Error envelope represents the interquartile range.


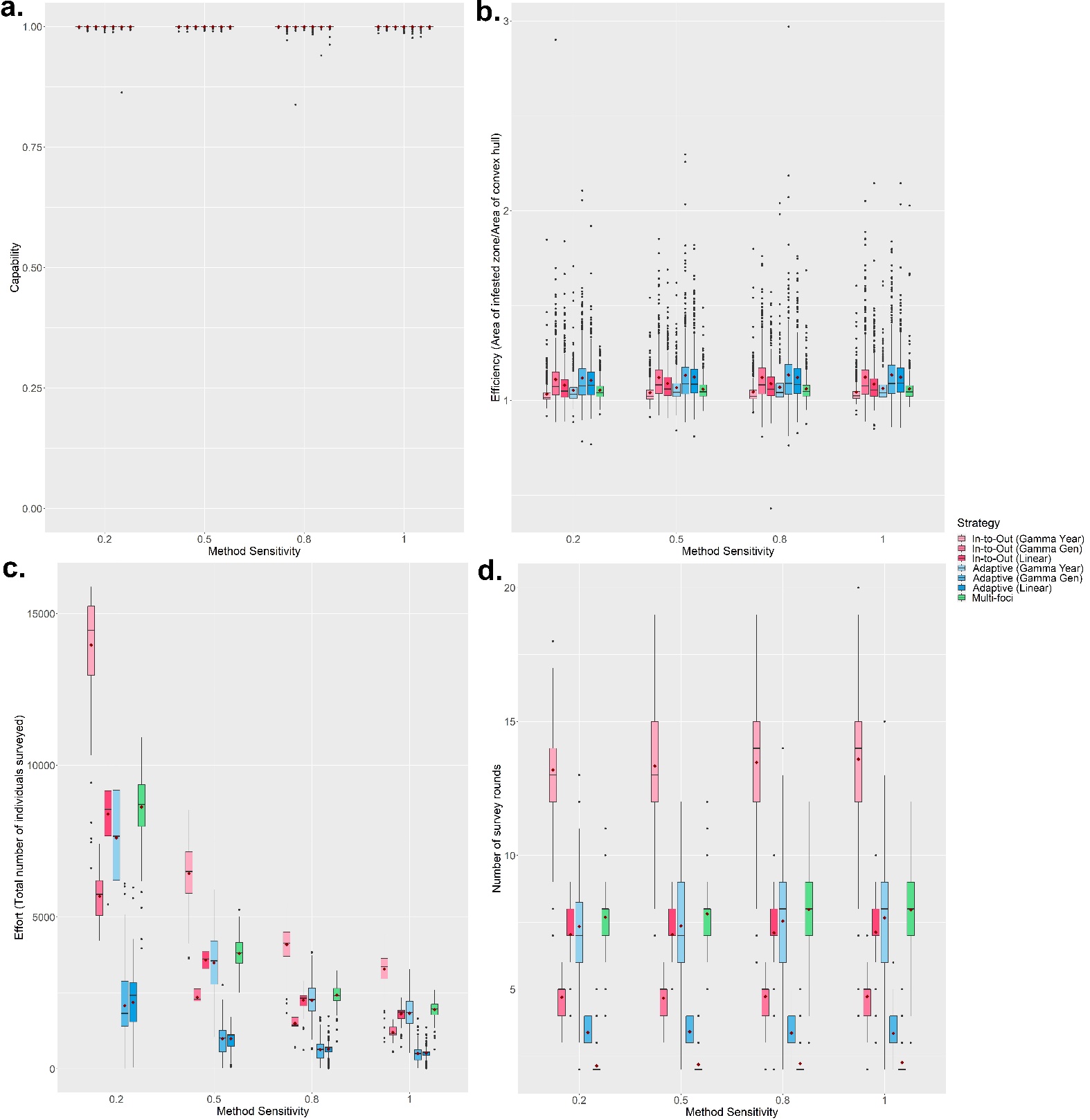


Figure S4: The change in Capability (a), Efficiency (b), Effort (c) scores, and Number of Survey Rounds (d) with Method Sensitivity of each delimiting strategy on a random host landscape in Scenario 1. The inspector-estimated spread distances matched the true values (750 m/generation and 1050 m/year). For each realization, all delimiting strategies started from the origin of the epidemic, no asymptomatic period was included, and the inspector-estimated duration of the pest spread was matched with the true value (3 years). Boxplots were obtained from 500 iterations. Mean values are indicated with a dark red diamond.


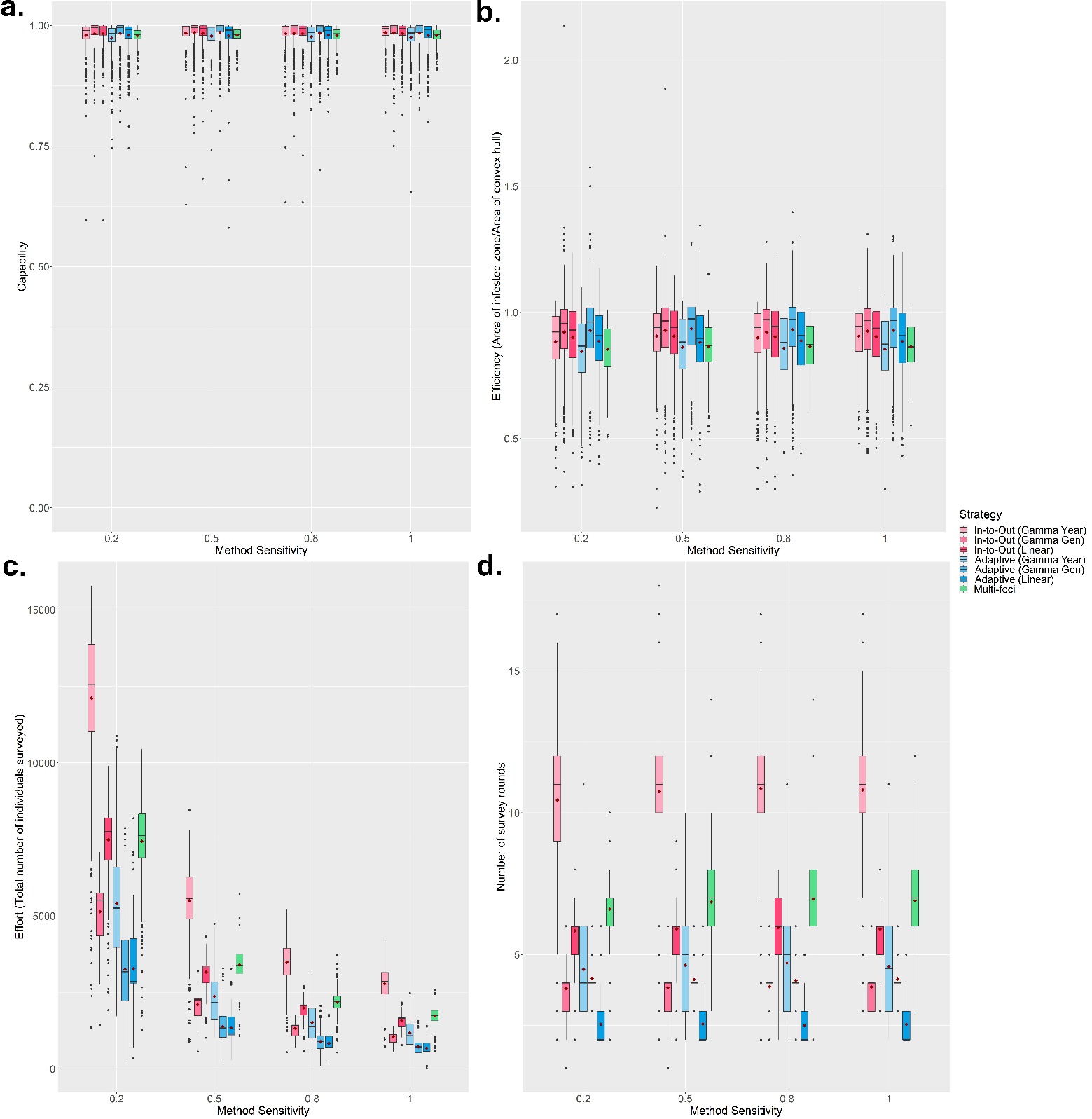


Figure S5: The change in in Capability (a), Efficiency (b), Effort (c) scores, and Number of Survey Rounds (d) with Method Sensitivity of each delimiting strategy on a random host landscape in Scenario 2. The inspector-estimated spread distances matched the true values (750 m/generation and 1050 m/year). For each realization, all delimiting strategies started from the origin of the epidemic, a 1-year asymptomatic period was included, and the inspector-estimated duration of the pest spread was matched with the true value (3 years). Boxplots were obtained from 500 iterations. Mean values are indicated with a dark red diamond.


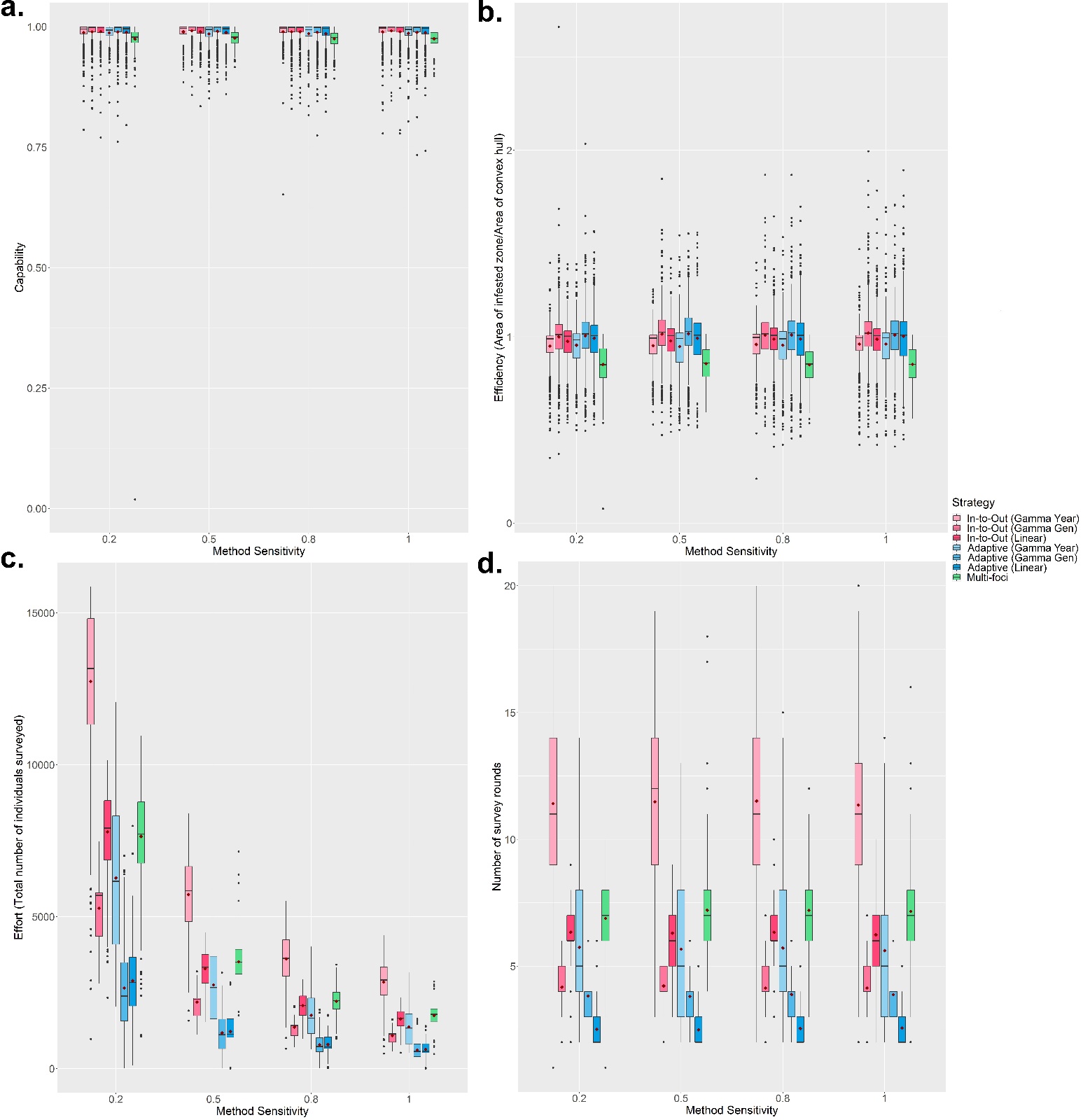


Figure S6: The change in Capability (a), Efficiency (b), Effort (c) scores, and Number of Survey Rounds (d) with Method Sensitivity of each delimiting strategy on a random host landscape in Scenario 3. The inspector-estimated spread distances matched the true values (750 m/generation and 1050 m/year). For each realization, all delimiting strategies started from the same randomly selected symptomatic individual, a 1-year asymptomatic period was included, and the inspector-estimated duration of the pest spread was matched with the true value (3 years). Boxplots were obtained from 500 iterations. Mean values are indicated with a dark red diamond.


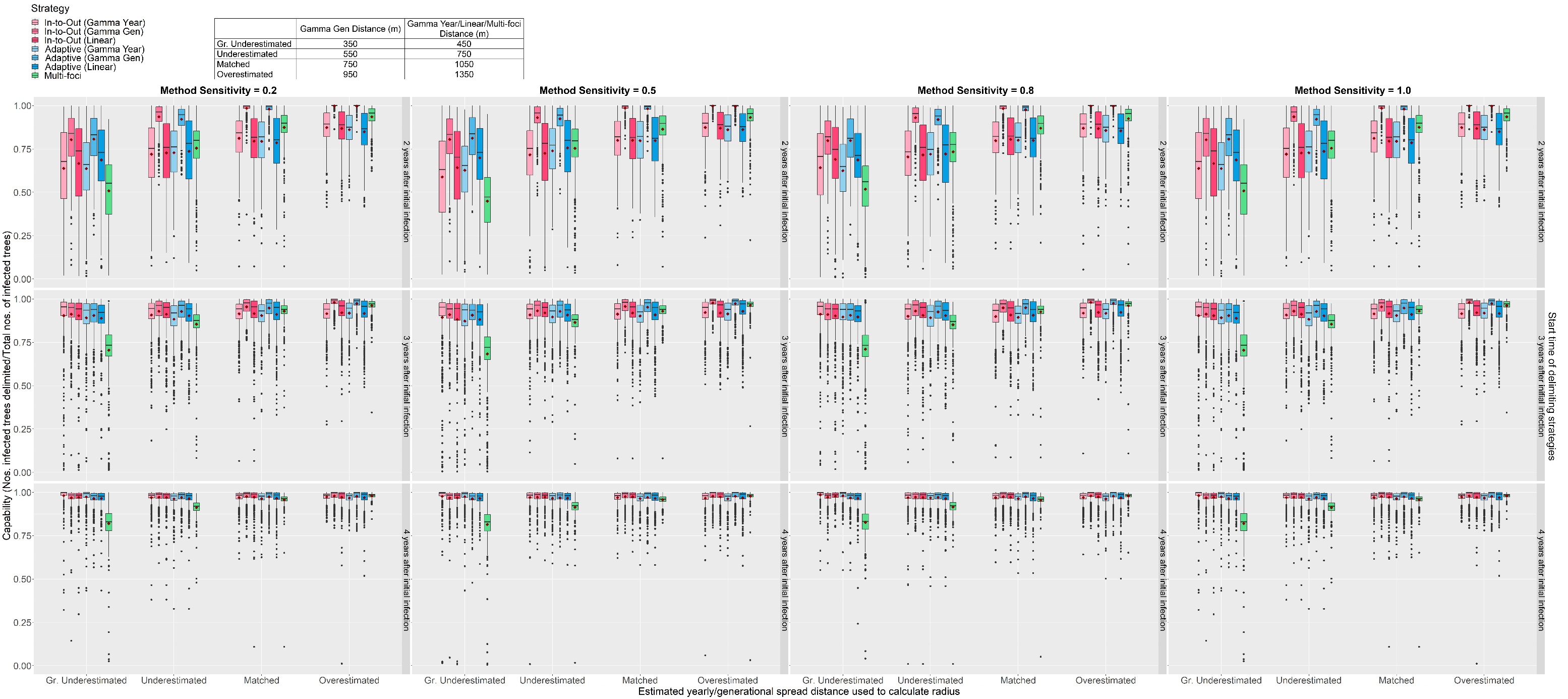


Figure S7: The change in Capability scores of all seven delimiting strategies on a random host landscape in Scenario 4 with Method Sensitivity, inspector-estimated spread distances and duration of pest spread. For each realization, all delimiting strategies started from the same randomly selected symptomatic individual, and a 1-year asymptomatic period was included. Boxplots were obtained from 500 iterations. Mean values are indicated with a dark red diamond.


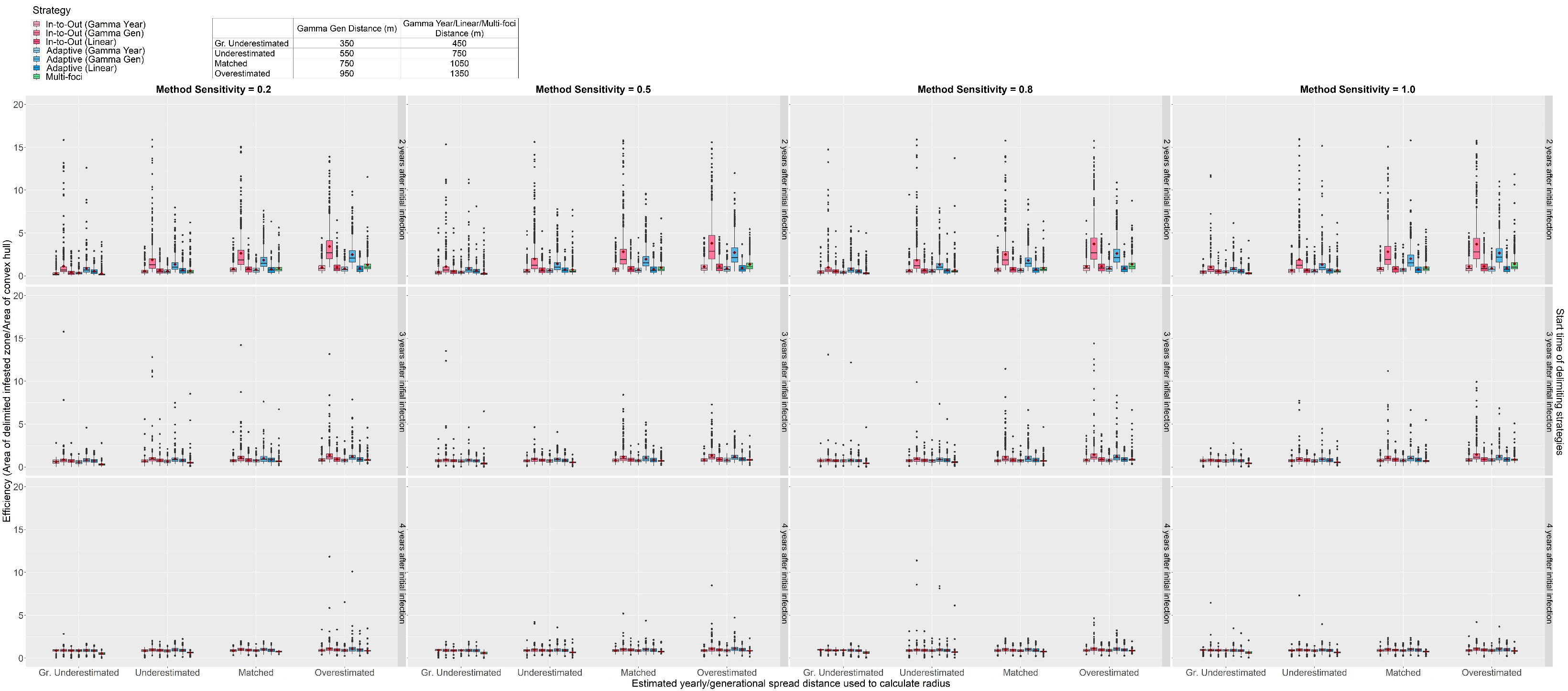


Figure S8: The change in Efficiency scores of all seven delimiting strategies on a random host landscape in Scenario 4 with Method Sensitivity, inspector-estimated spread distances and duration of pest spread. For each realization, all delimiting strategies started from the same randomly selected symptomatic individual, and a 1-year asymptomatic period was included. Boxplots were obtained from 500 iterations. Mean values are indicated with a dark red diamond.


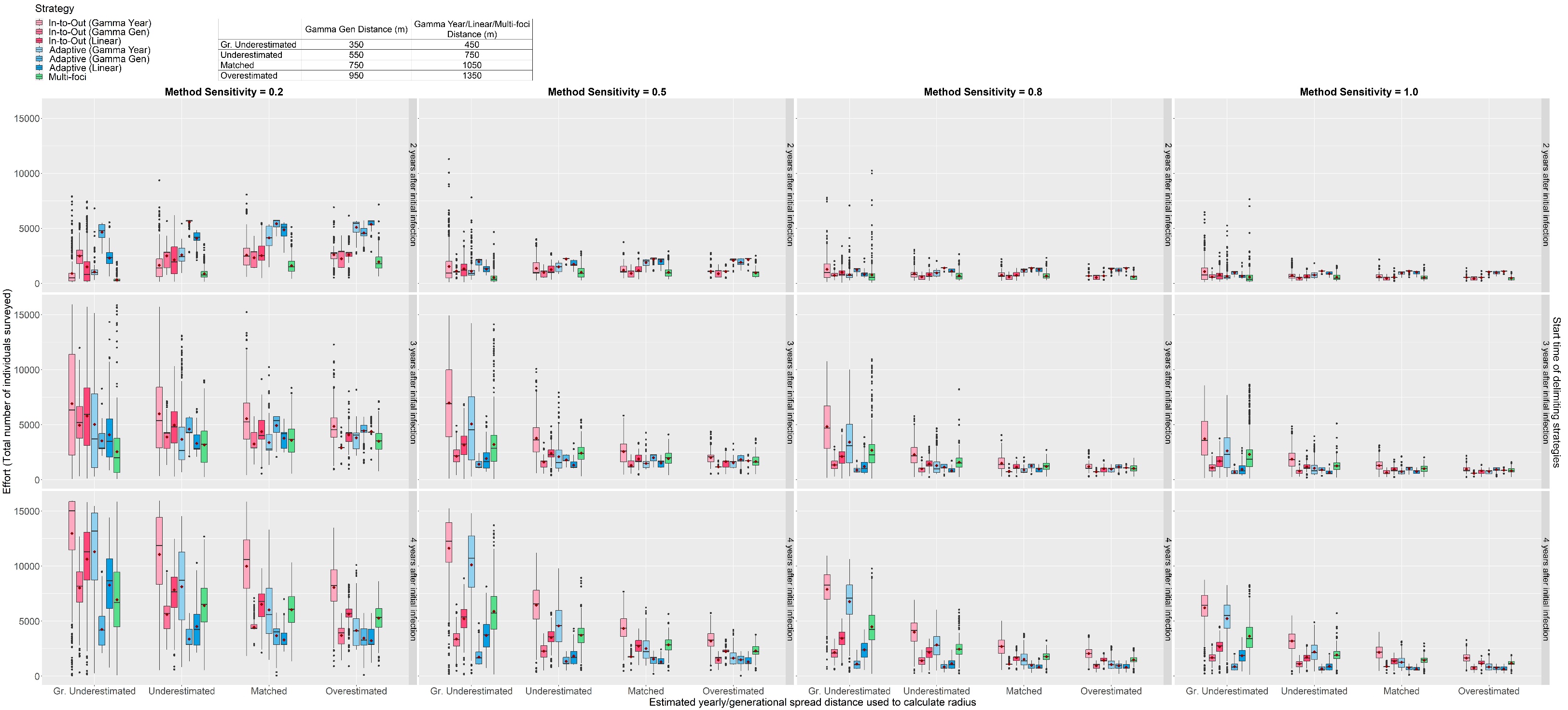


Figure S9: The change in Effort scores of all seven delimiting strategies on a random host landscape in Scenario 4 with Method Sensitivity, inspector-estimated spread distances and duration of pest spread. For each realization, all delimiting strategies started from the same randomly selected symptomatic individual, and a 1-year asymptomatic period was included. Boxplots were obtained from 500 iterations. Mean values are indicated with a dark red diamond.


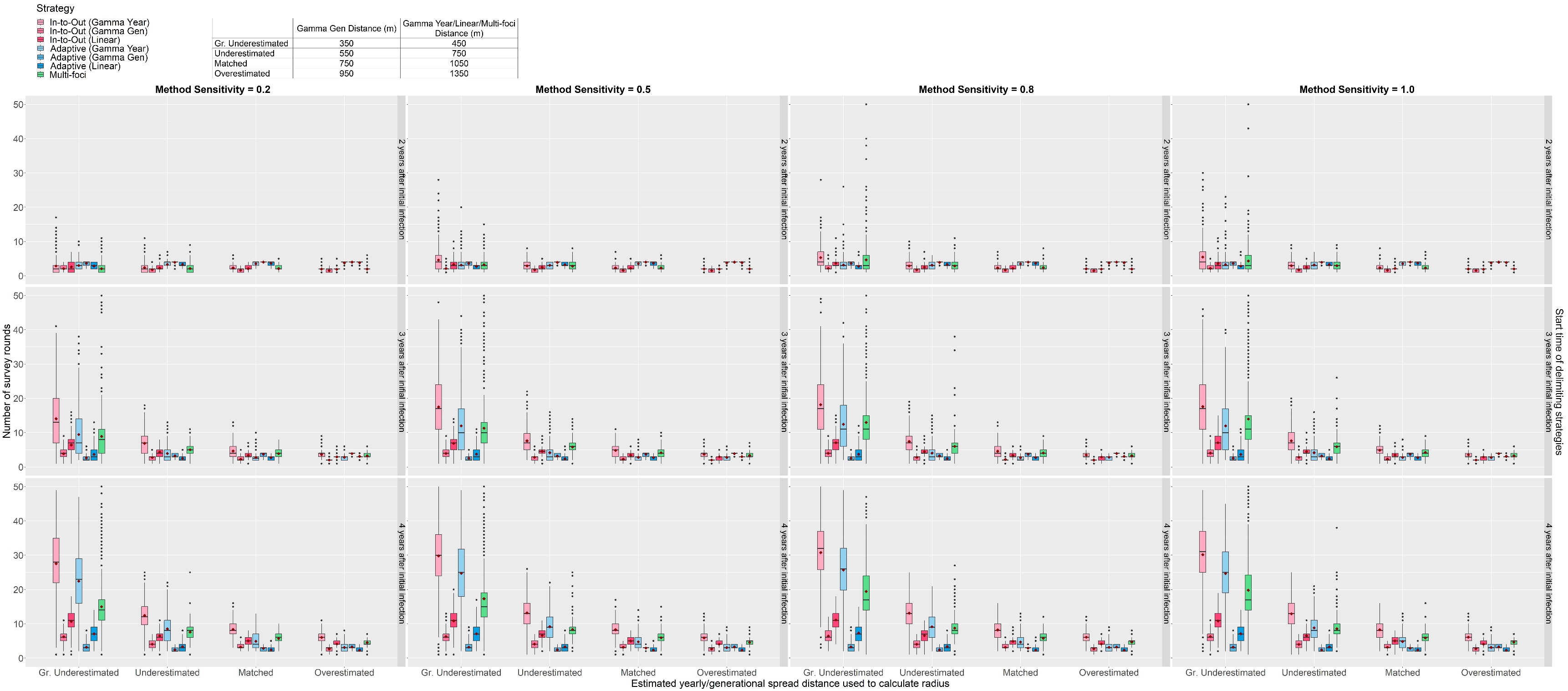


Figure S10: The change in the number of survey rounds each delimiting strategy took to delimit the potential infested zone on a random host landscape in Scenario 4 with Method Sensitivity, inspector-estimated spread distances and duration of pest spread. For each realization, all delimiting strategies start from the same randomly selected symptomatic individual, and a 1-year asymptomatic period was included. Boxplots were obtained from 500 iterations. Mean values are indicated with a dark red diamond.


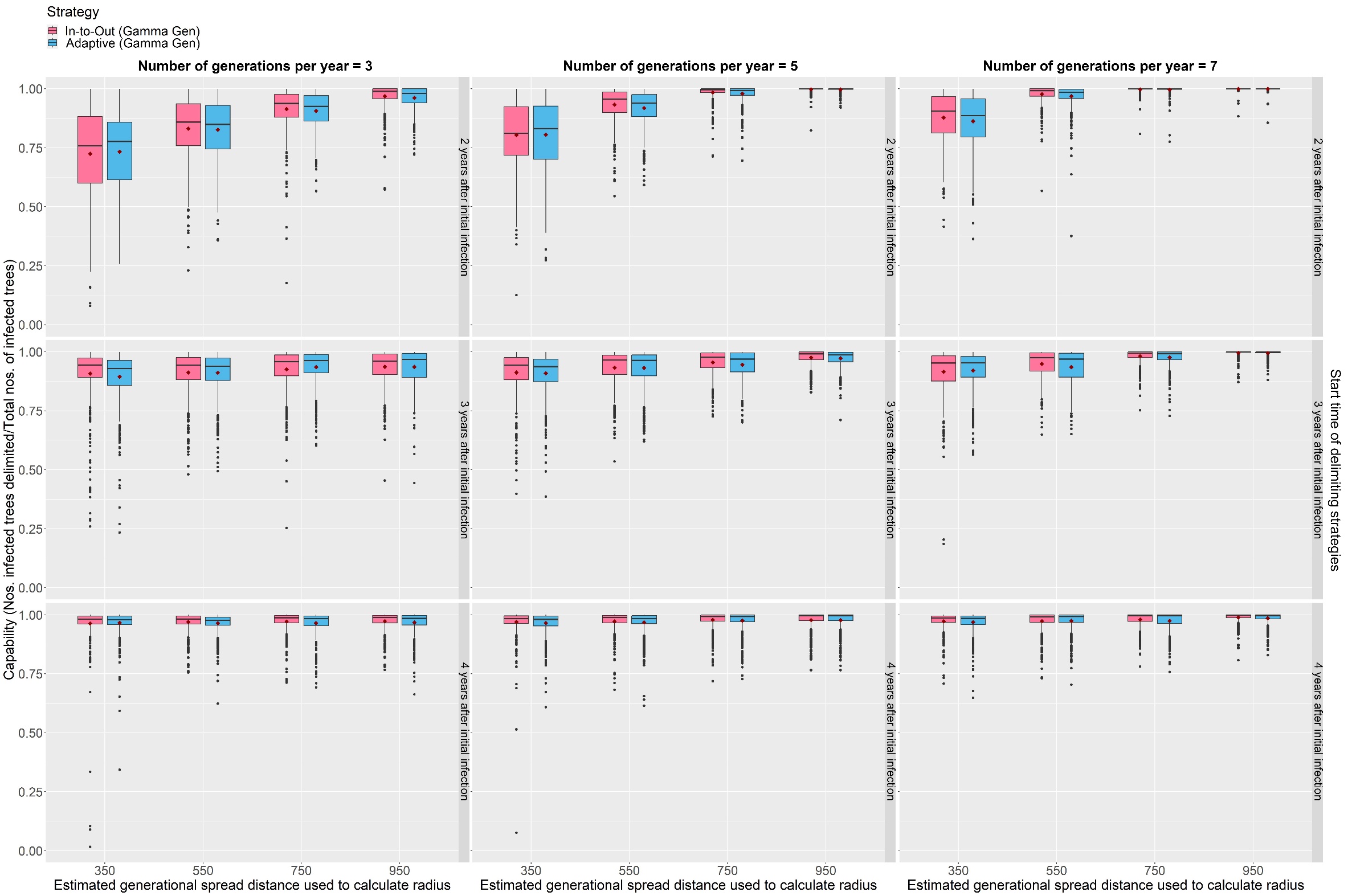


Figure S11: The change in Capability scores of the In-to-Out (Gamma Gen) and Adaptive (Gamma Gen) strategies on a random host landscape with varying inspector-estimated generational spread distances, mean number of generations per year and duration of pest spread. For each realization, both delimiting strategies started from the same randomly selected symptomatic individual, and a 1-year asymptomatic period was included. Method sensitivity was kept constant at 0.5. Boxplots were obtained from 500 iterations. Mean values are indicated with a dark red diamond.


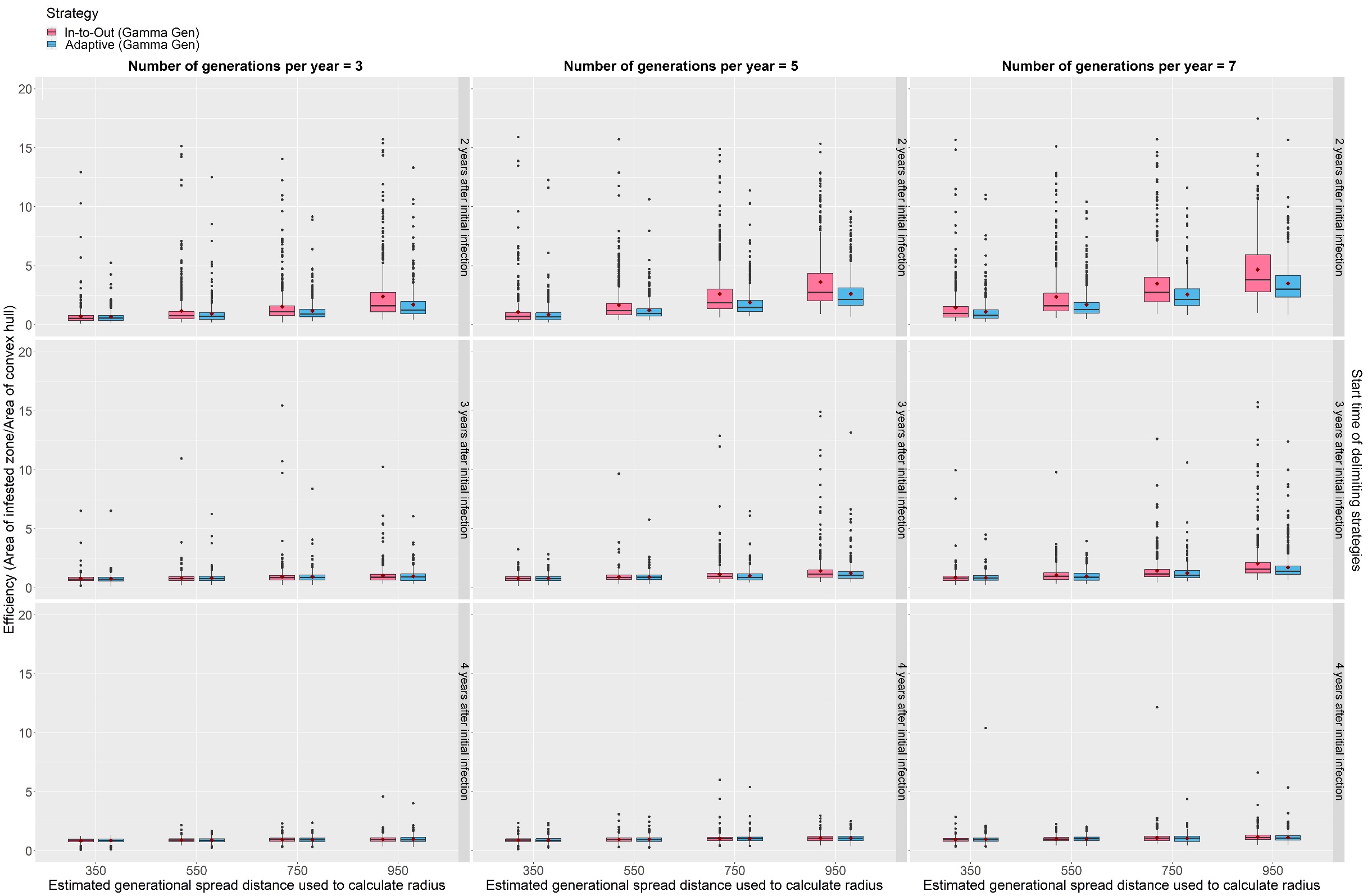


Figure S12: The change in Efficiency scores of the In-to-Out (Gamma Gen) and Adaptive (Gamma Gen) strategies on a random host landscape with varying inspector-estimated generational spread distances, mean number of generations per year and duration of pest spread. For each realization, both delimiting strategies started from the same randomly selected symptomatic individual, and a 1-year asymptomatic period was included. Method sensitivity was kept constant at 0.5. Boxplots were obtained from 500 iterations. Mean values are indicated with a dark red diamond.


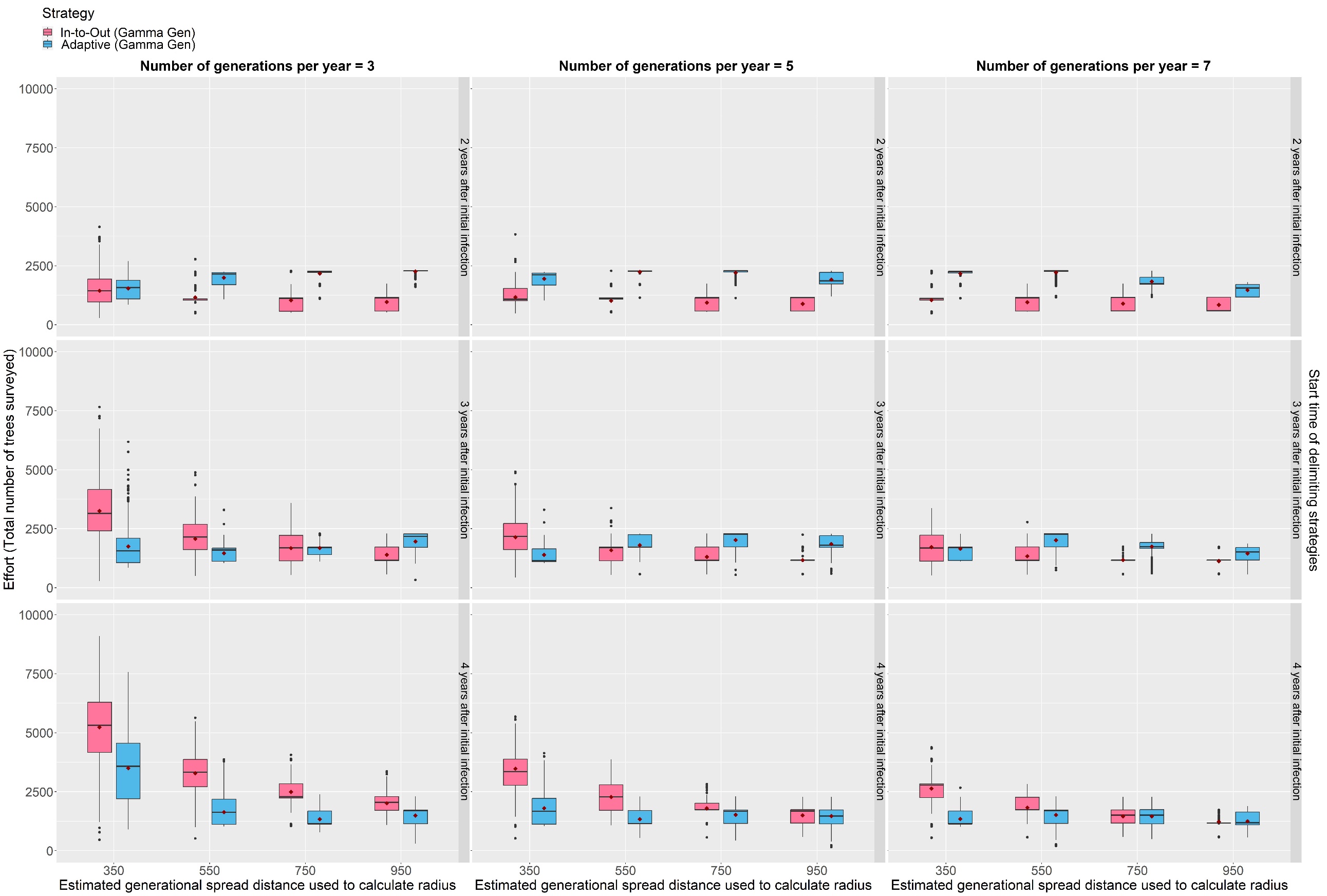


Figure S13: The change in Effort scores of the In-to-Out (Gamma Gen) and Adaptive (Gamma Gen) strategies on a random host landscape with varying inspector-estimated generational spread distances, mean number of generations per year and duration of pest spread. For each realization, both delimiting strategies started from the same randomly selected symptomatic individual, and a 1-year asymptomatic period was included. Method sensitivity was kept constant at 0.5. Boxplots were obtained from 500 iterations. Mean values are indicated with a dark red diamond.


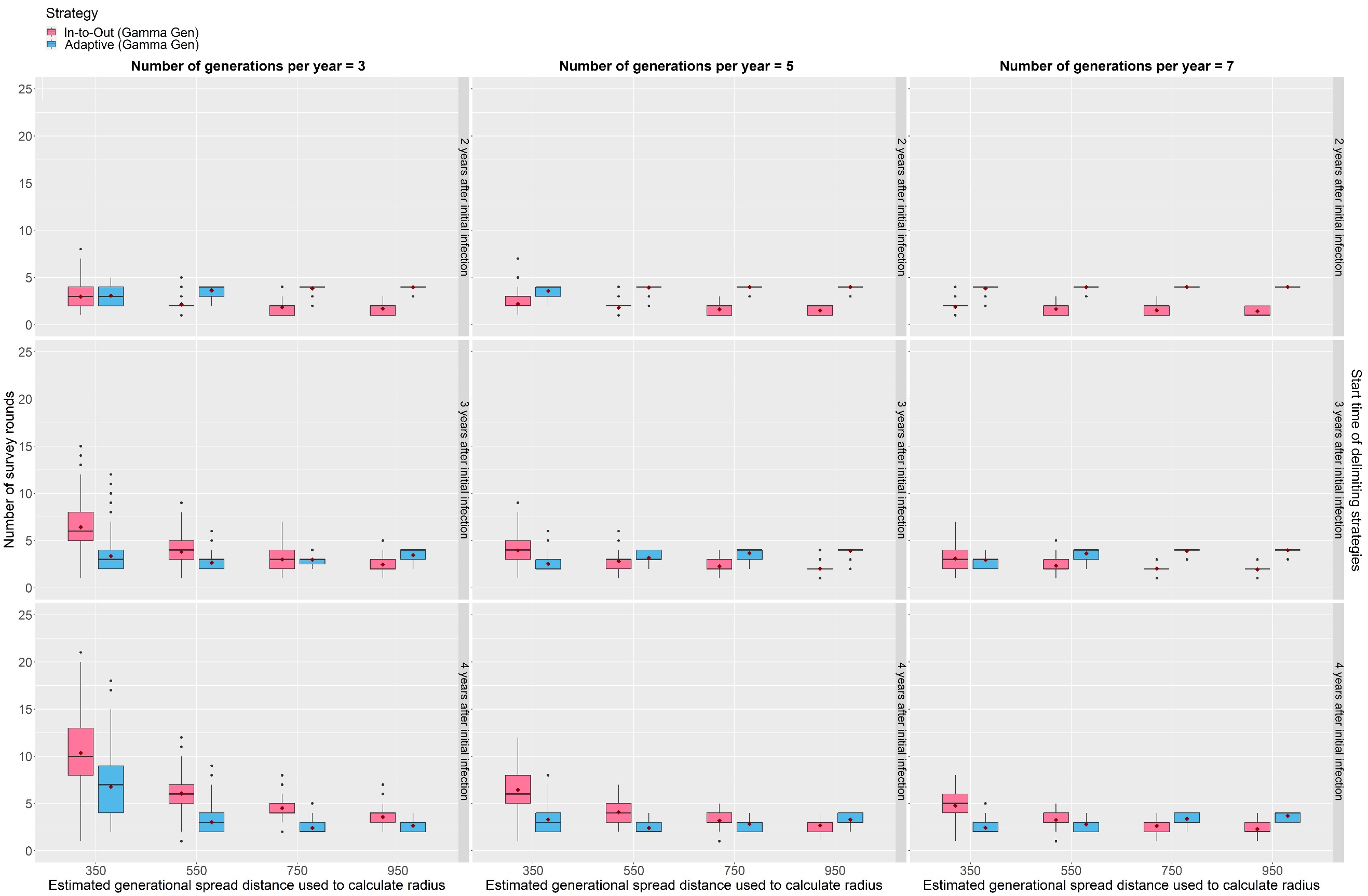


Figure S14: The change in the number of survey rounds the In-to-Out (Gamma Gen) and Adaptive (Gamma Gen) strategies took to delimit the potential infested zone on a random host landscape with varying inspector-estimated generational spread distances, mean number of generations per year and duration of pest spread. For each realization, both delimiting strategies started from the same randomly selected symptomatic individual, and a 1-year asymptomatic period was included. Method sensitivity was kept constant at 0.5. Boxplots were obtained from 500 iterations. Mean values are indicated with a dark red diamond.


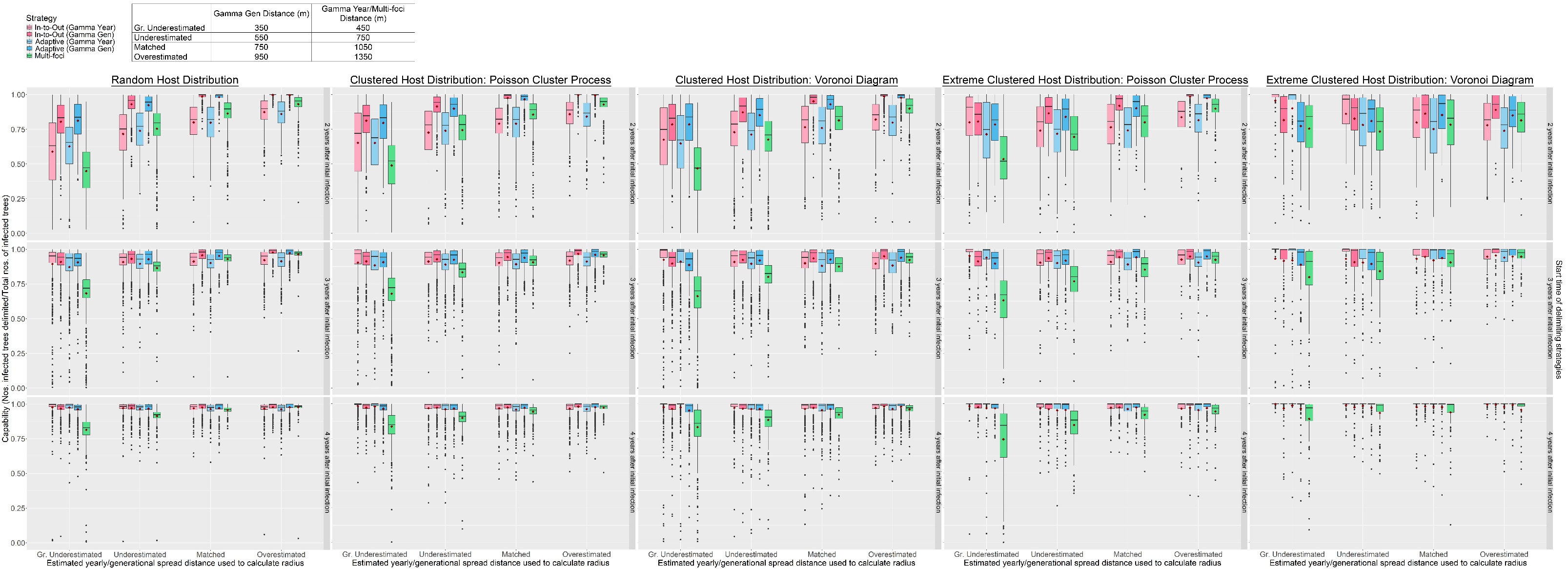


Figure S15: Change in the Capability scores of the delimiting strategies on various host landscape types in Scenario 5. For each realization, all delimiting strategies started from the same randomly selected symptomatic individual, and a 1-year asymptomatic period was included. Method sensitivity was kept constant at 0.5. Boxplots were obtained from 500 iterations except for the Extreme Clustered Host Distribution columns where boxplots were obtained from 150 iterations. Mean values are indicated with a dark red diamond.


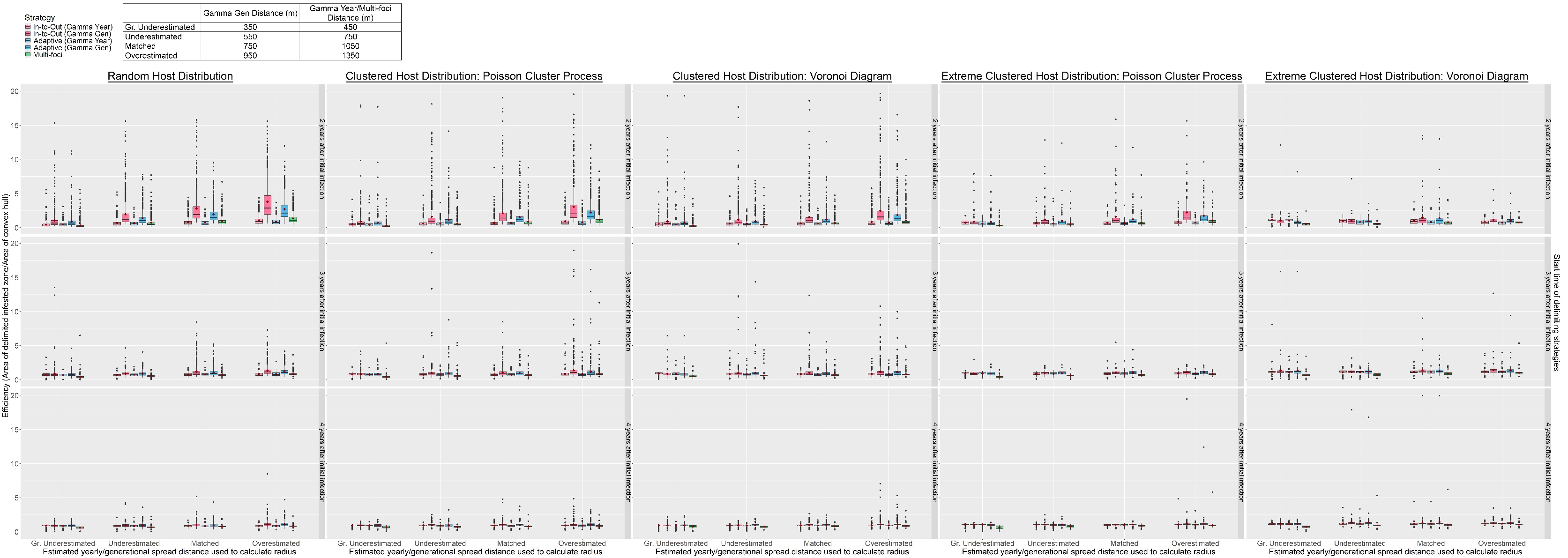


Figure S16: The change in Efficiency scores of the delimiting strategies on various host landscape types in Scenario 5. For each realization, all delimiting strategies started from the same randomly selected symptomatic individual, and a 1-year asymptomatic period was included. Method sensitivity was kept constant at 0.5. Boxplots were obtained from 500 iterations except for the Extreme Clustered Host Distribution columns where boxplots were obtained from 150 iterations. Mean values are indicated with a dark red diamond.


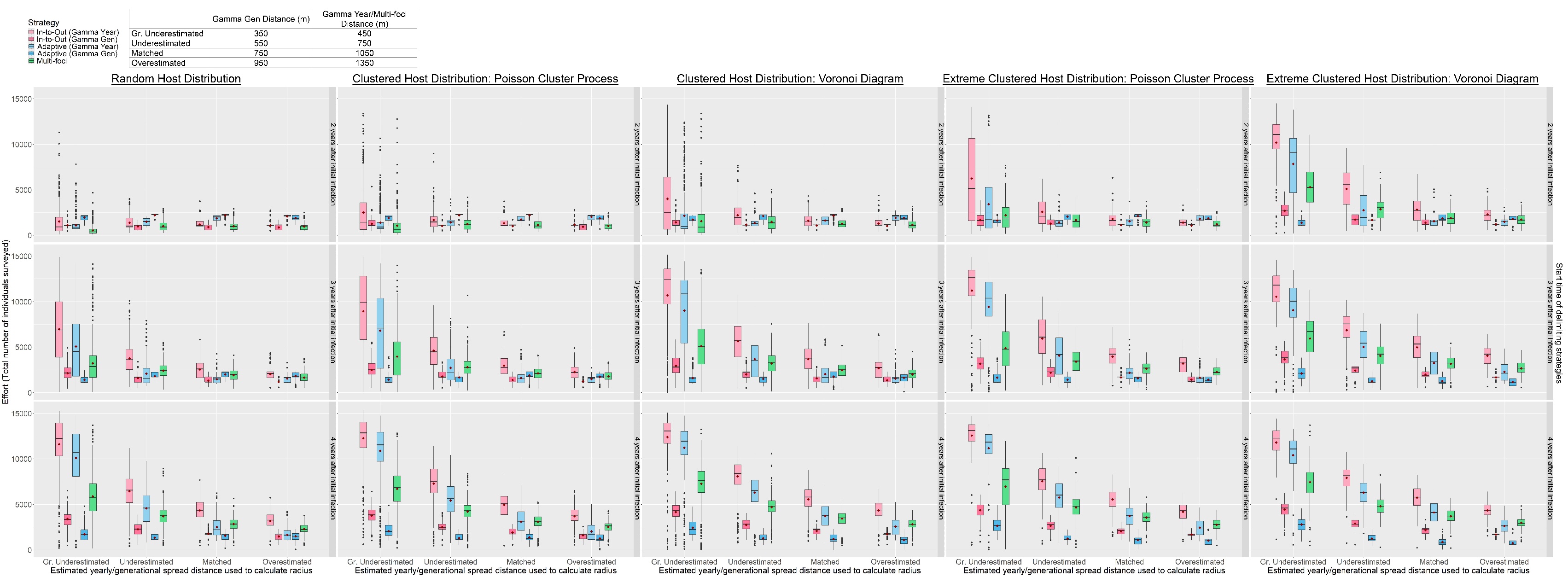


Figure S17: The change in Effort scores of the delimiting strategies on various host landscape types in Scenario 5. For each realization, all delimiting strategies started from the same randomly selected symptomatic individual, and a 1-year asymptomatic period was included. Method sensitivity was kept constant at 0.5. Boxplots were obtained from 500 iterations except for the Extreme Clustered Host Distribution columns where boxplots were obtained from 150 iterations. Mean values are indicated with a dark red diamond.


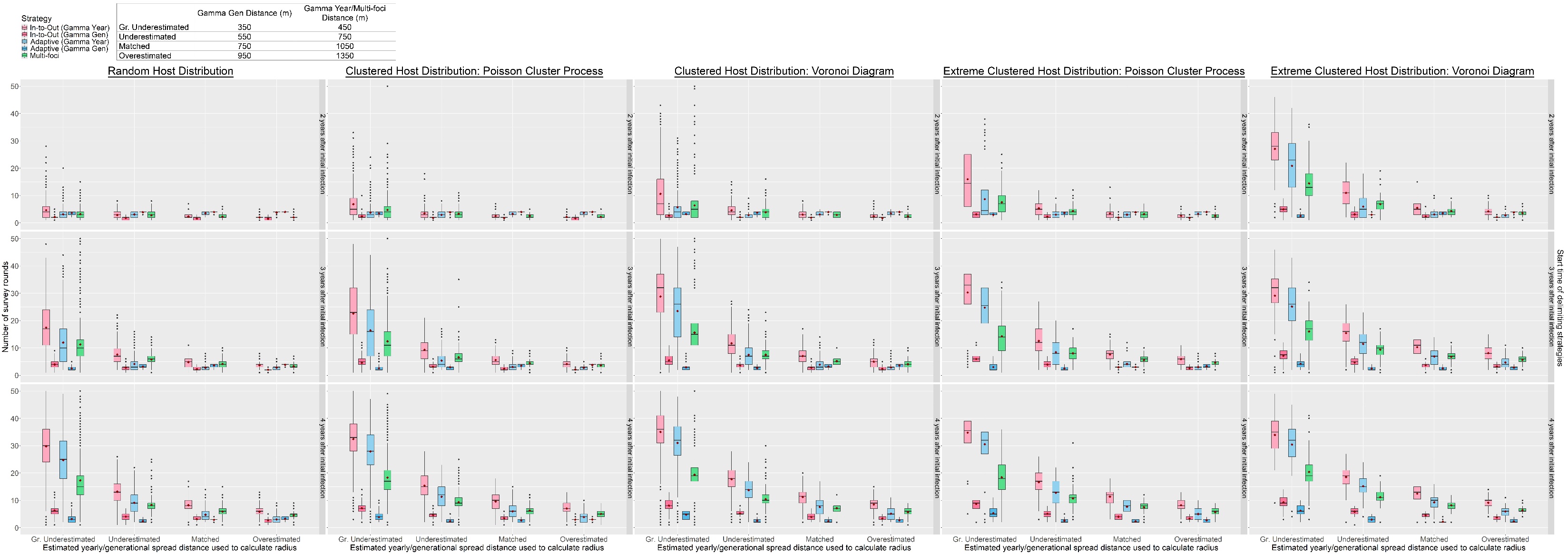


Figure S18: The change in the number of survey rounds each of the delimiting strategies took to delimit the potential infested zone on various host landscape types in Scenario 5. For each realization, all delimiting strategies started from the same randomly selected symptomatic individual, and a 1-year asymptomatic period was included. Method sensitivity was kept constant at 0.5. Boxplots were obtained from 500 iterations except for the Extreme Clustered Host Distribution columns where boxplots were obtained from 150 iterations.


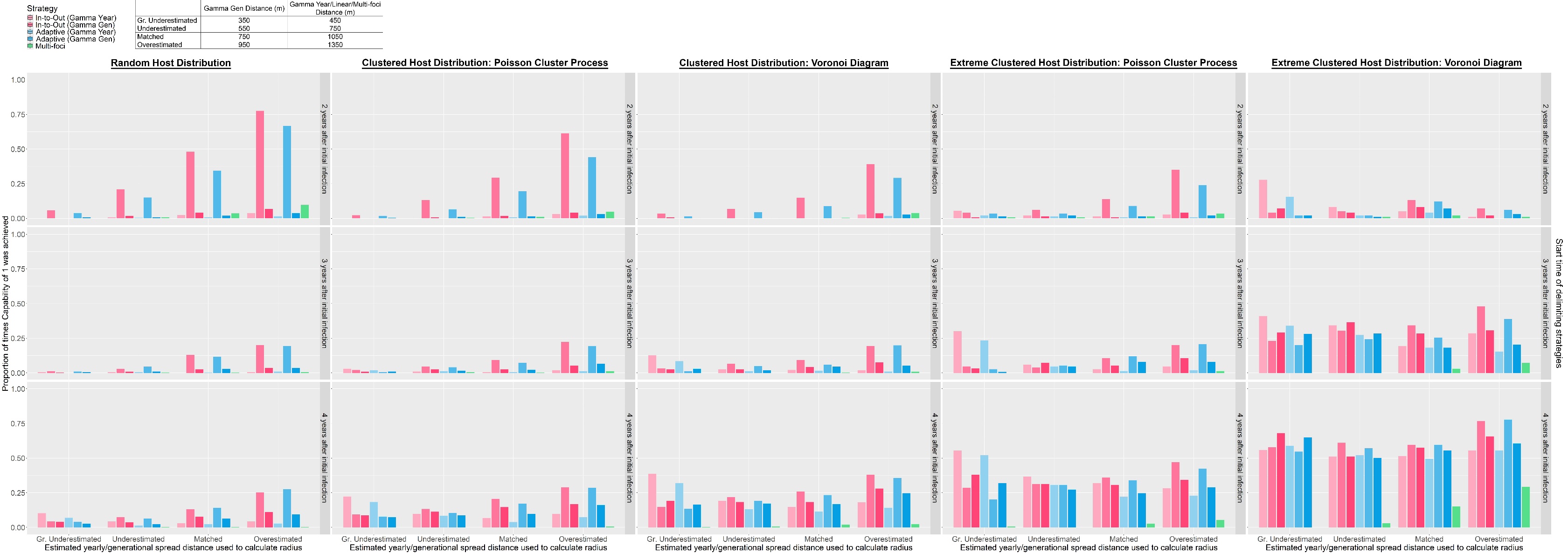


Figure S19: Change in the proportion of times perfect Capability was achieved by each of the delimiting strategies on various host landscape types with the inspector-estimated spread distances and duration of pest spread. For each realization, all delimiting strategies started from the same randomly selected symptomatic individual, and a 1-year asymptomatic period was included. Method Sensitivity was kept constant at 0.5. Each bar was obtained from 500 iterations.


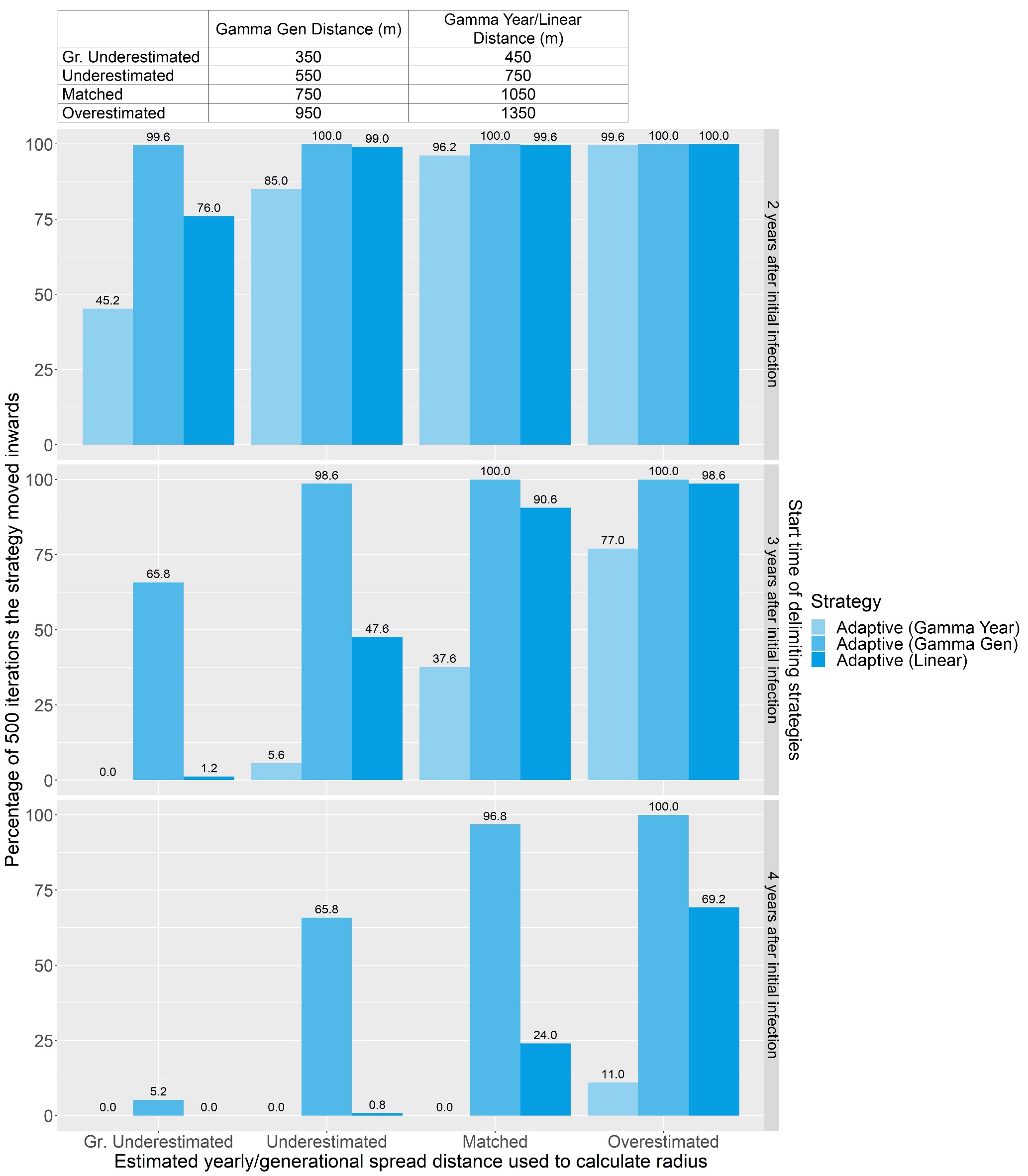


Figure S20: Bar graph showing how frequently each version of the Adaptive strategy correctly estimated the distance of epidemic spread and moved inwards on a random host landscape and when Method Sensitivity = 0.5.


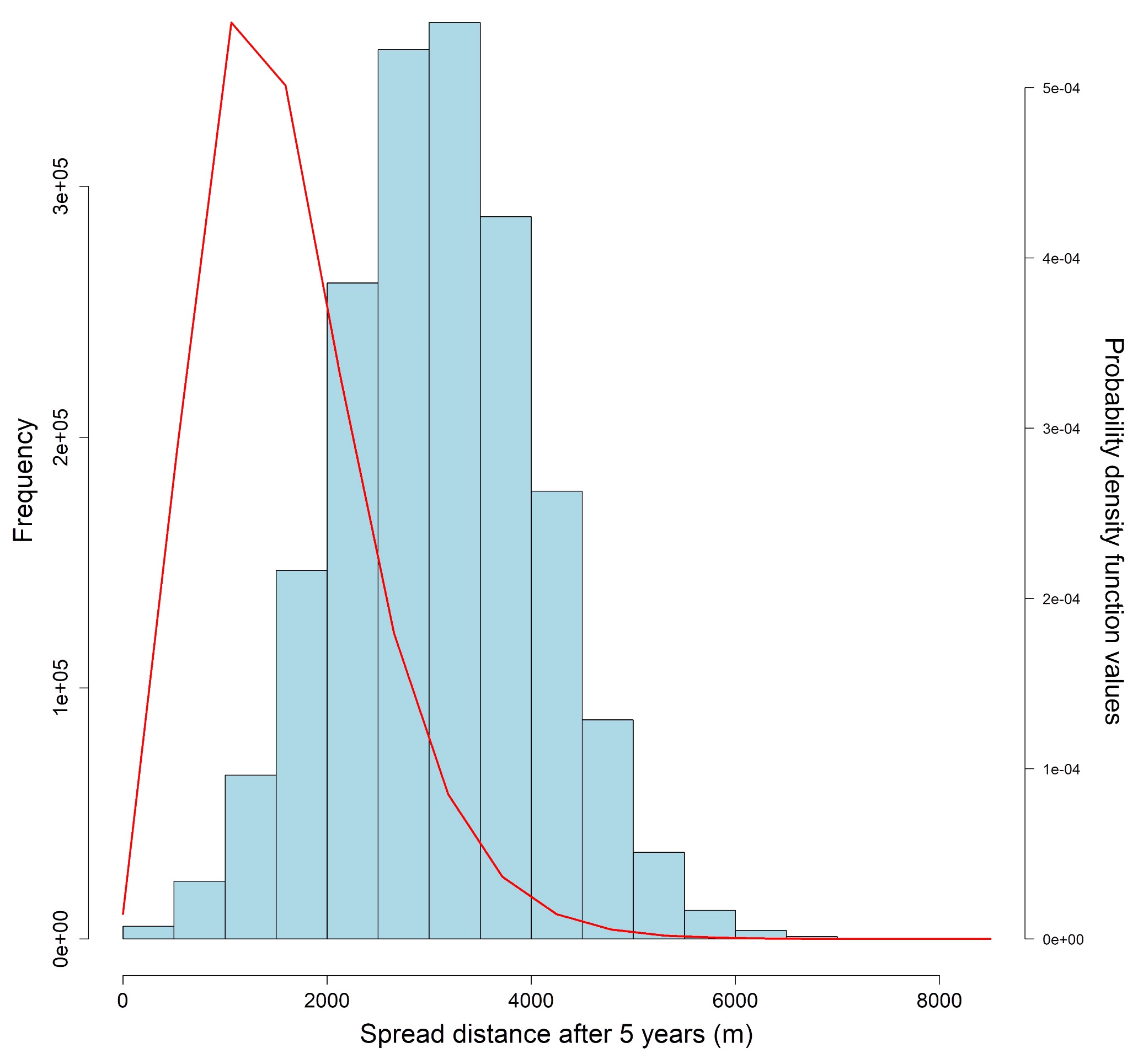


Figure S21: Histogram of the spread distances after five years and the probability density function values of a gamma distribution with a shape parameter value of 5 and a rate parameter value calculated with $-\frac{\ln(1-0.95)}{1056.911}$ .


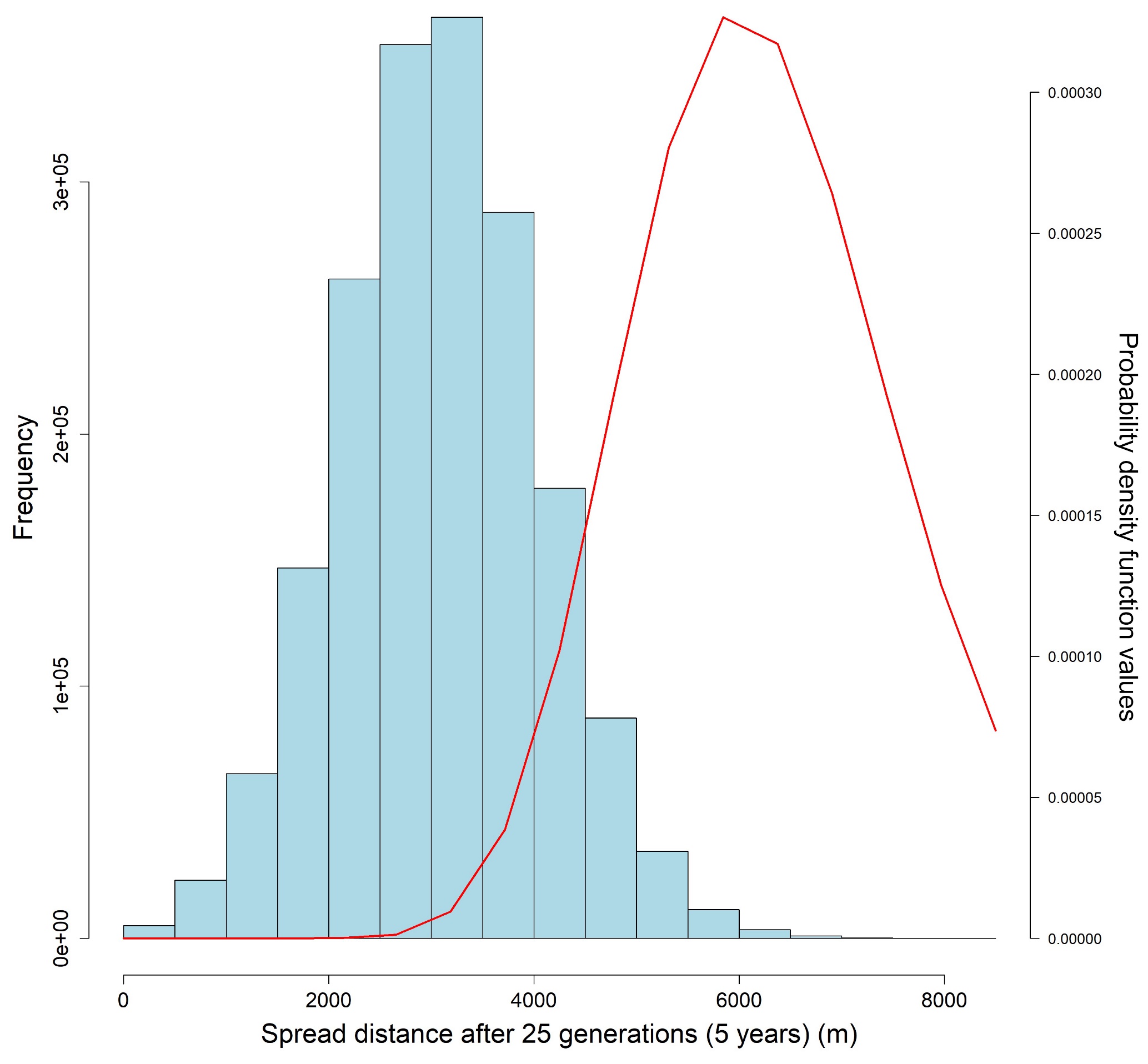


Figure S22: Histogram of the spread distances after twenty-five generations (five years) and the probability density function values of a gamma distribution with a shape parameter value of 25 and a rate parameter value calculated with $-\frac{\ln(1-0.95)}{737.69}$.


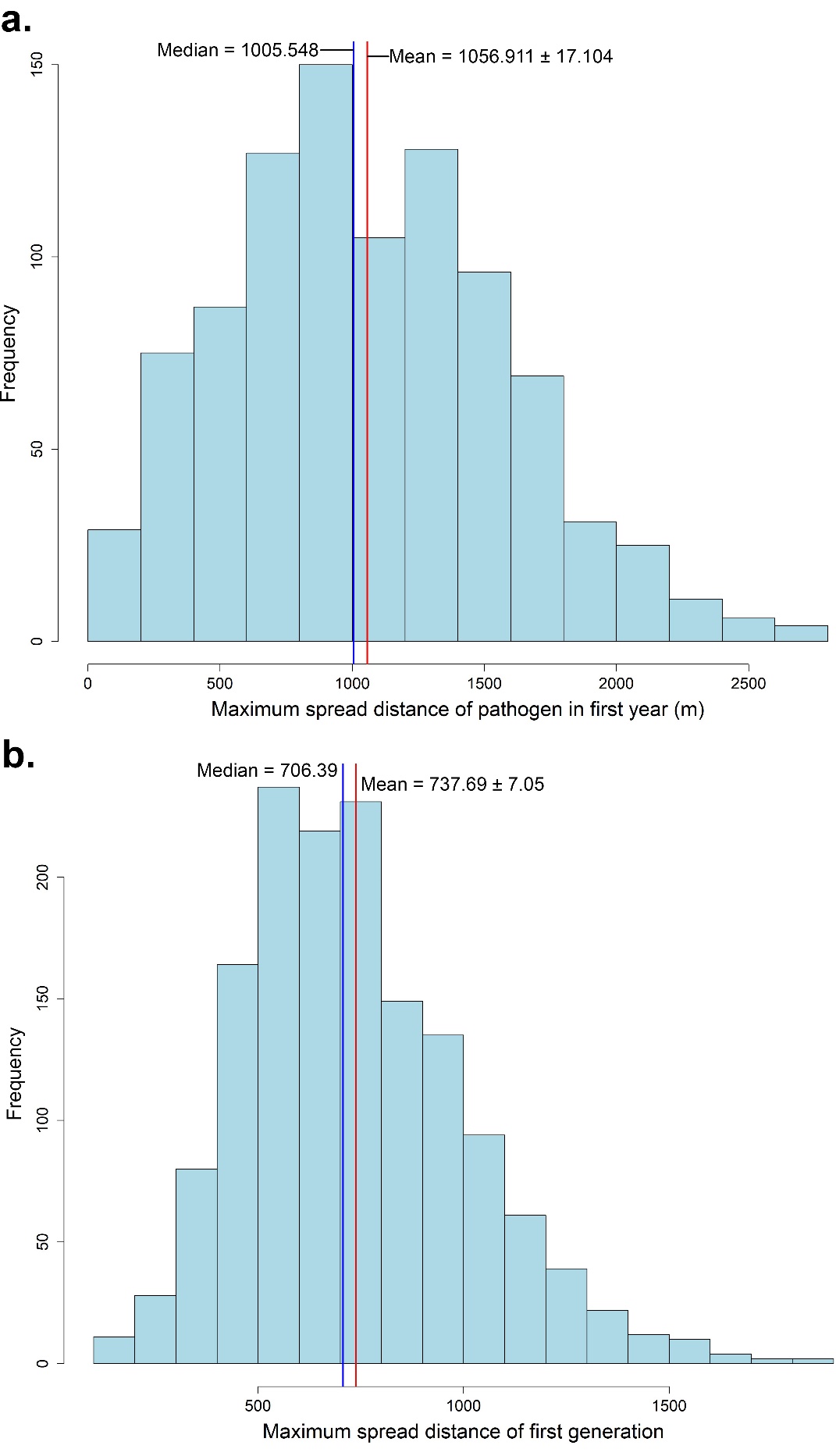


Figure S23: Histogram of the spread distances after the first year (a) and the first generation (b).


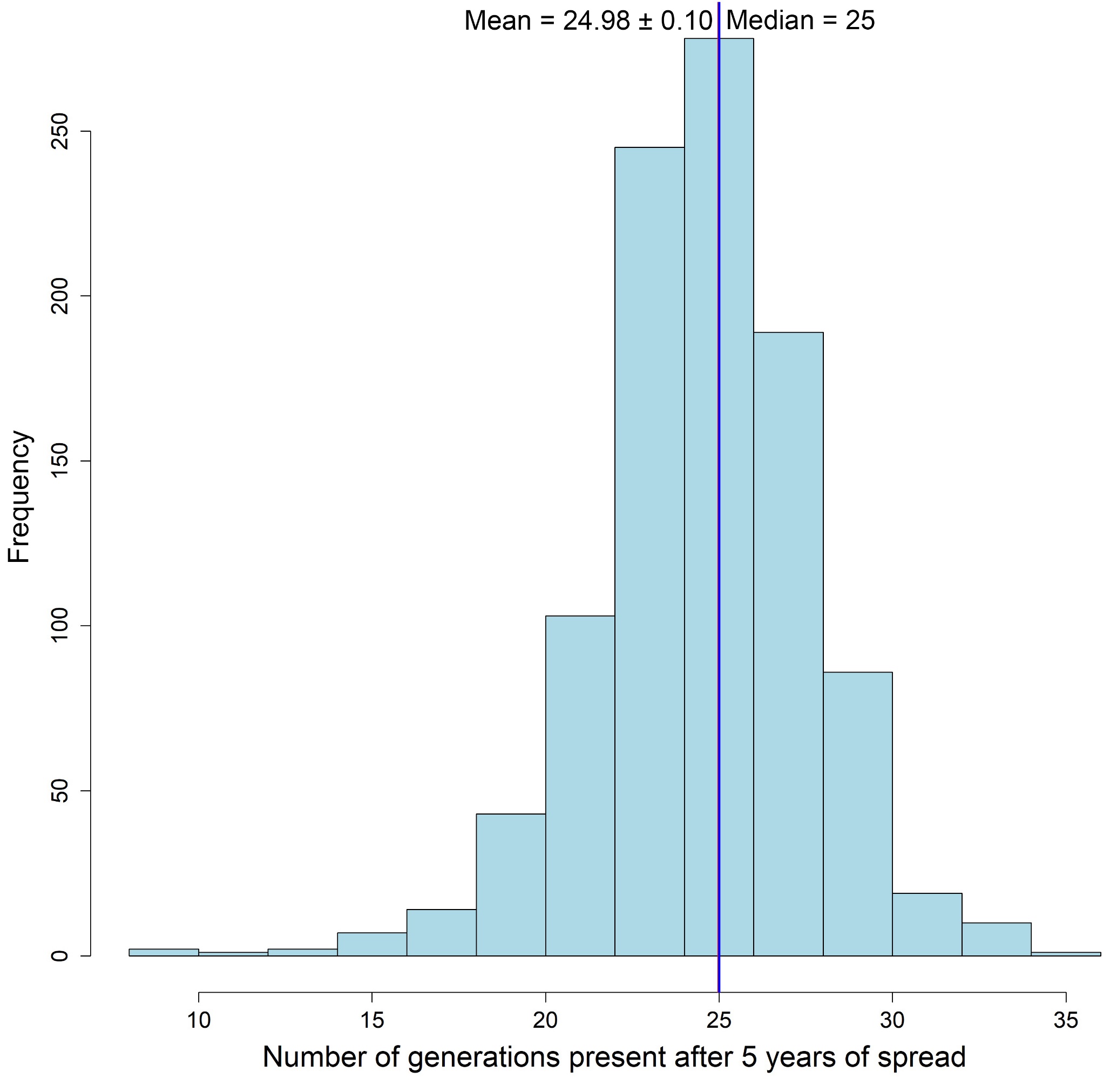


Figure S24: Histogram of the number of generations present in the epidemic after 5 years of spread.


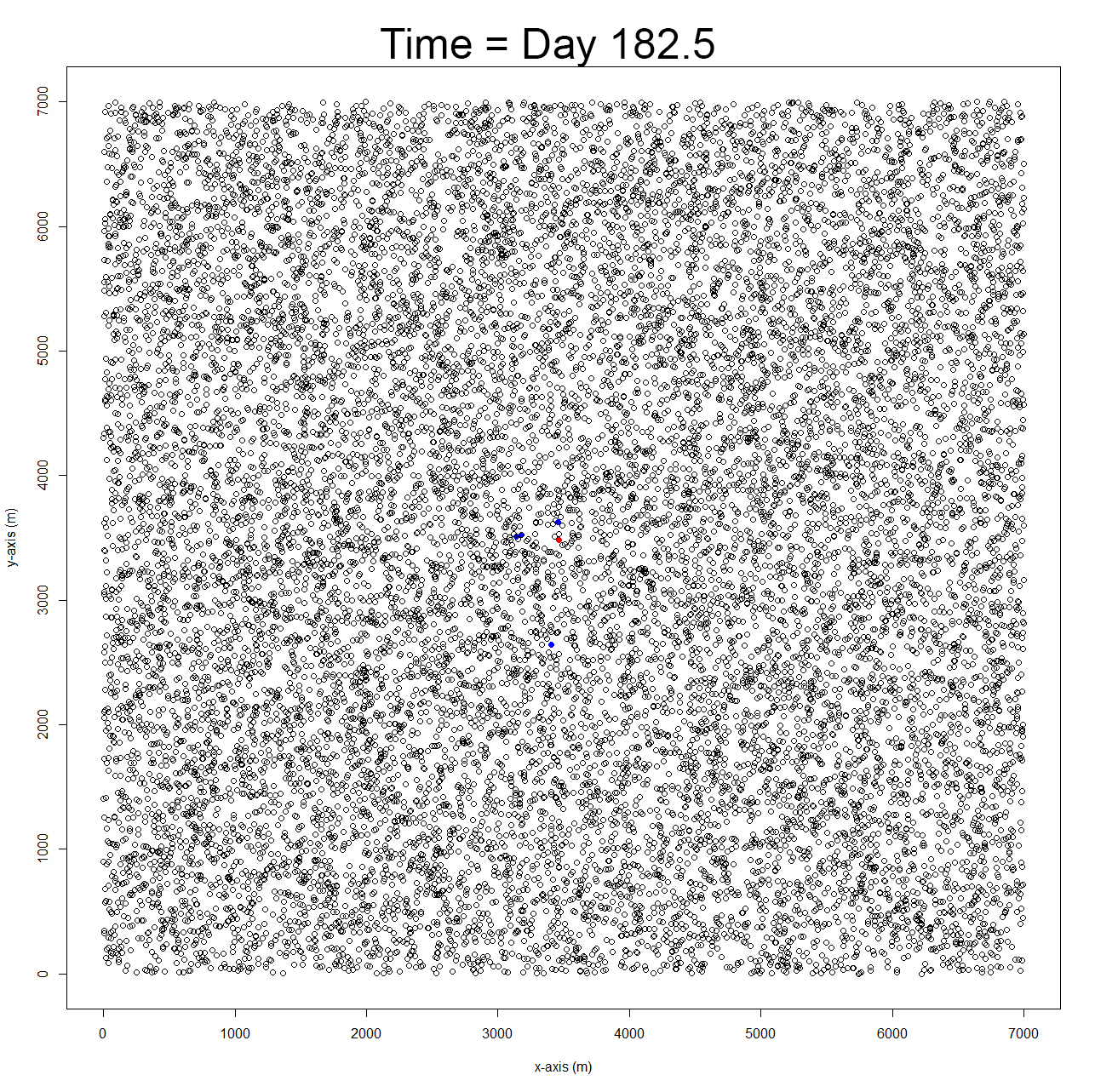


Figure SA1: Animated GIF showing the spread of the simulated pest in a random host landscape. Black circles indicate susceptible individuals, pink dots represent symptomatic individuals, blue dots represent asymptomatic individuals, and the red dot represents the first infected individual or the origin of the epidemic.


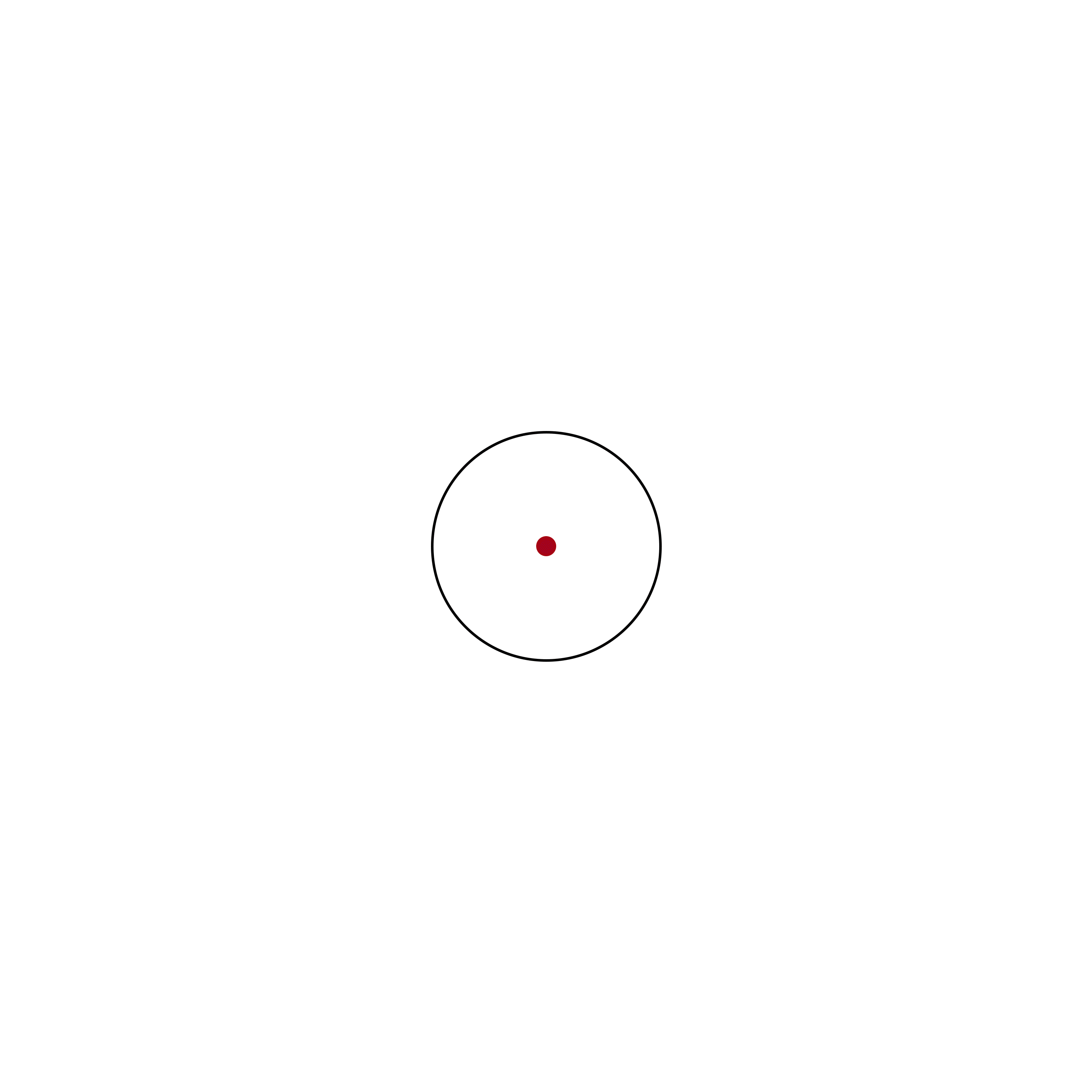


Figure SA2: Animated GIF showing how the In-to-Out strategy works. Colored dots represent detections of infected individuals, while the corresponding-colored bands represent the surveys conducted following these detections. The final purple circle represents the boundary of the delimited potential infested zone.


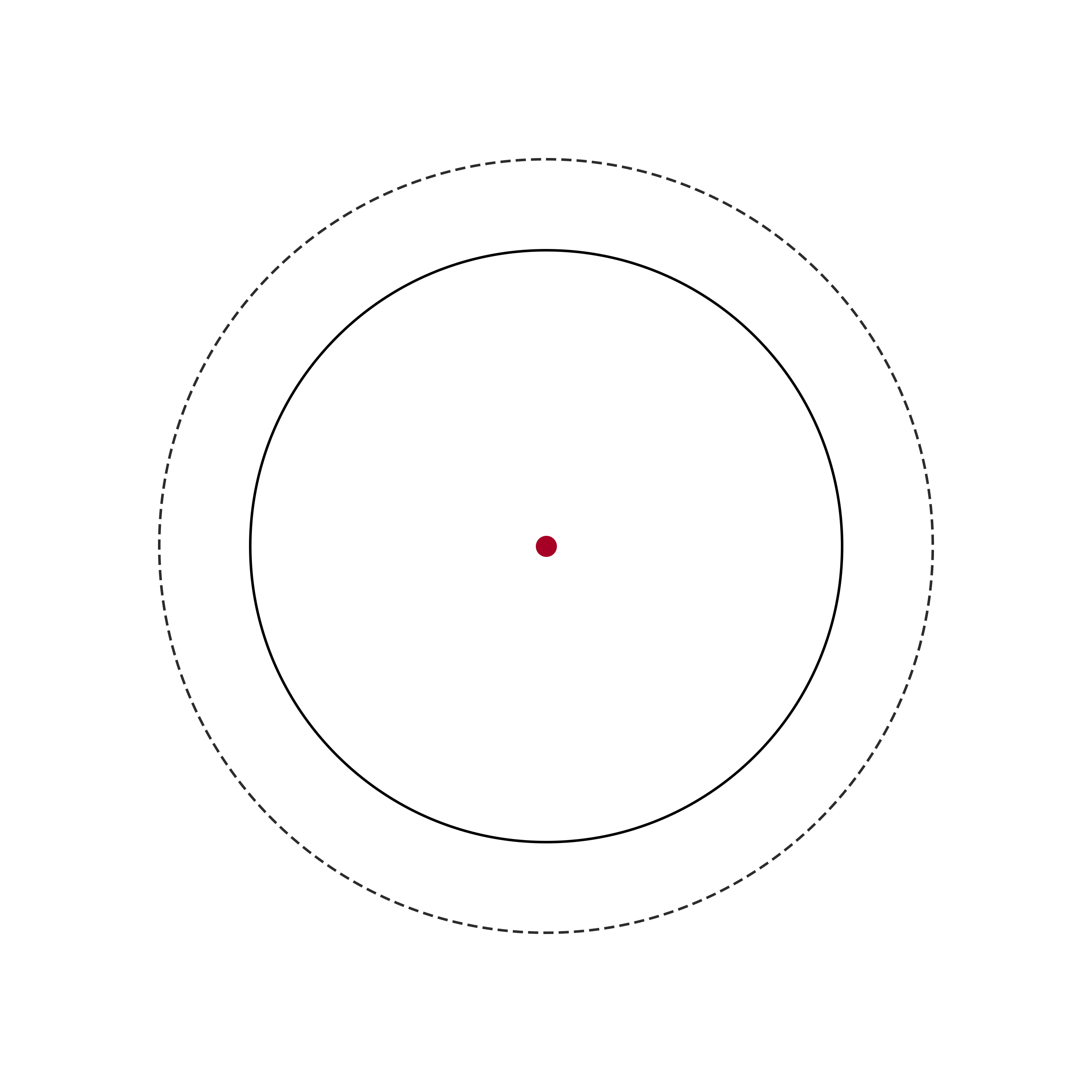


Figure SA3: Animated GIF showing how the Adaptive strategy works when a detection is made in the preliminary survey. Colored dots represent detections of infected individuals, while the corresponding-colored bands represent the surveys conducted following these detections. The final purple circle represents the boundary of the delimited potential infested zone.


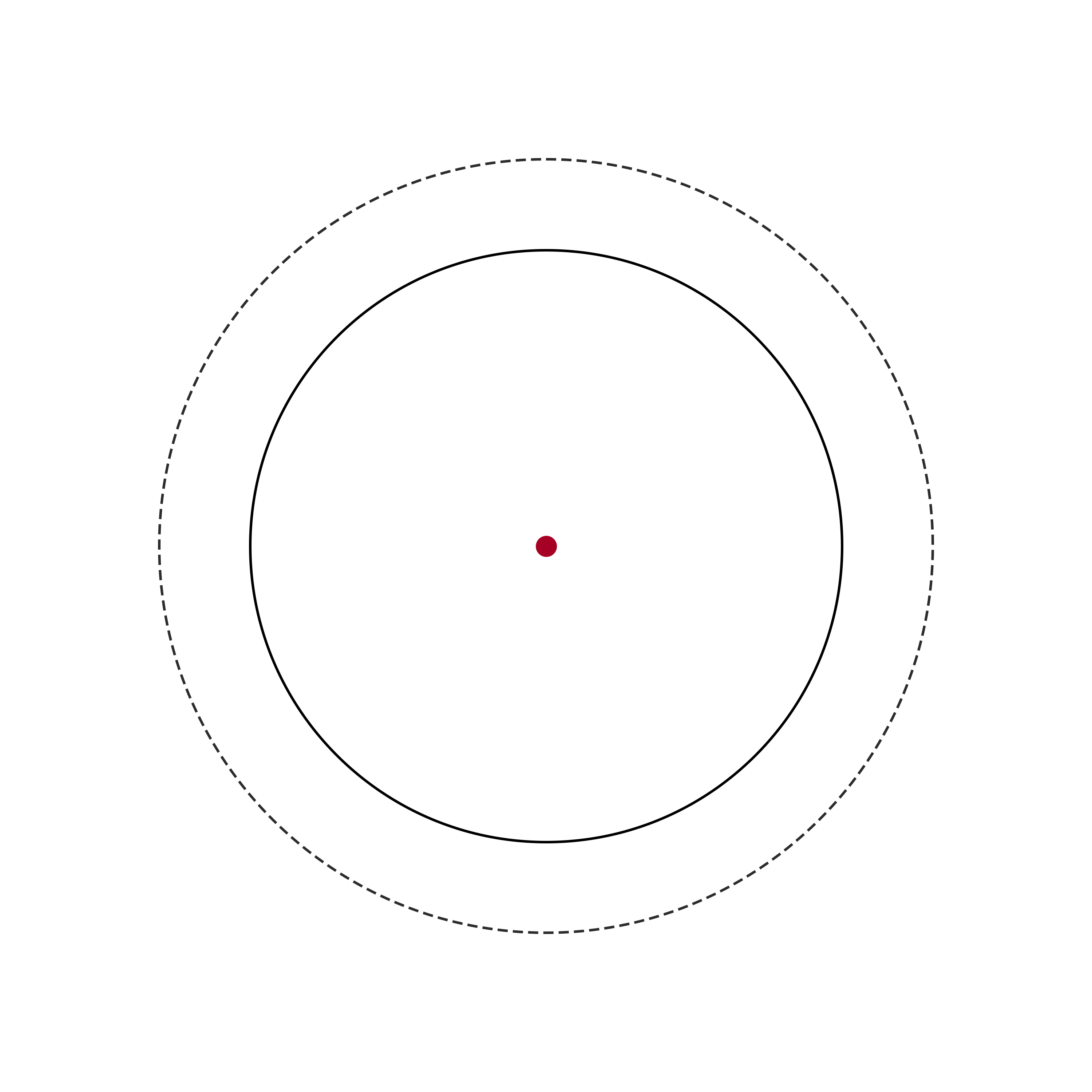


Figure SA4: Animated GIF showing how the Adaptive strategy works when no detection is made in the preliminary survey. Colored dots represent detections of infected individuals, while the corresponding-colored bands represent the surveys conducted following these detections. The final purple circle represents the boundary of the delimited potential infested zone.


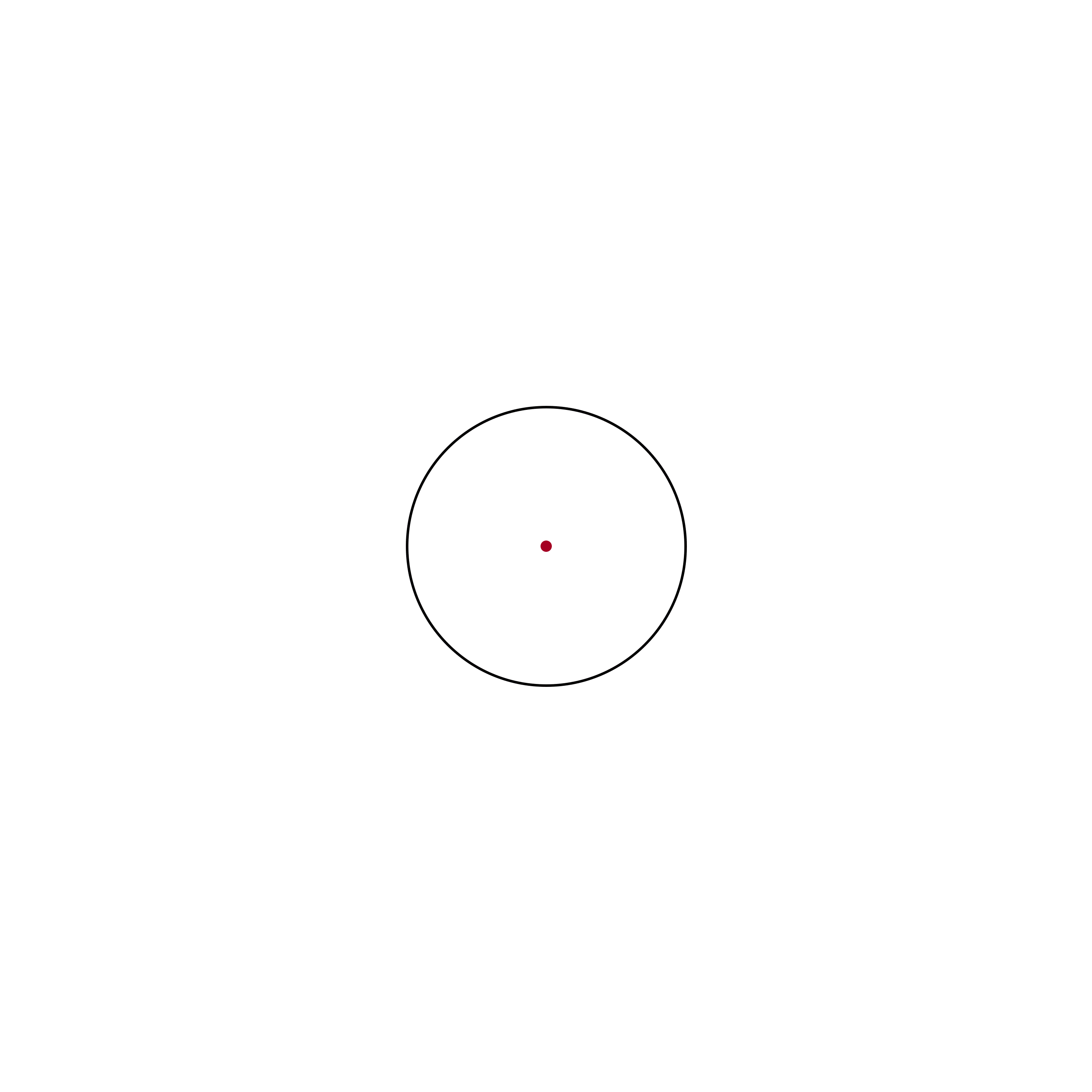


Figure SA5: Animated GIF showing how the Multi-foci strategy works. Colored dots represent detections of infected individuals, while the corresponding-colored bands represent the surveys conducted following these detections. The final purple circle represents the boundary of the delimited potential infested zone.


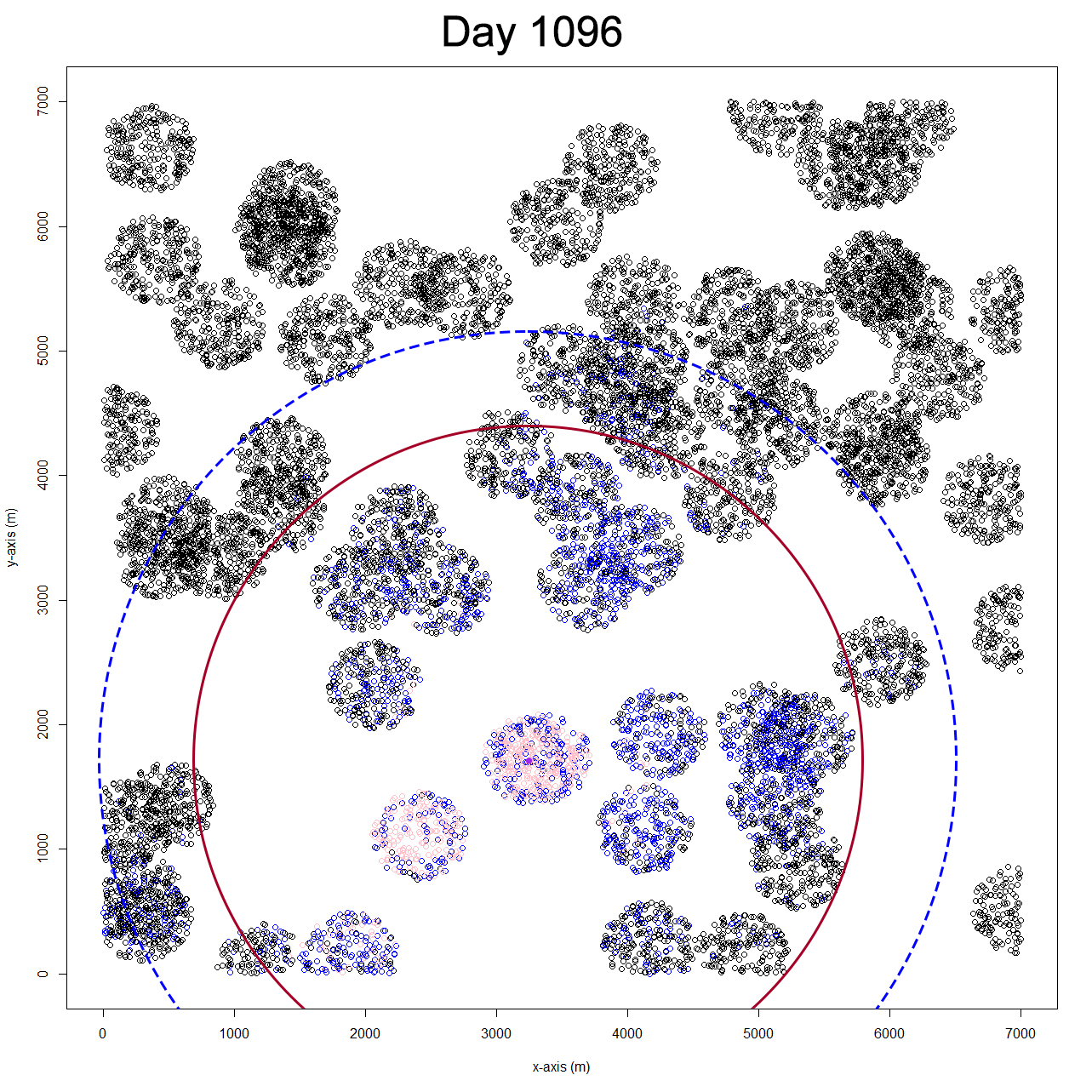


Figure SA6: Animated GIF showing a realization of the Adaptive (Gamma Gen) strategy on an Extreme Clustered Poisson Cluster Process host landscape. The purple dot represents the initial detection, pink circles represent symptomatic individuals, blue circles represent asymptomatic individuals, black circles represent susceptible individuals, and the green dot represents the subsequent detection. Large colored circles represent the boundaries of the survey bands. The blue dotted circle represents the outer boundary of the preliminary survey.


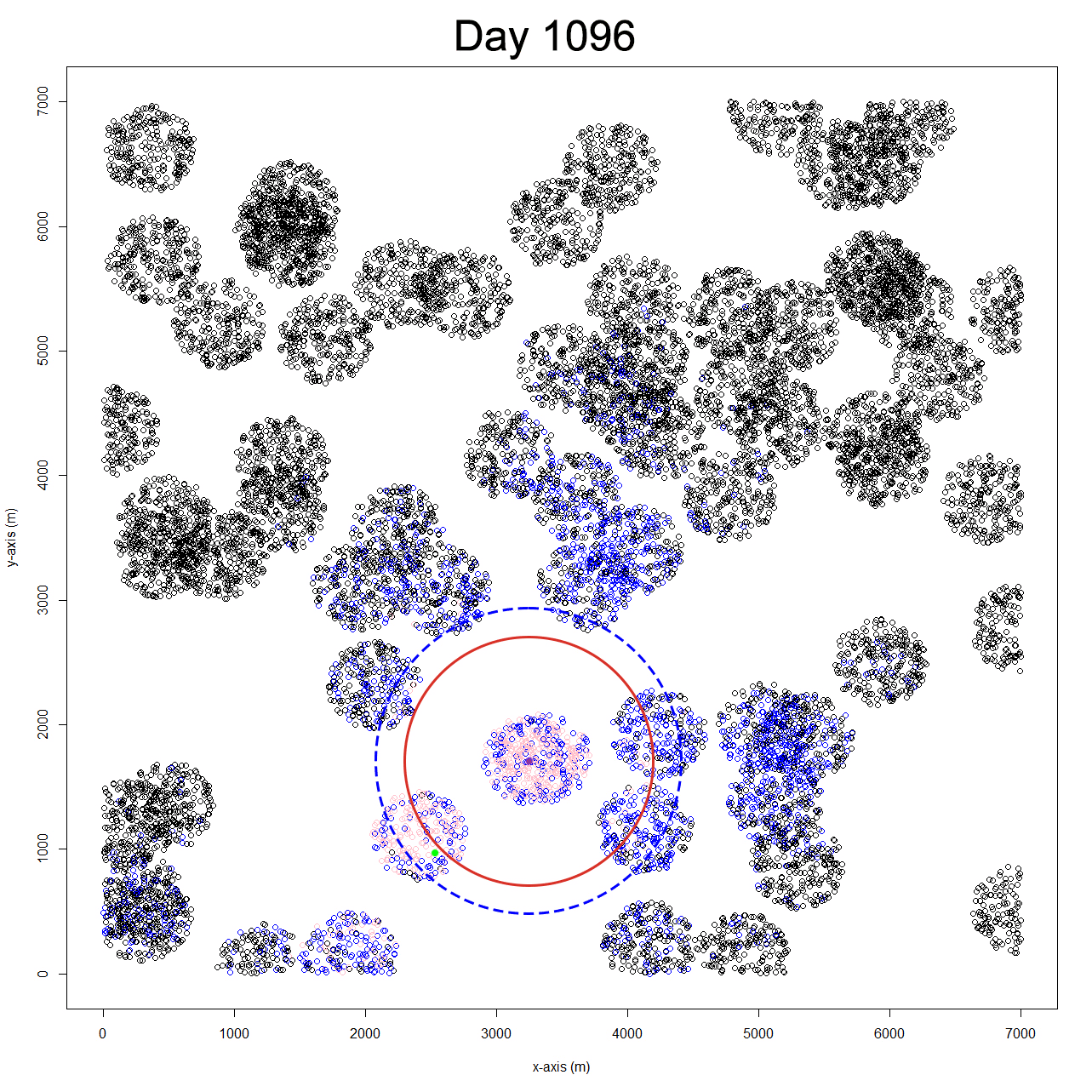


Figure SA7: Animated GIF showing a realization of the Adaptive (Gamma Year) strategy on the same Extreme Clustered Poisson Cluster Process host landscape as Fig. SA6. The purple dot represents the initial detection, pink circles represent symptomatic individuals, blue circles represent asymptomatic individuals, black circles represent susceptible individuals, and the green dot represents the subsequent detection. Large colored circles represent the boundaries of the survey bands. The blue dotted circle represents the outer boundary of the preliminary survey.


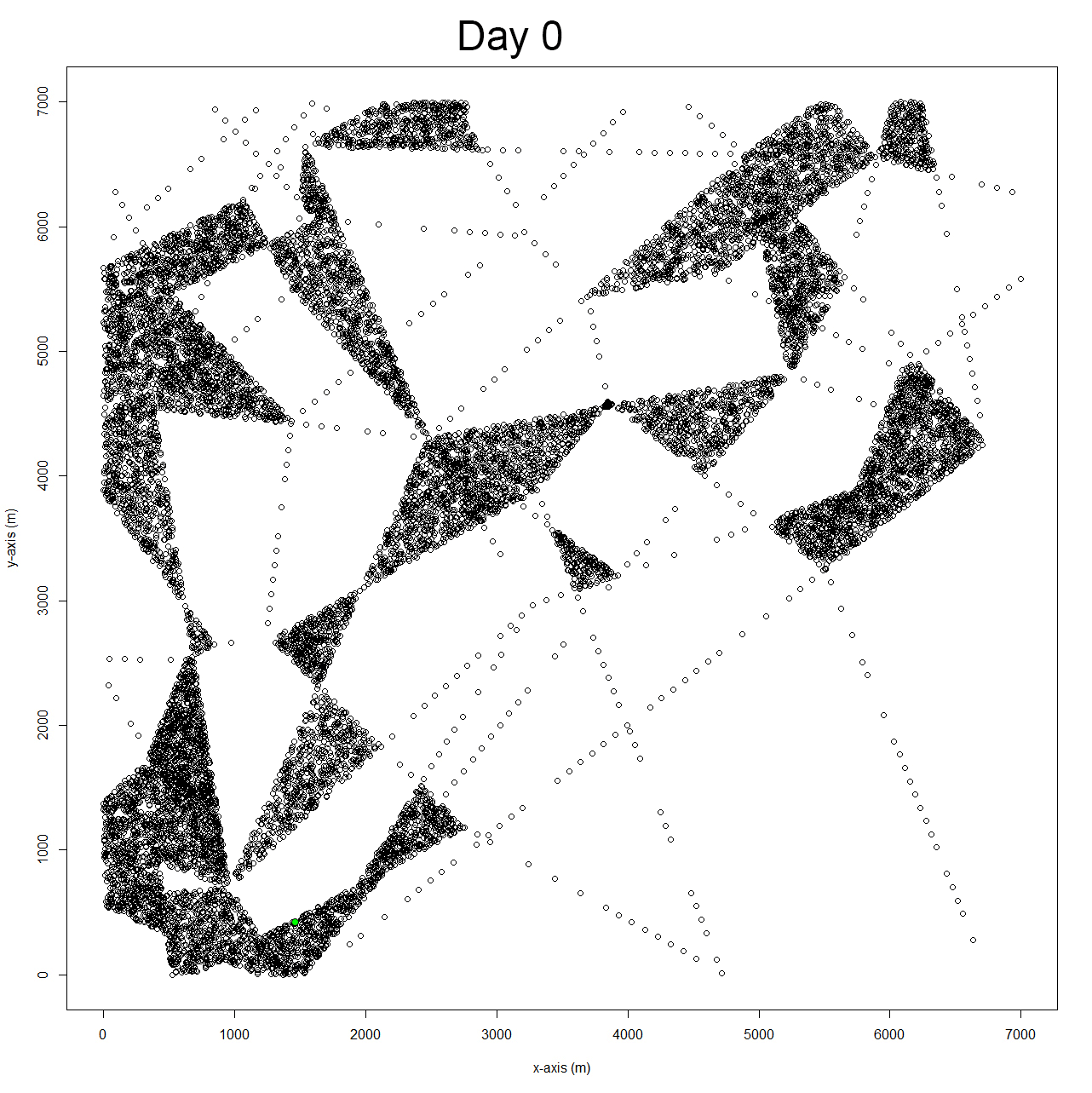


Figure SA8: Animated GIF showing a realization of the Individual Based Model and Multi-foci strategy on an Extreme Clustered Voronoi Diagram host landscape. The inspector-estimated (IE) annual spread distance of the Multi-foci strategy matched the true value of the simulated pest (1050 m/yr) and Method Sensitivity was 0.5. The purple dot represents the initial detection, pink dots represent symptomatic individuals, blue dots represent asymptomatic individuals, black circles represent susceptible individuals, the green dot represents the origin of the epidemic, red and orange dots represent the detections made during the survey rounds. Large multi-colored circles or globulus shapes represent the outer boundaries of survey rounds. The final shape with a green outline and purple filling represents the delimited potential infested zone.


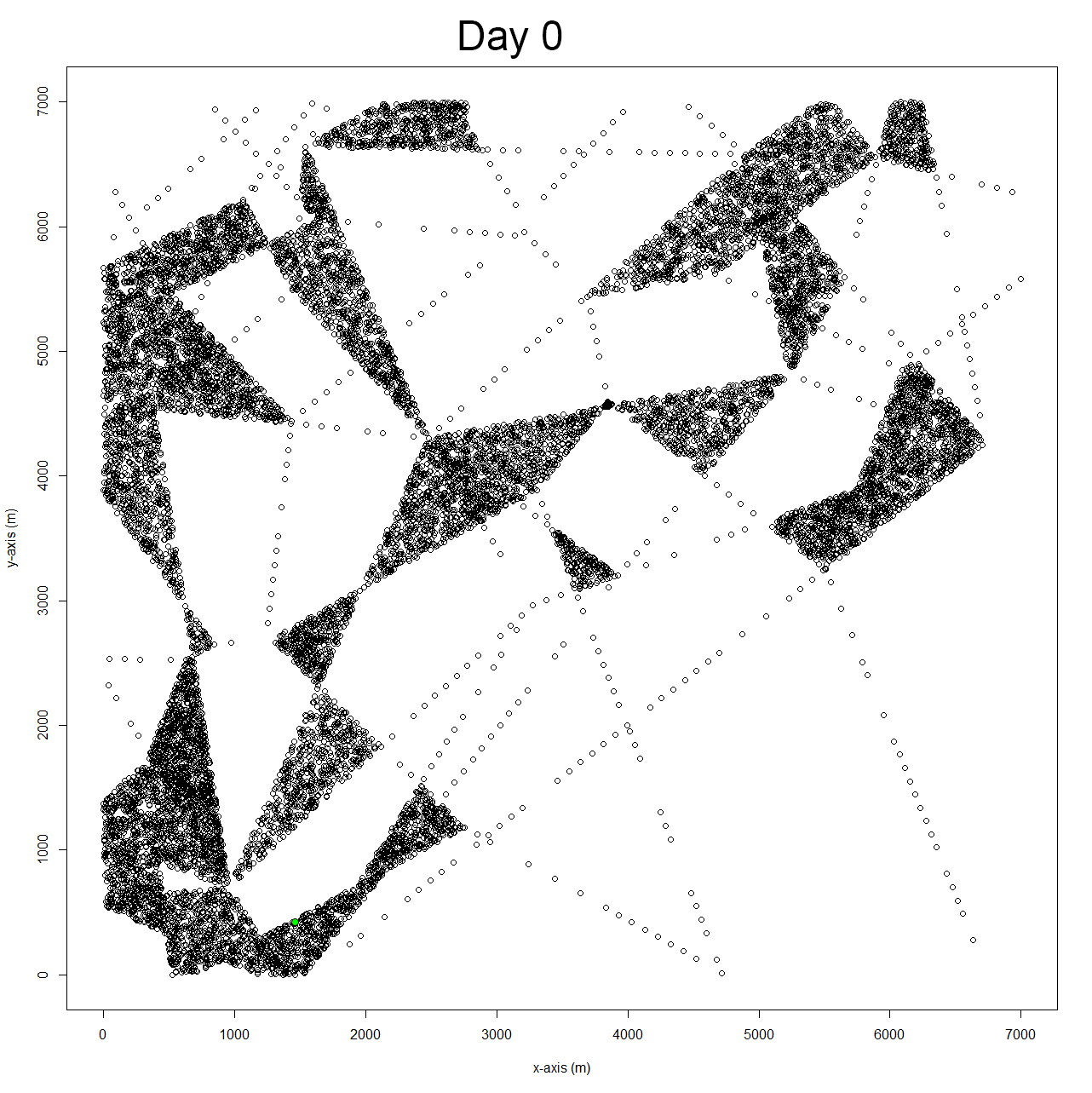


Figure SA9: Animated GIF showing a realization of the Adaptive (Gamma Gen) strategy on the same Extreme Clustered Voronoi Diagram host landscape, starting symptomatic host, and realization of the Individual Based Model as in Fig. SA8. The inspector-estimated (IE) generational spread distance of the Adaptive (Gamma Gen) strategy matched the true value of the simulated pest (750 m/yr) and Method Sensitivity was 0.5. The purple dot represents the initial detection, pink dots represent symptomatic individuals, blue dots represent asymptomatic individuals, black circles represent susceptible individuals, the green dot represents the origin of the epidemic, red and orange dots represent the detections made during the survey rounds. Large multi-colored circles represent the boundaries of survey rounds, and the highlighted band shows the survey area for that round. The final shape with a green outline and purple filling represents the delimited potential infested zone.
