# Supplementary figures and images for: Assessing delimiting strategies to identify the infested zones of quarantine plant pests and diseases
Source: Sci Rep. 2025 Feb 15;15:5610. doi: 10.1038/s41598-025-90343-2 (PMC11829978; doi:10.1038/s41598-025-90343-2)

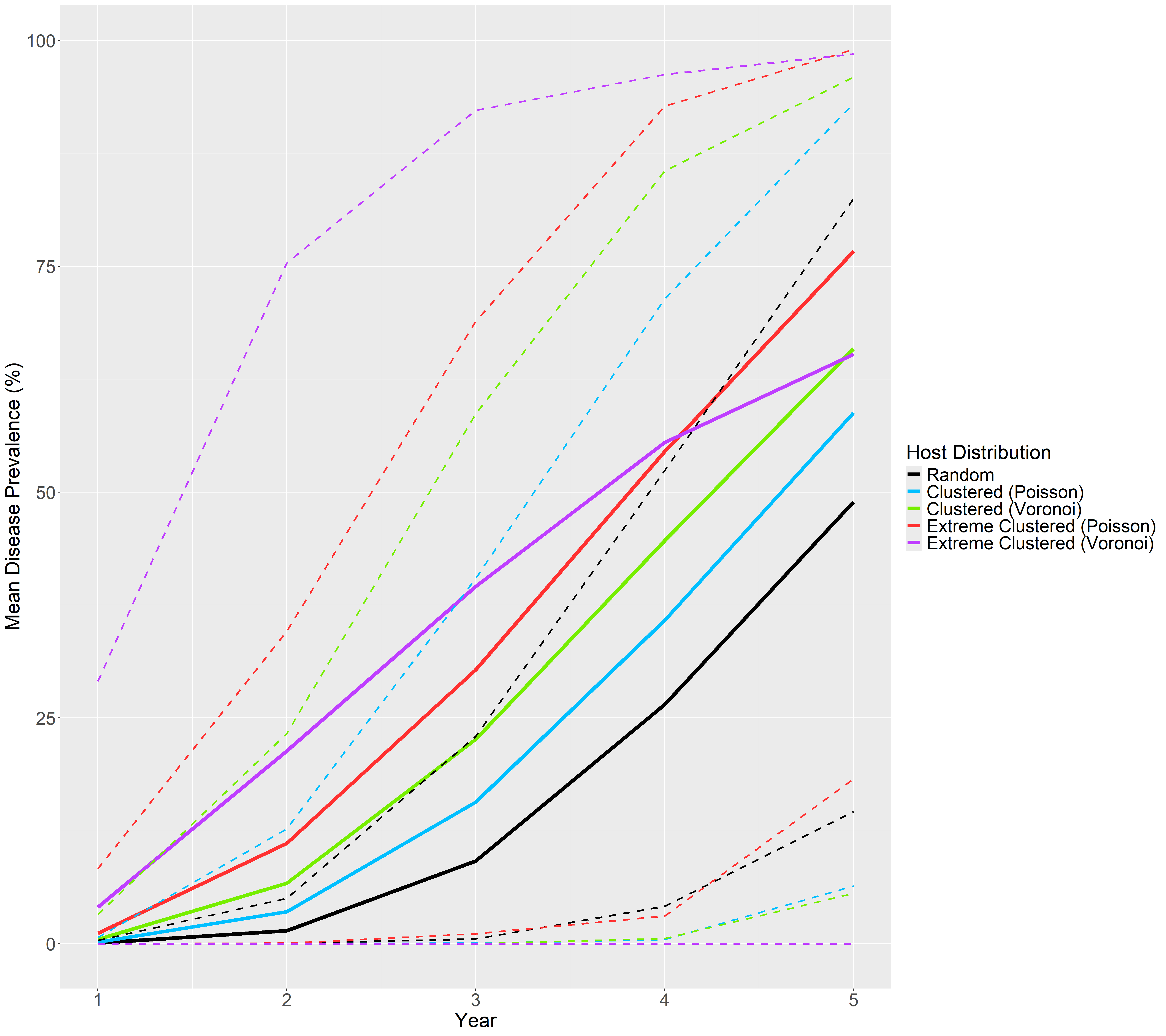

Supplement: Supplementary file 3 — Supplementary Material 3 [file 41598_2025_90343_MOESM3_ESM.png]

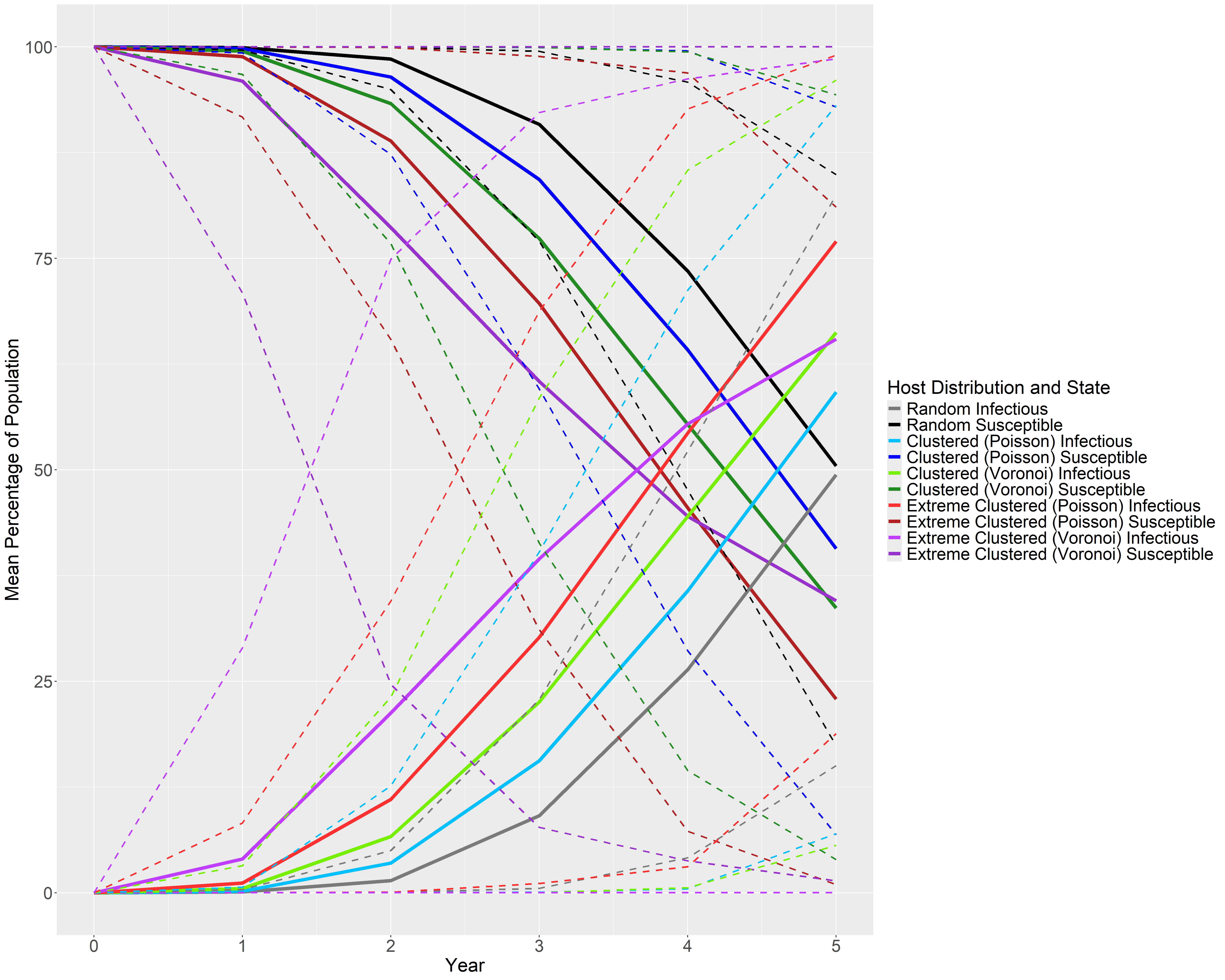

Supplement: Supplementary file 4 — Supplementary Material 4 [file 41598_2025_90343_MOESM4_ESM.png]

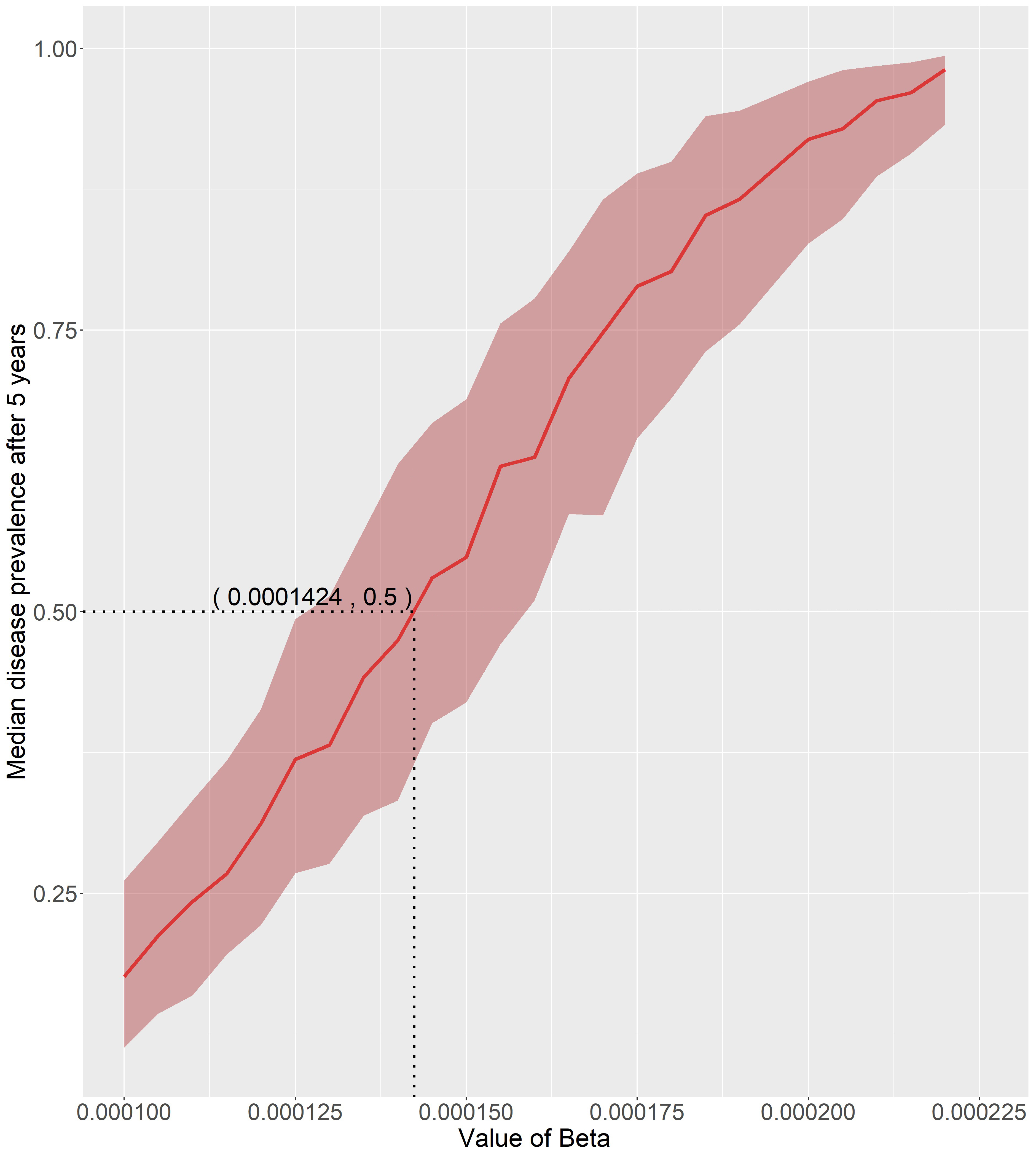

Supplement: Supplementary file 5 — Supplementary Material 5 [file 41598_2025_90343_MOESM5_ESM.jpg]

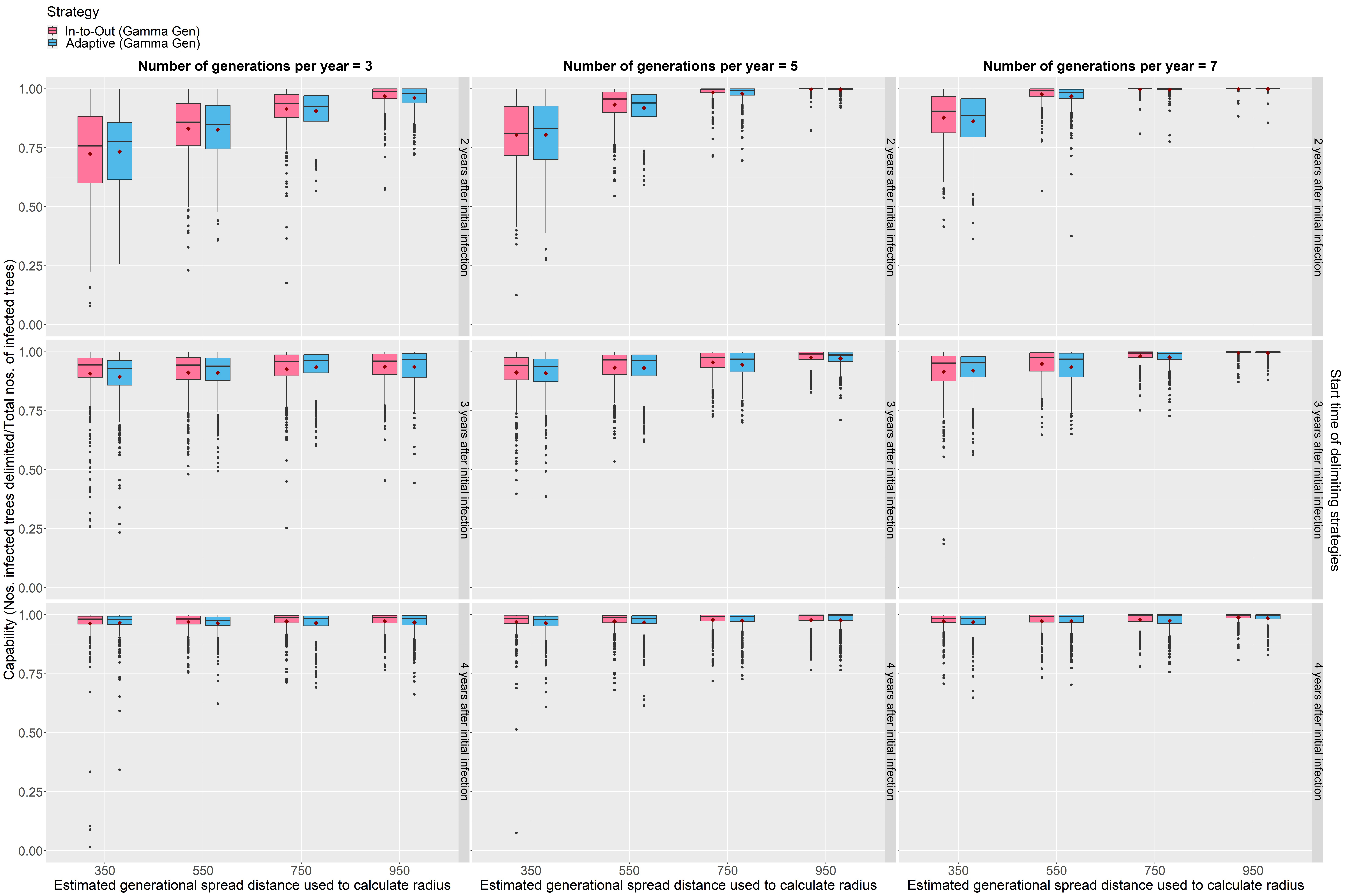

Supplement: Supplementary file 13 — Supplementary Material 13 [file 41598_2025_90343_MOESM13_ESM.jpg]

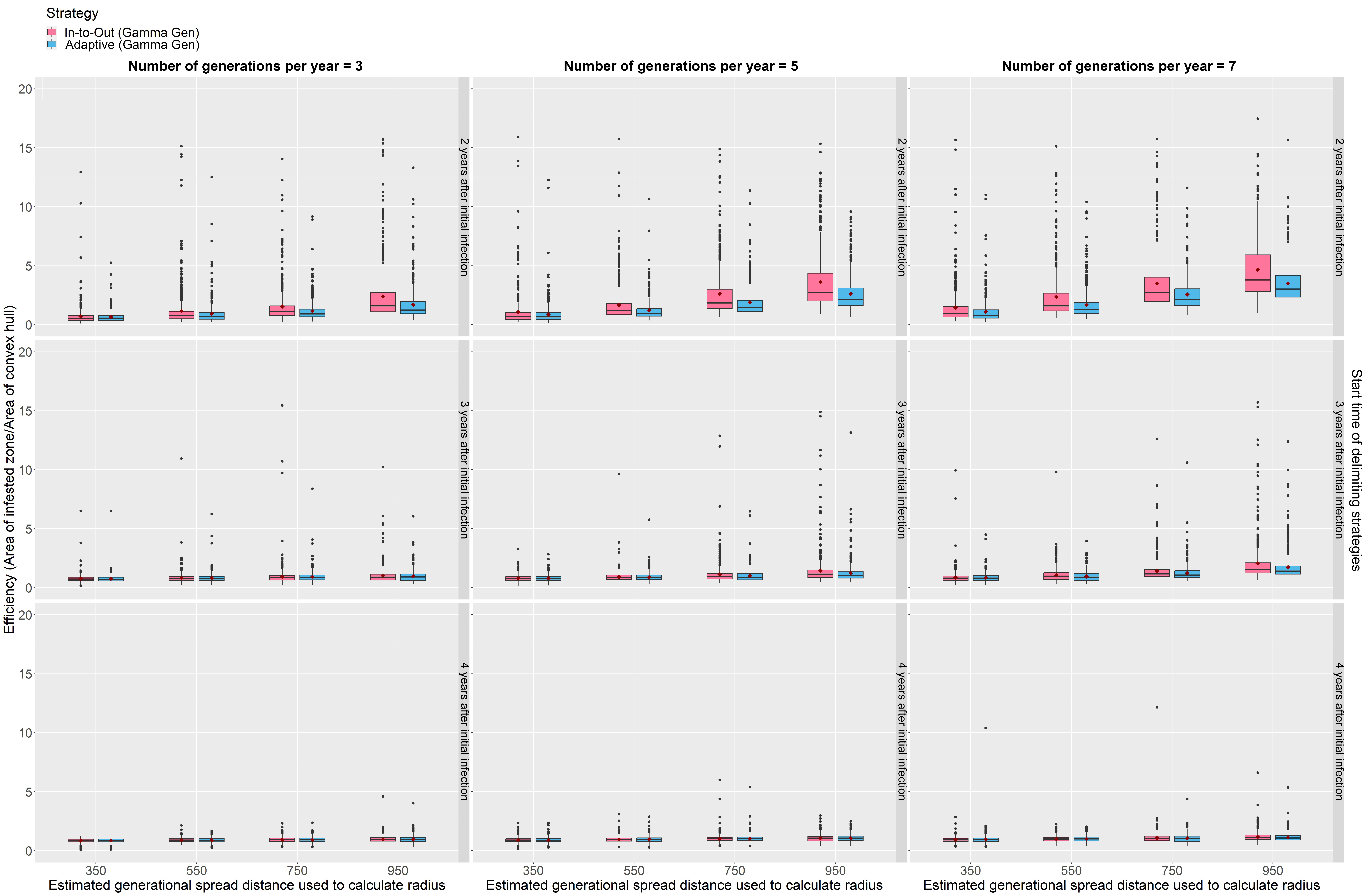

Supplement: Supplementary file 14 — Supplementary Material 14 [file 41598_2025_90343_MOESM14_ESM.jpg]

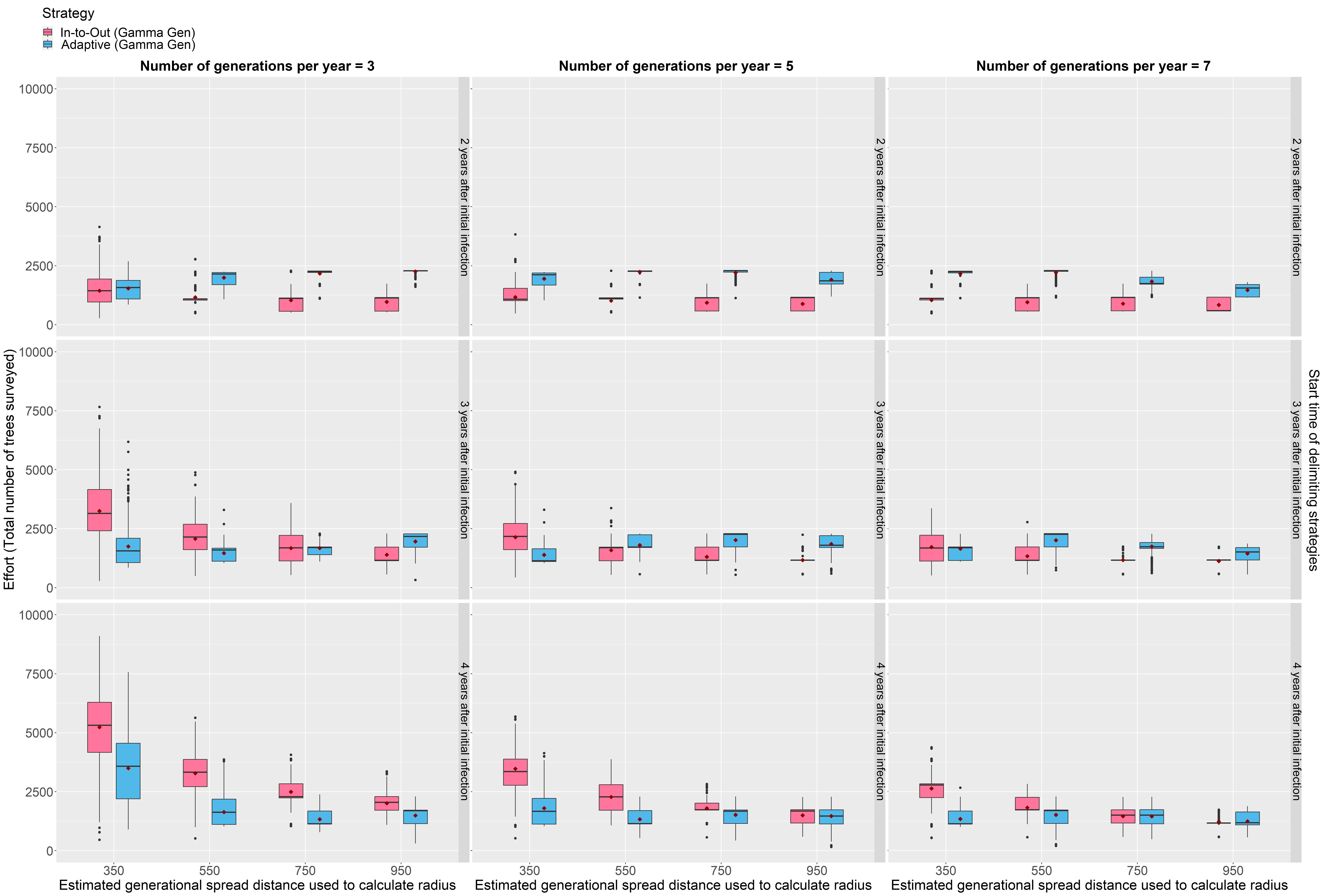

Supplement: Supplementary file 15 — Supplementary Material 15 [file 41598_2025_90343_MOESM15_ESM.jpg]

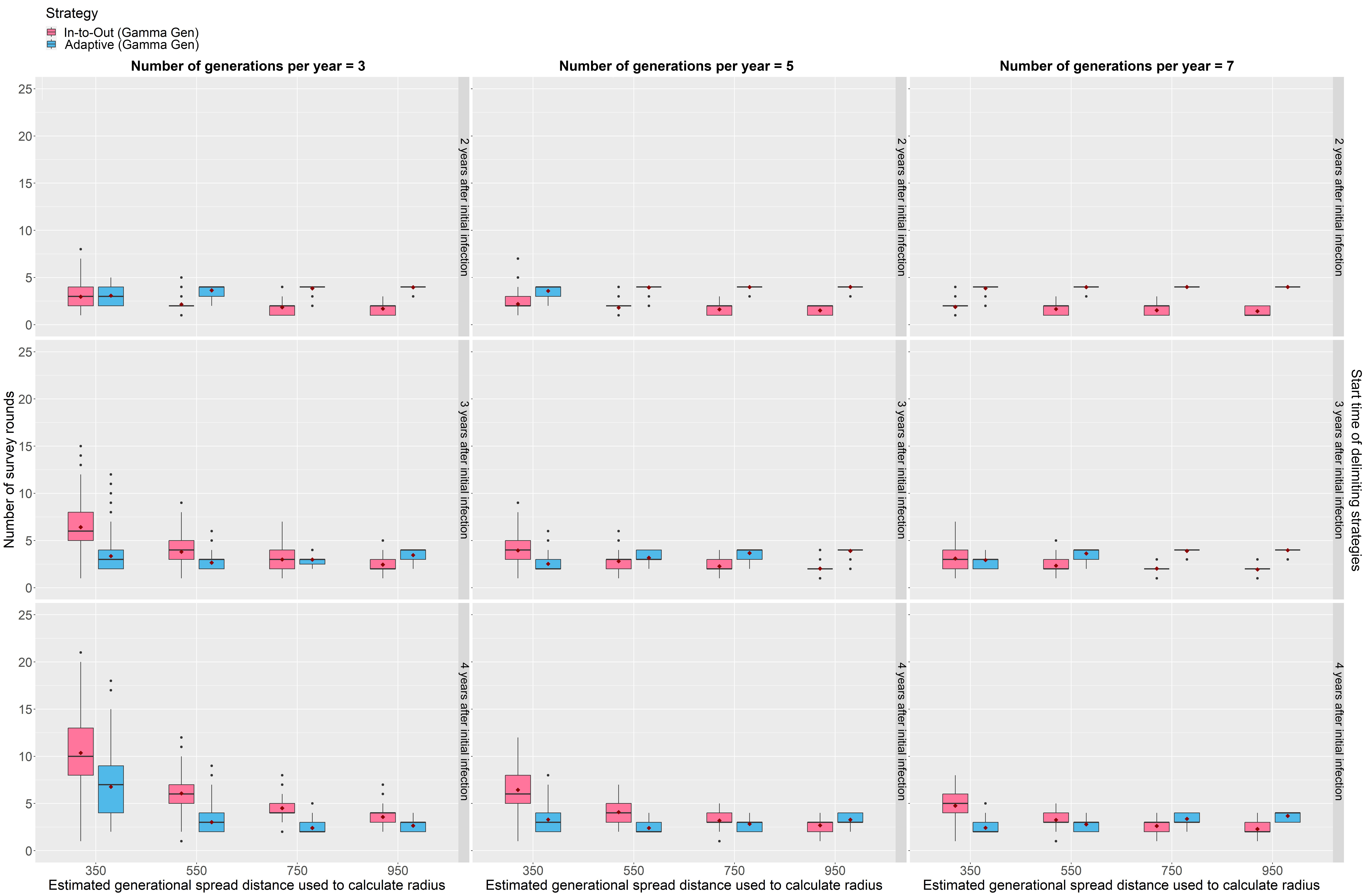

Supplement: Supplementary file 16 — Supplementary Material 16 [file 41598_2025_90343_MOESM16_ESM.jpg]

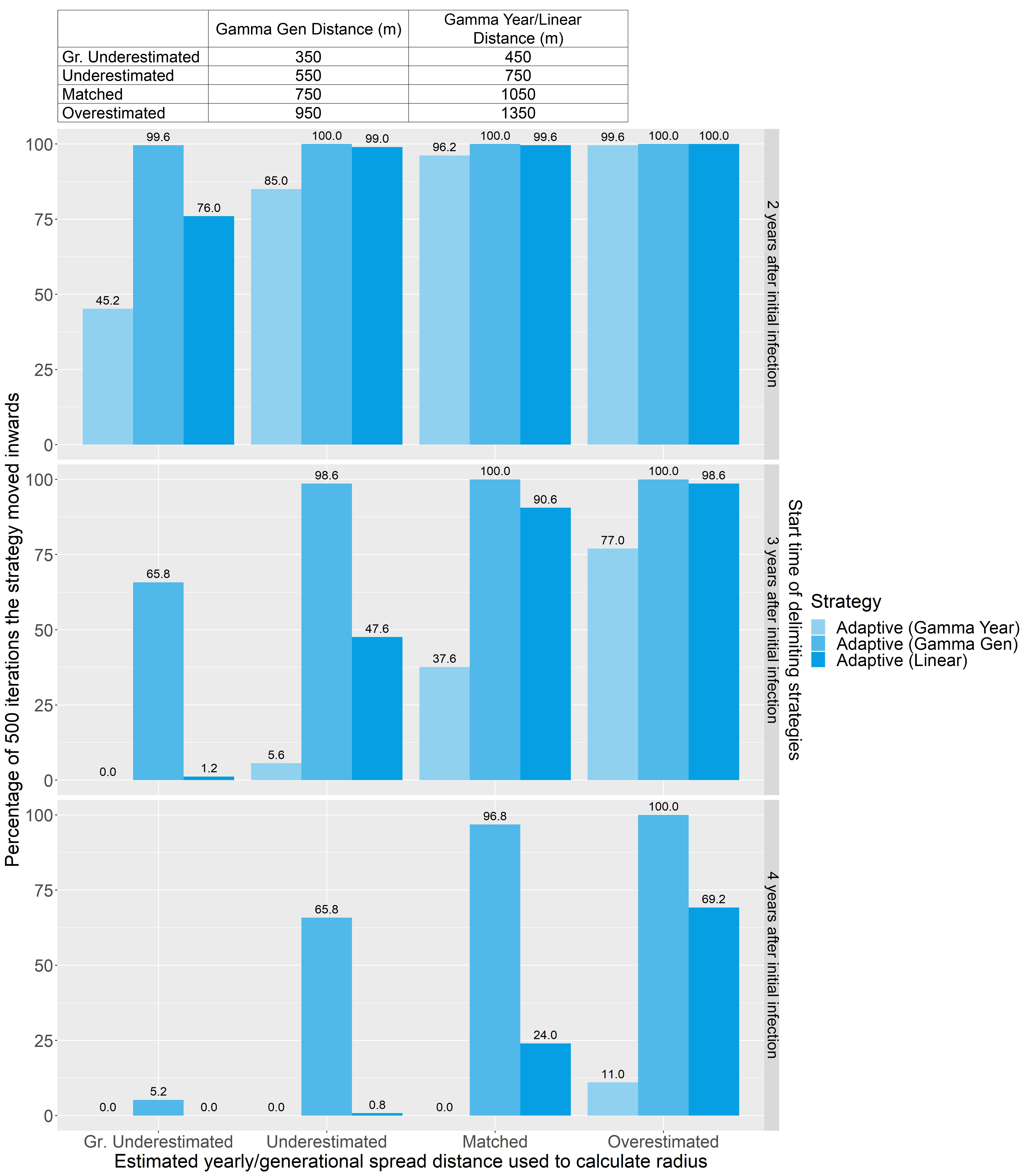

Supplement: Supplementary file 22 — Supplementary Material 22 [file 41598_2025_90343_MOESM22_ESM.jpg]

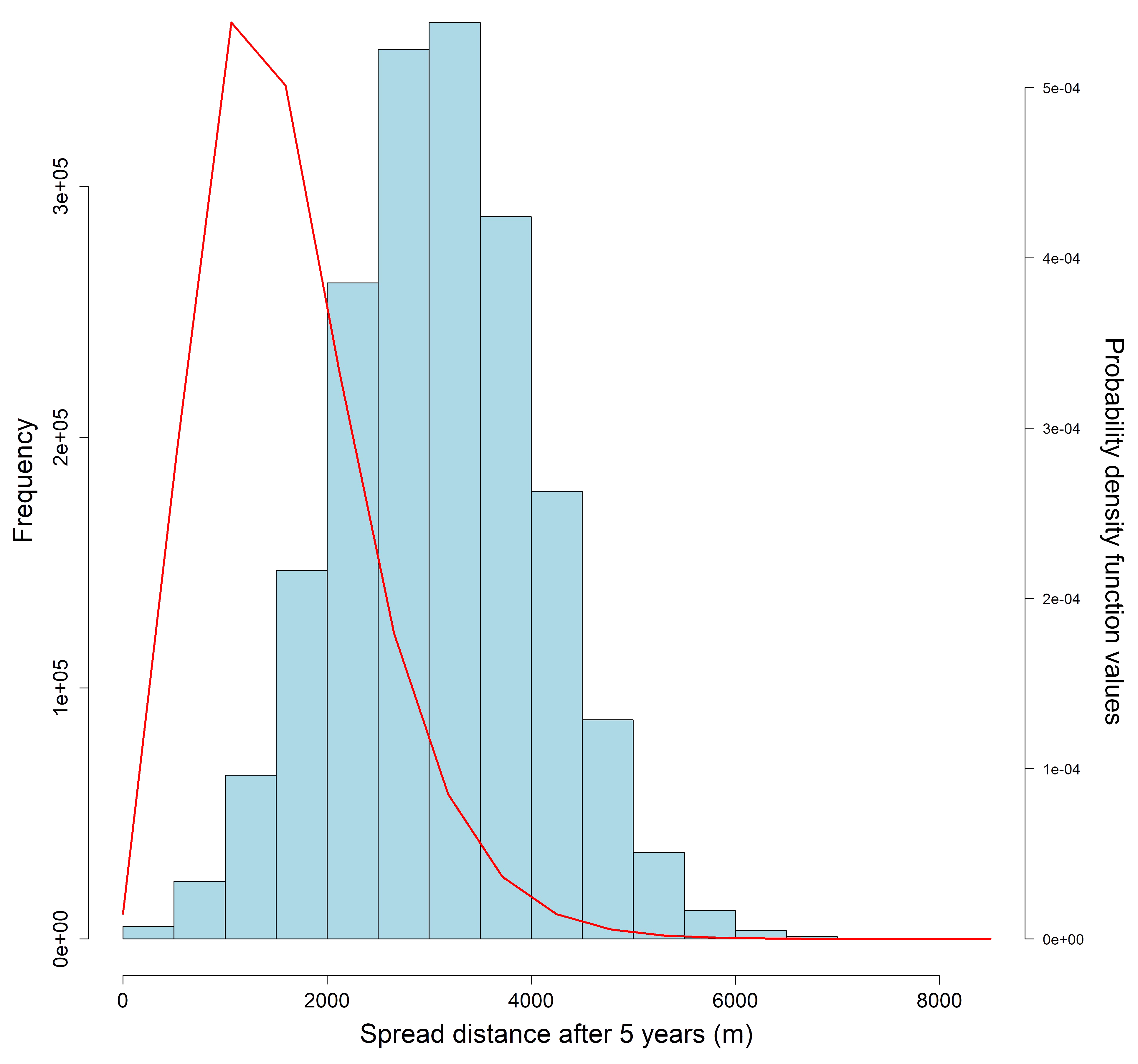

Supplement: Supplementary file 23 — Supplementary Material 23 [file 41598_2025_90343_MOESM23_ESM.jpg]

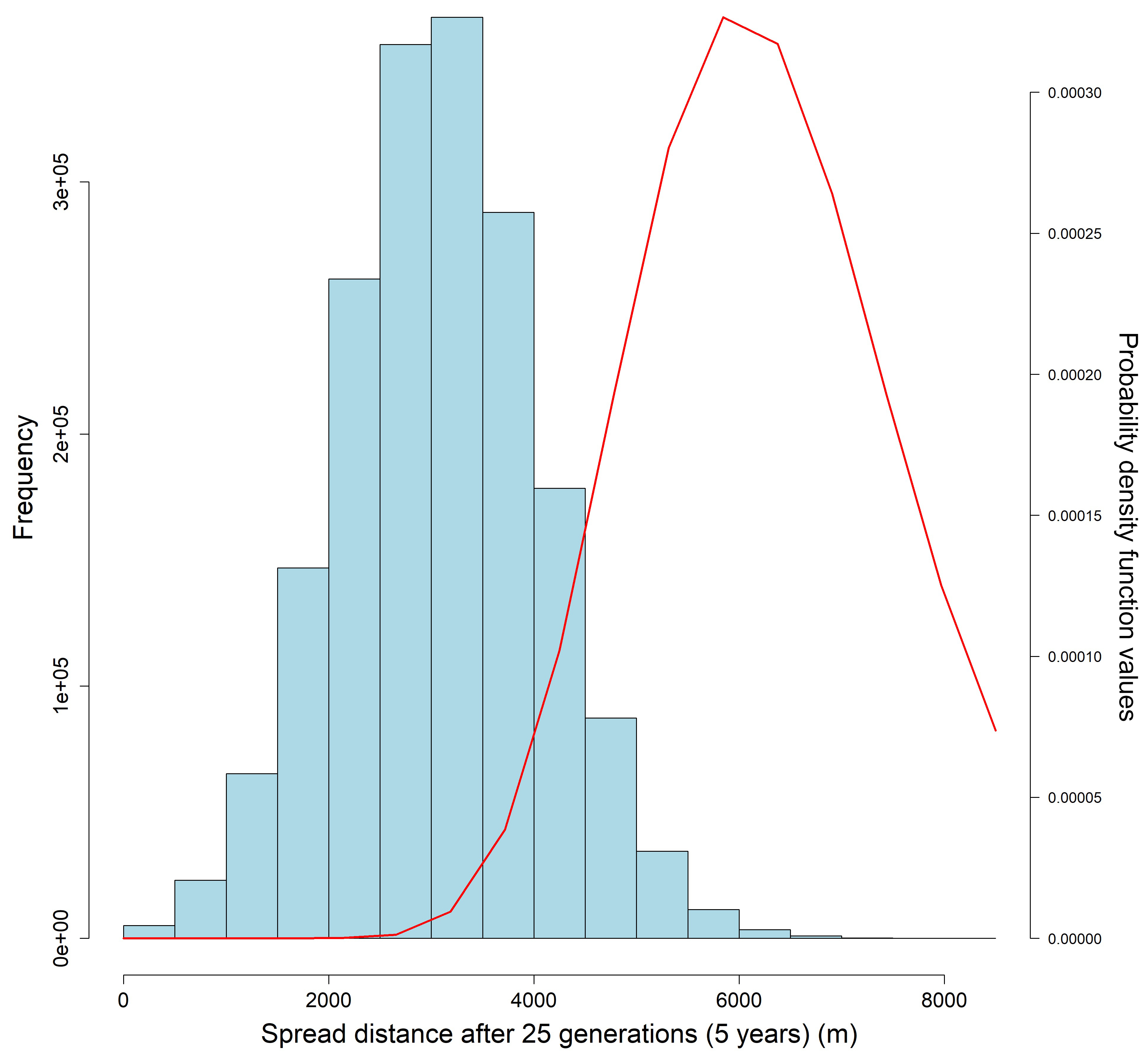

Supplement: Supplementary file 24 — Supplementary Material 24 [file 41598_2025_90343_MOESM24_ESM.jpg]

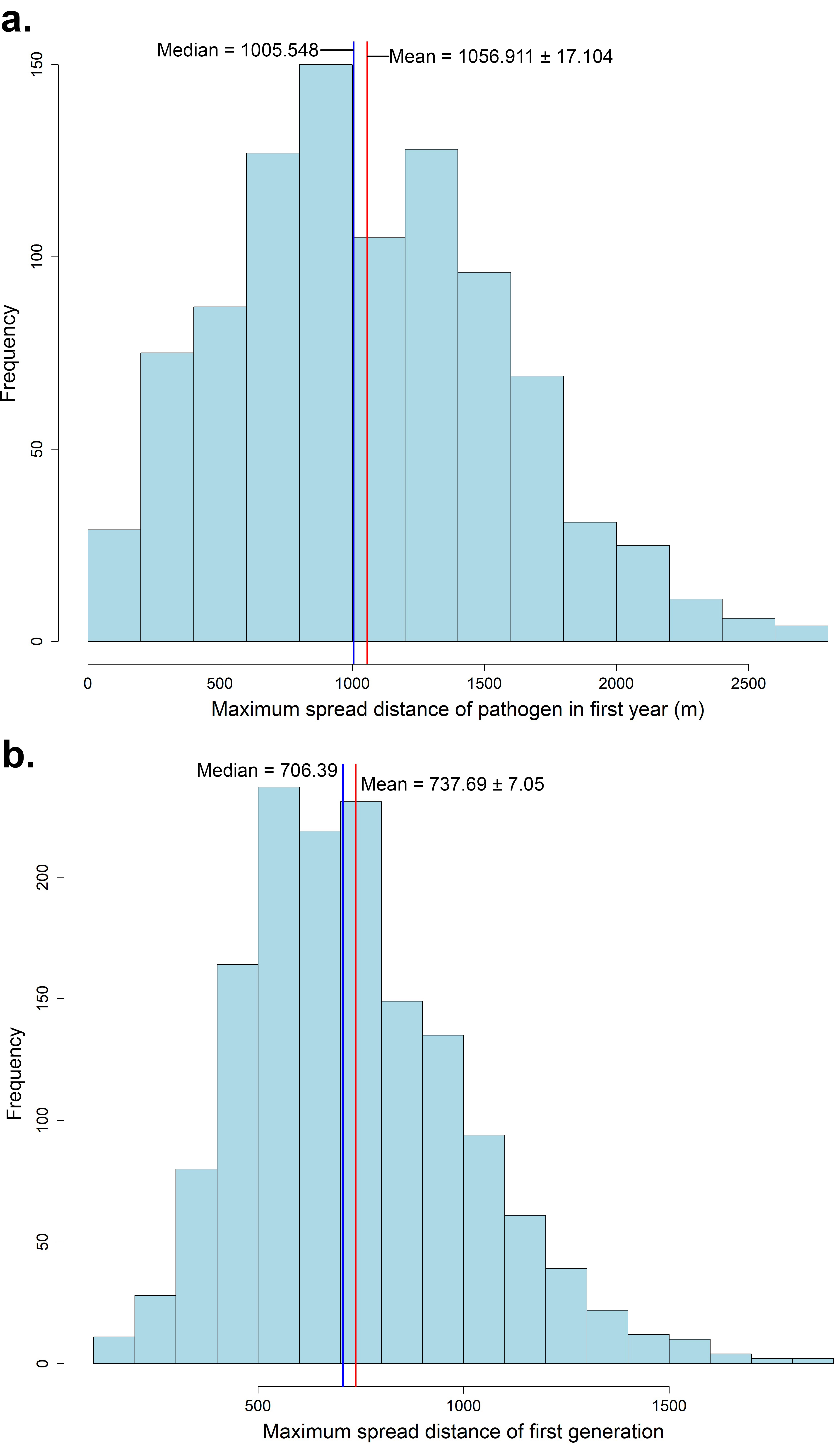

Supplement: Supplementary file 25 — Supplementary Material 25 [file 41598_2025_90343_MOESM25_ESM.jpg]

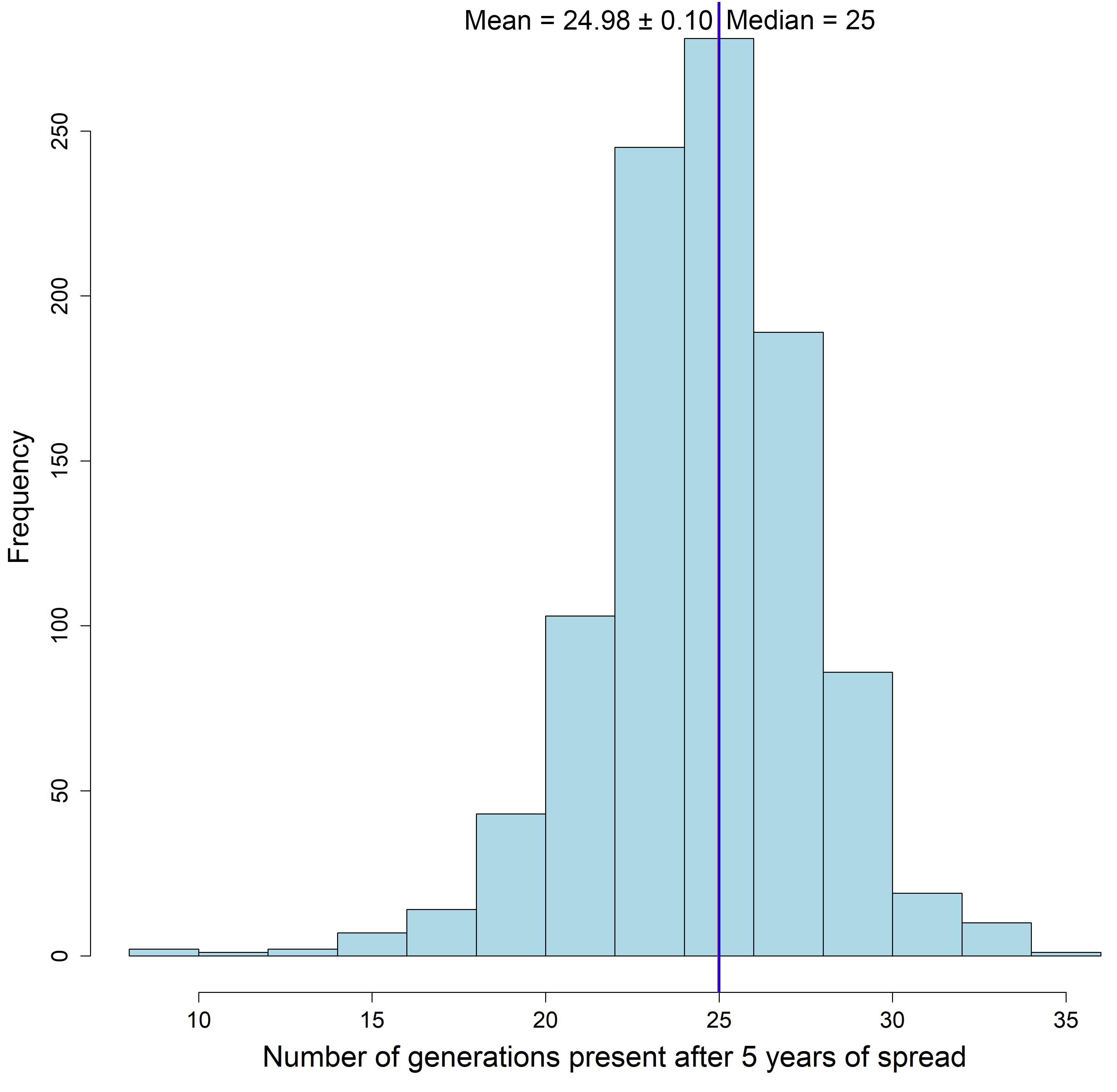

Supplement: Supplementary file 26 — Supplementary Material 26 [file 41598_2025_90343_MOESM26_ESM.jpg]

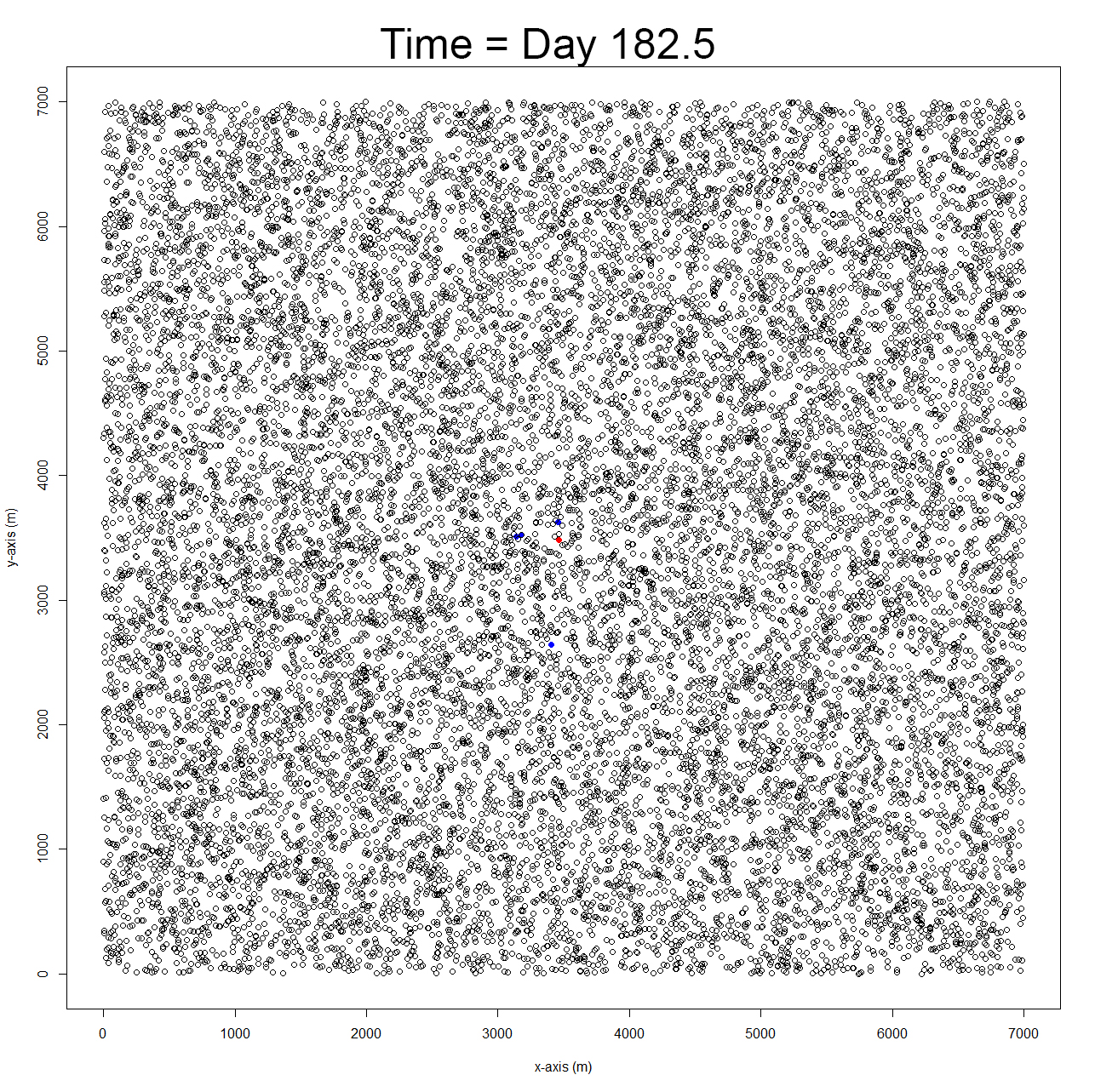

Supplement: Supplementary file 27 — Supplementary Material 27 [file 41598_2025_90343_MOESM27_ESM.gif]

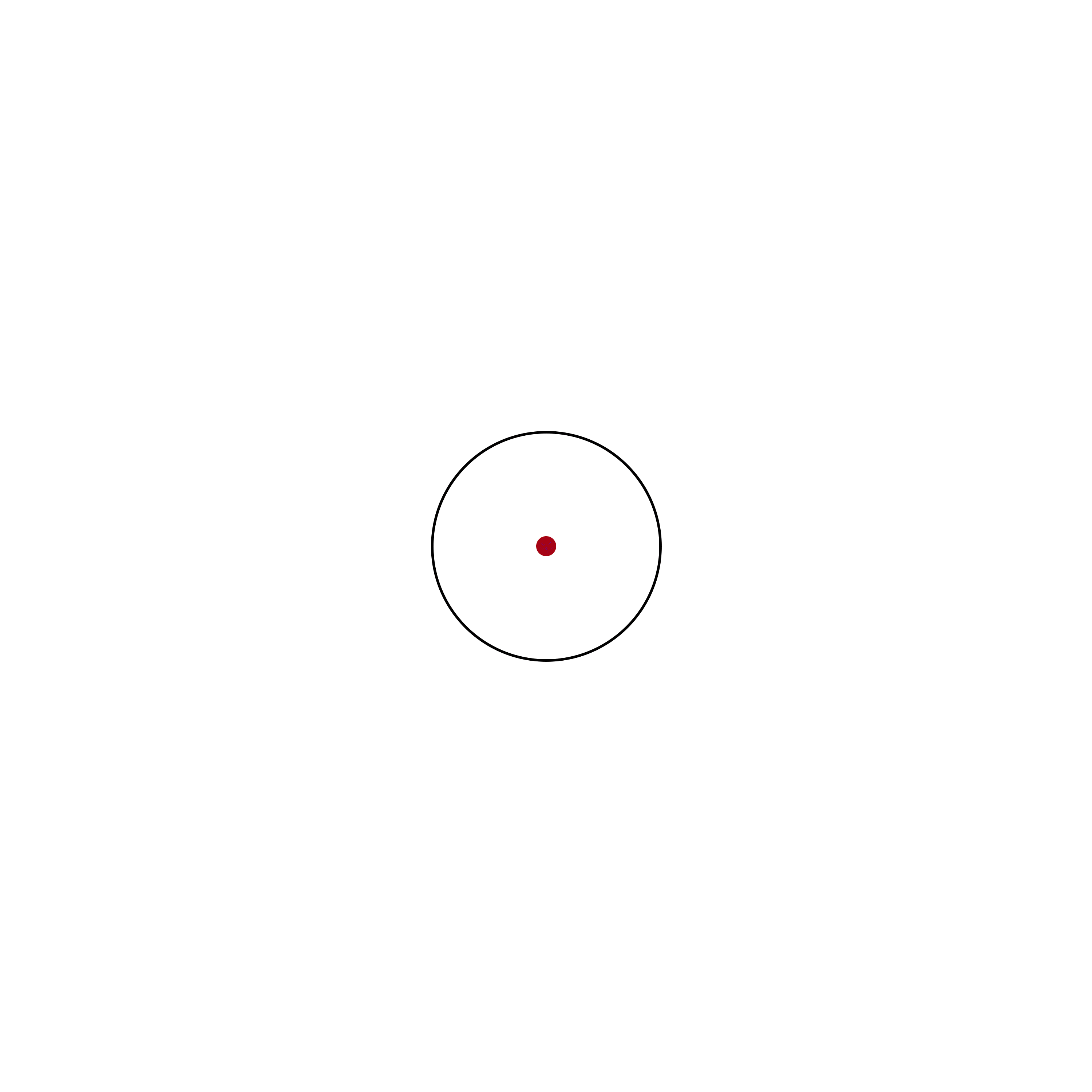

Supplement: Supplementary file 28 — Supplementary Material 28 [file 41598_2025_90343_MOESM28_ESM.gif]

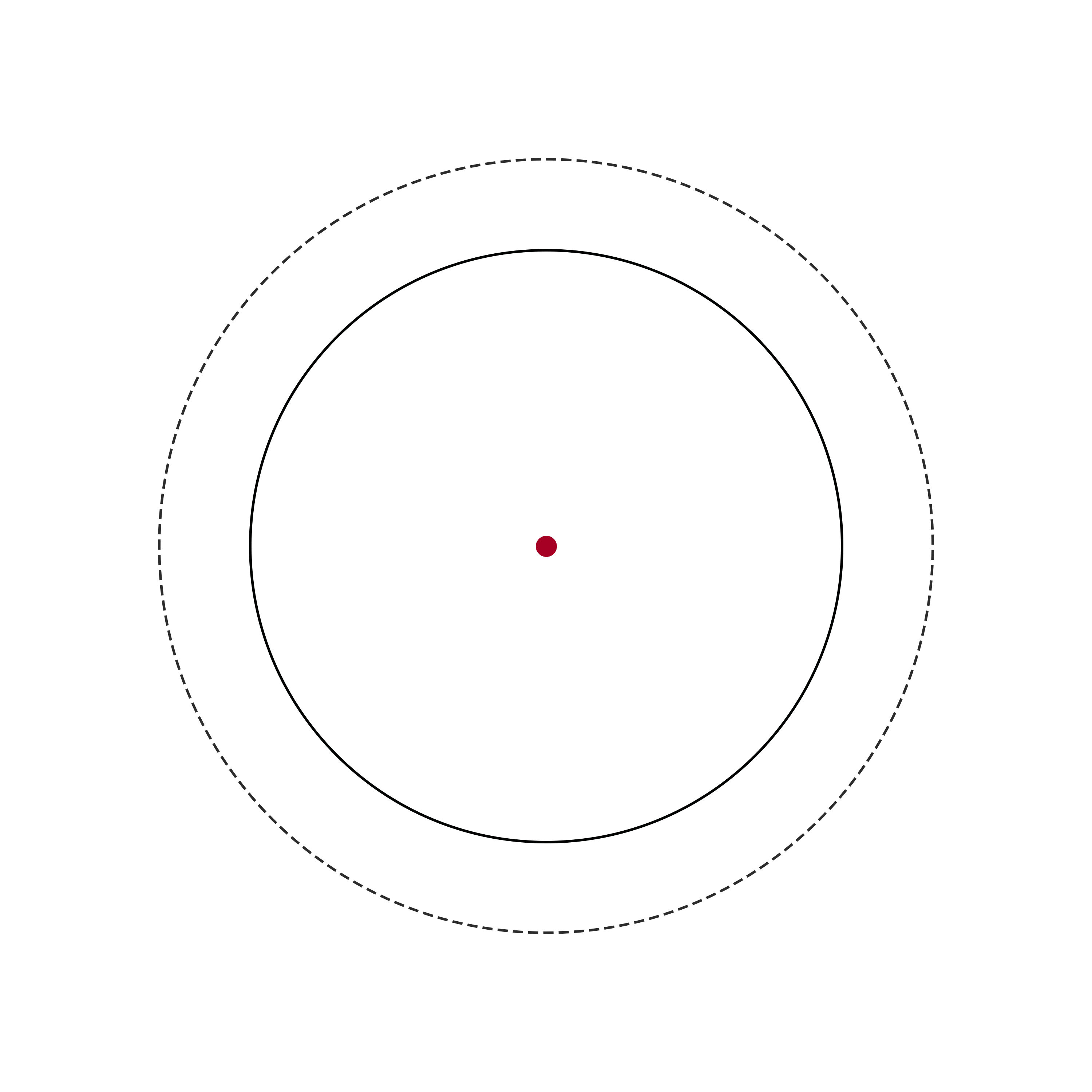

Supplement: Supplementary file 29 — Supplementary Material 29 [file 41598_2025_90343_MOESM29_ESM.gif]

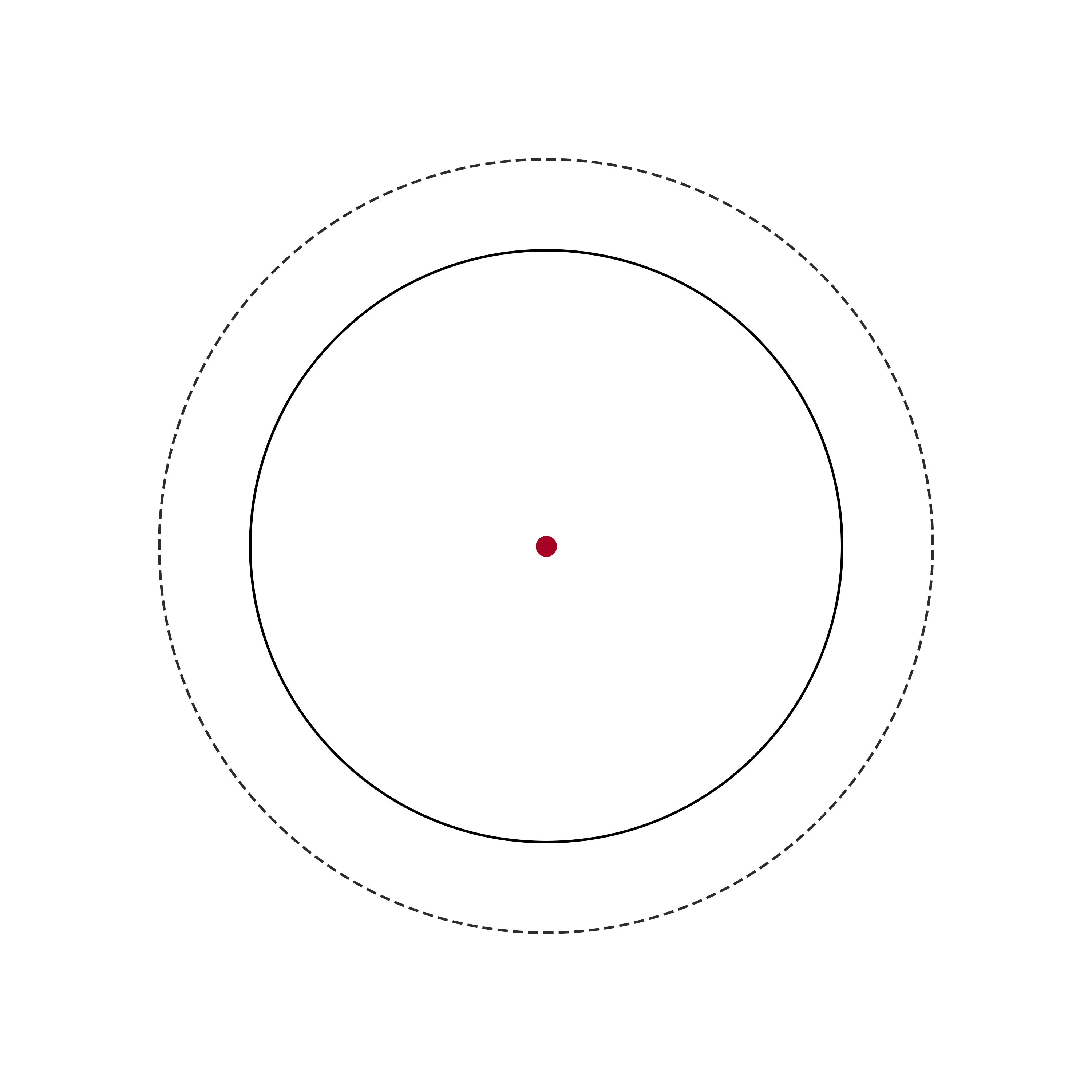

Supplement: Supplementary file 30 — Supplementary Material 30 [file 41598_2025_90343_MOESM30_ESM.gif]

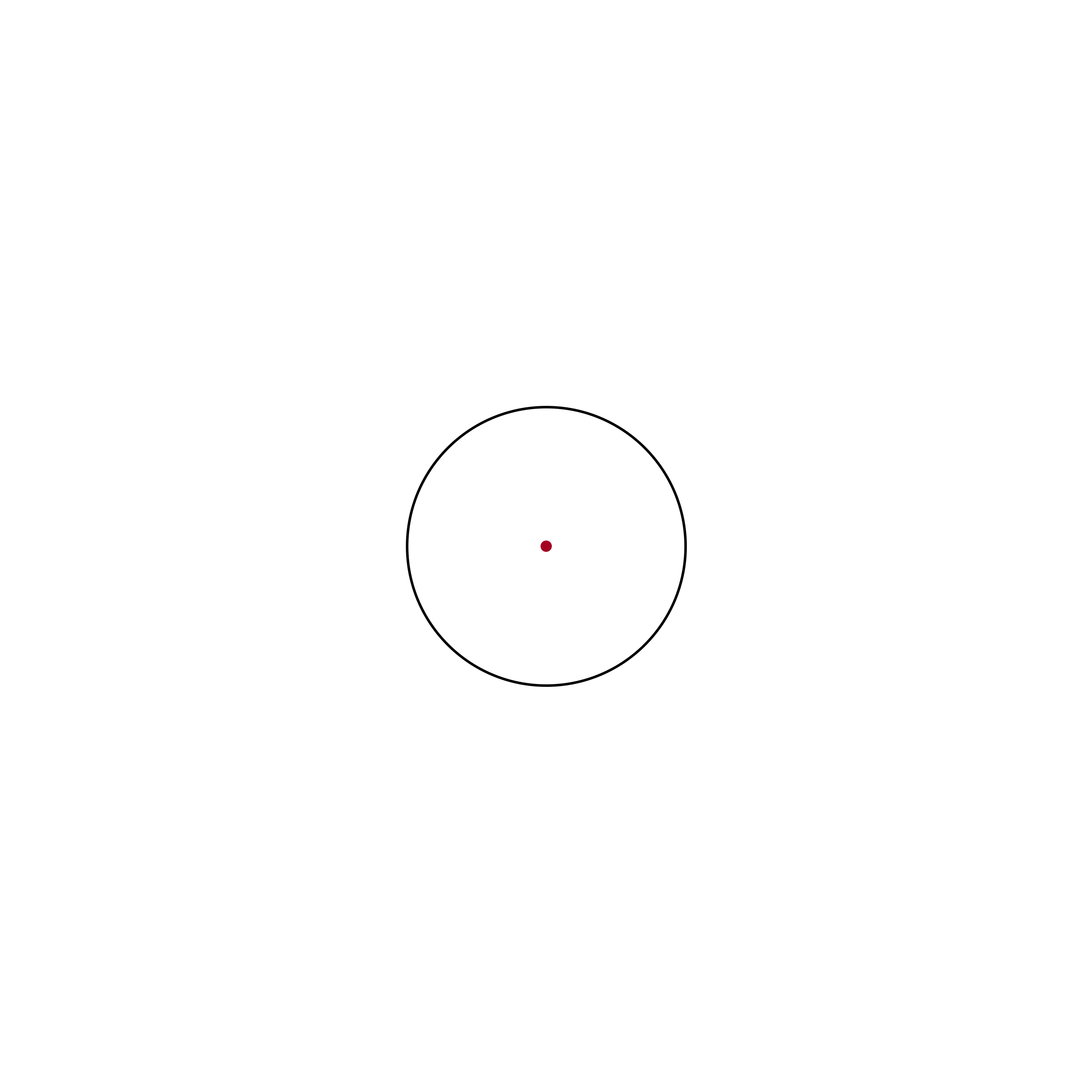

Supplement: Supplementary file 31 — Supplementary Material 31 [file 41598_2025_90343_MOESM31_ESM.gif]

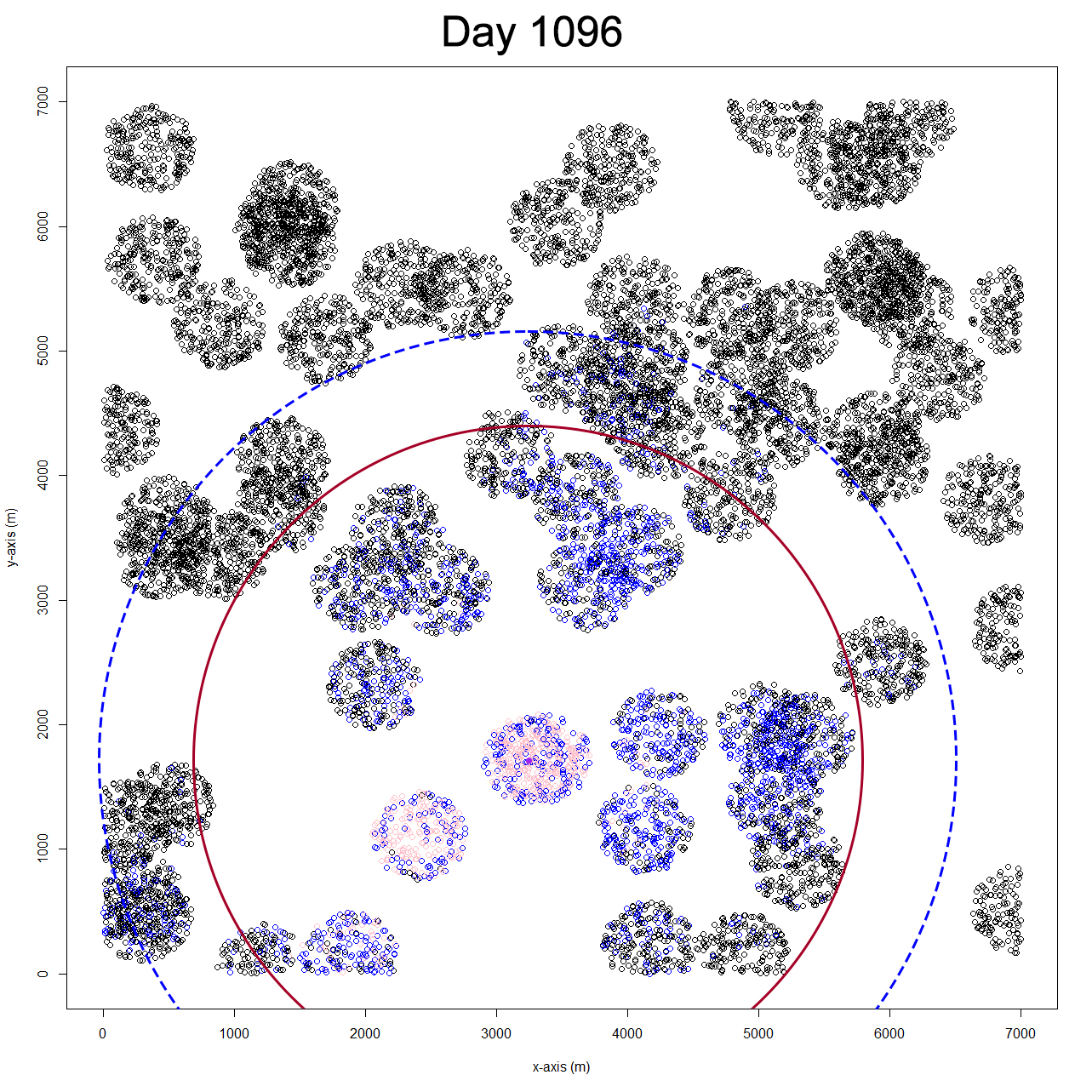

Supplement: Supplementary file 32 — Supplementary Material 32 [file 41598_2025_90343_MOESM32_ESM.gif]

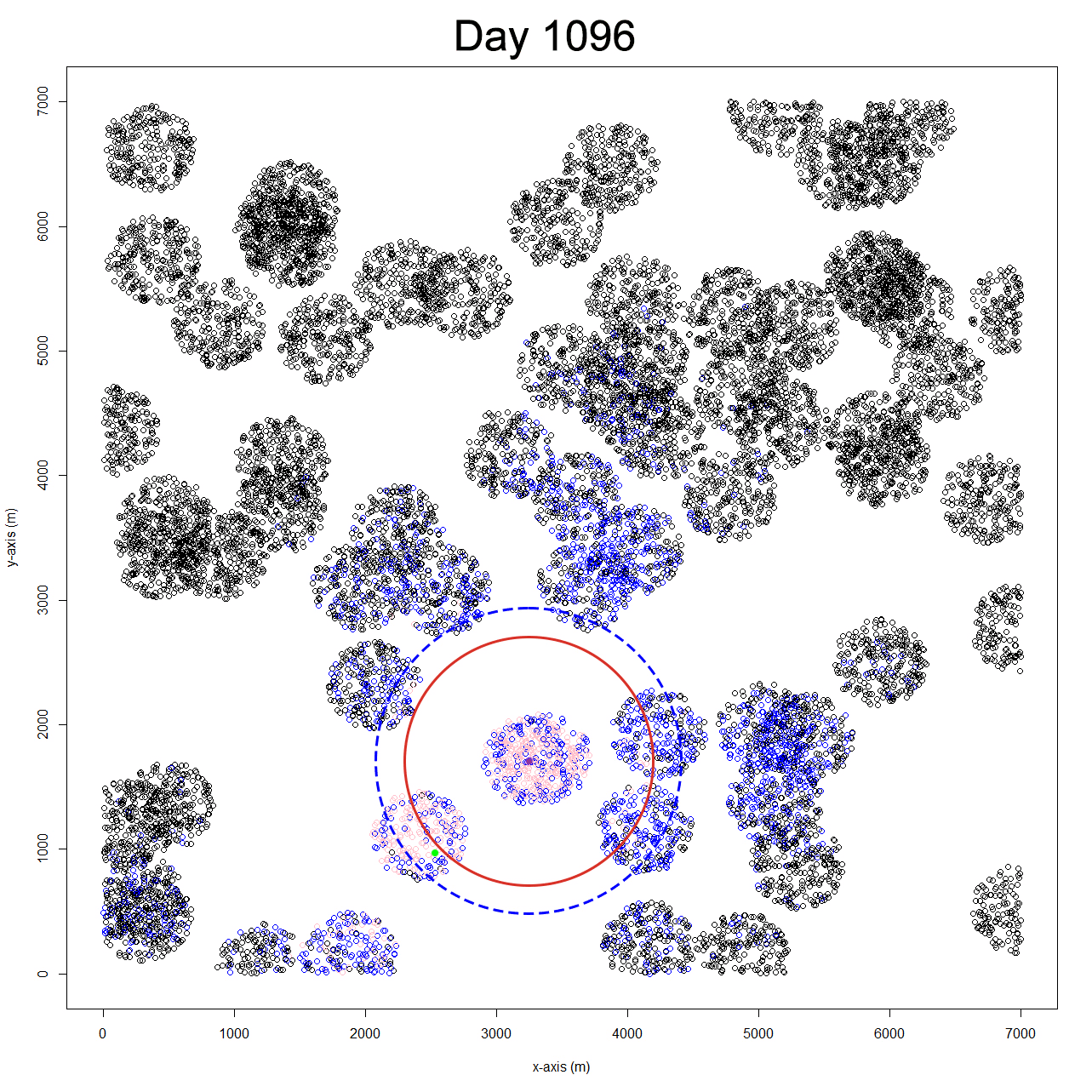

Supplement: Supplementary file 33 — Supplementary Material 33 [file 41598_2025_90343_MOESM33_ESM.gif]

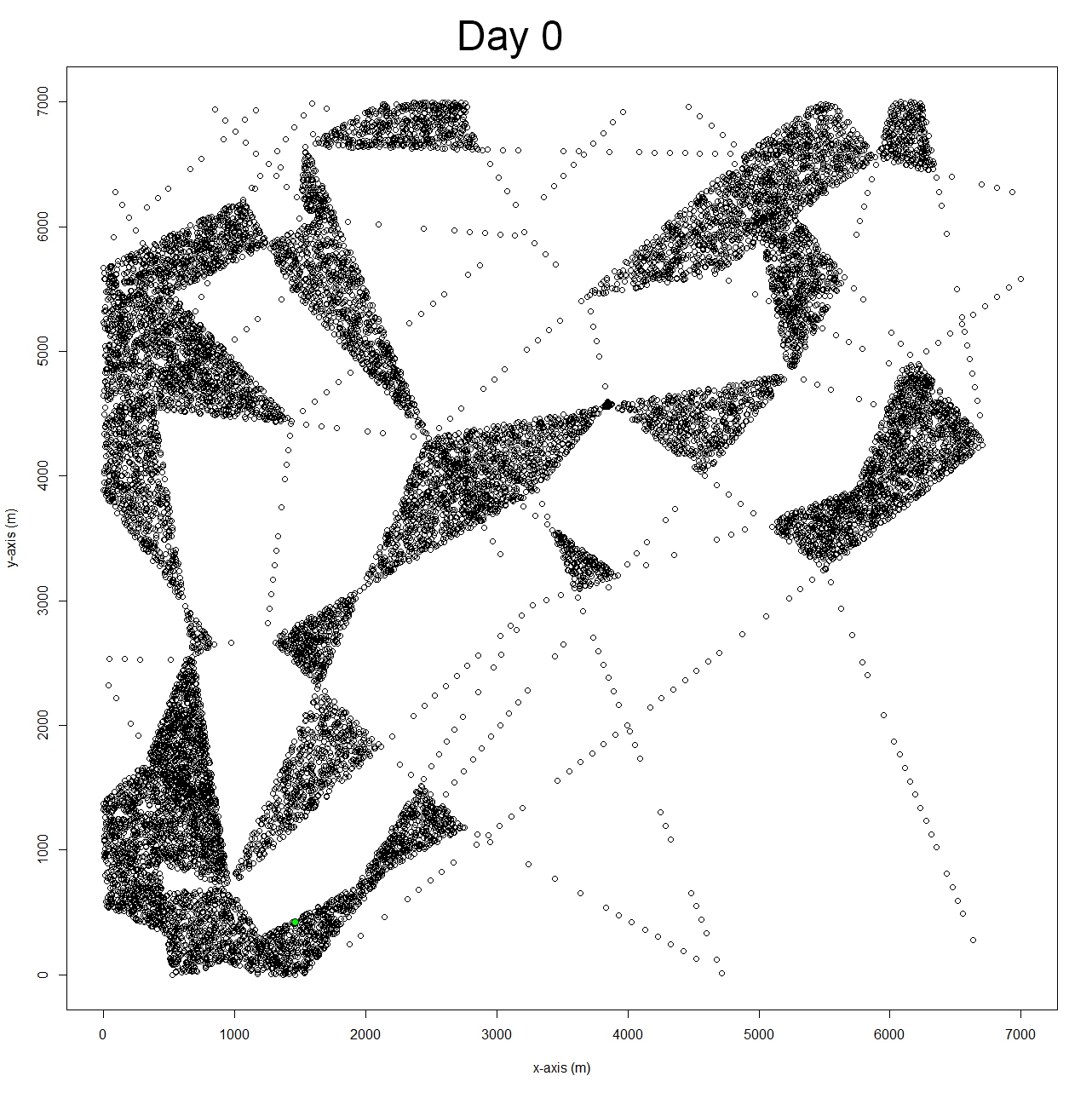

Supplement: Supplementary file 34 — Supplementary Material 34 [file 41598_2025_90343_MOESM34_ESM.gif]

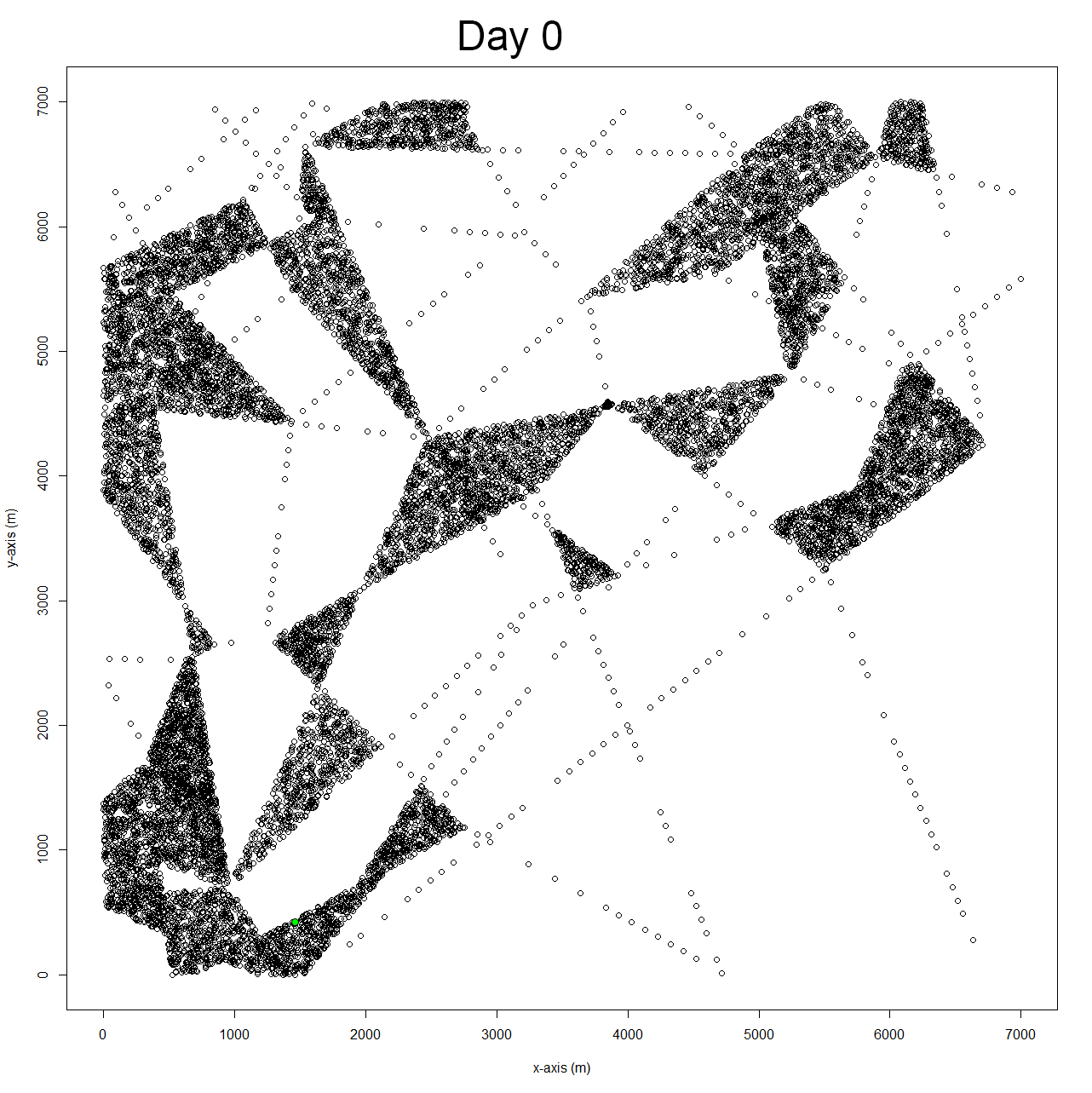

Supplement: Supplementary file 35 — Supplementary Material 35 [file 41598_2025_90343_MOESM35_ESM.gif]
